# Associated morphometric and geospatial differentiation among 98 species of stone oaks (*Lithocarpus*)

**DOI:** 10.1371/journal.pone.0199538

**Published:** 2018-06-26

**Authors:** Xi Chen, Takashi S. Kohyama, Charles H. Cannon

**Affiliations:** 1 School of Ecology and Environment Sciences, Yunnan University, Kunming, Yunnan, China; 2 Faculty of Environmental Earth Science, Hokkaido University, Kita-ku, Sapporo, Hokkaido, Japan; 3 The Center for Tree Science, The Morton Arboretum, Lisle, IL, United States of America; Helmholtz Centre for Environmental Research - UFZ, GERMANY

## Abstract

Two fruit types can be distinguished among stone oaks (*Lithocarpus*) species: the ‘acorn’ (AC) and the ‘enclosed receptacle’ (ER) types. Our morphometric analysis of 595 nuts from 98 species (one third of all *Lithocarpus* spp.) found substantial transition in mechanical protection of the seed between two woody fruit tissues (exocarp and receptacle) of two fruit types. AC fruits were smaller in seed and fruit size and the thin brittle exocarp largely enclosed the seed, whereas ER fruits were larger and the seed was mostly enclosed by thick woody receptacle tissue. The differences in these two tissues were considerably greater between compared to within fruit type and species. Geospatial distribution showed that seed size of all examined species increased with elevation and decreased with latitude, the physical defense increased with both elevation and latitude, and ER-fruit species were more common at higher elevation. The two fruit types represent distinct suites of associated traits that respond differently to the various biotic and abiotic factors associated with geographic variation, profoundly impacting the evolution of the two fruit types. The co-occurrence of two fruit types in the same forest could be a consequence of distinct fruit and animal interactions.

## Introduction

The stone oaks (*Lithocarpus* Blume) are remarkable floristic components of Asian tropical and subtropical forests [1,2]. Do not tolerate freezing temperatures, these evergreen trees can dominate forest canopy, particularly in tropical montane and subtropical forests. As the second largest genus of Fagaceae, over 300 *Lithocarpus* species are distributed from far eastern India through southern China, throughout Indochina, north to southern Japan, and extend through the Malayan archipelago into Papua New Guinea [1,2]. Stone oaks exhibit a wider range of variation in fruit morphology than temperate oks (*Quercus* L.), and the majority of the key traits for identification are in the fruit morphology, because little variation exists in the floral and sterile structures [1–4]. While only a few *Lithocarpus* species produce edible seeds and are actively harvested by humans, many species of stone oaks provide considerable food for forest vertebrates.

**Fig 1:**
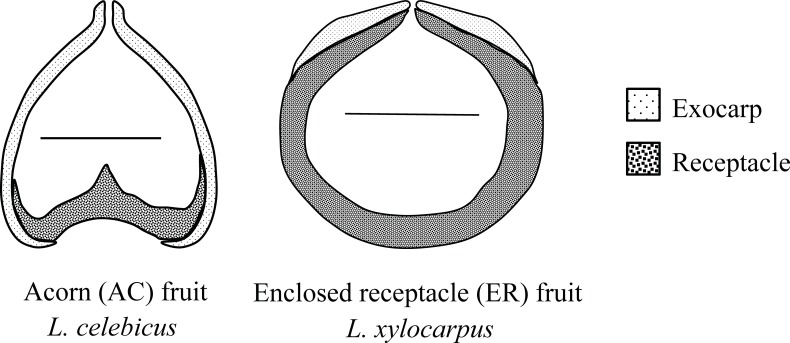
The longitudinal section of two fruit types of *Lithocarpus*. Bars inside of the fruits represent 1cm size reference.

The fruit of *Lithocarpus* consists of a dry nut subtended by a cupule [5,6] (S1 Fig). The cupule is a reduced and modified sterile branching structure with various shapes and textures, protecting nuts during development [7]. The nut consists of one seed enclosed in a fruit wall composed by exocarp and receptacle [4,7,8] (Fig 1). The laminate exocarp consisting of regular, columnar cells that form the outer layer of pericarp [9]. The receptacle is internal to the concaved abscission layer between the seed and the cupule [4]. The morphology of the mature receptacle tissue varies across species, particularly in the degree to which it encloses the seed (Fig 1).

By combining a morphometric analysis with molecular phylogeny, Cannon and Manos [6] identified two fruit types, ‘acorn’ (AC) and ‘enclosed receptacle’ (ER), among 21 Bornean *Lithocarpus* species. Similar to a temperate oak acorn, the seed of an AC fruit is primarily enclosed by the exocarp tissue with the relative small, flattened, and typically concave receptacle at the bottom of the seed (Fig 1). In an ER fruit, the majority of the seed is enclosed by the receptacle tissue, which often becomes woody and thickened, while the exocarp tissue becomes greatly reduced and vestigial. The authors suggested that ER fruit type may have evolved independently twice or more times from different acorn-like ancestors [4]. Chen, Cannon and Conklin-Brittan [8] recognized seed macronutrient and antifeedants trade-off between the two fruit types. There is a higher level of antifeedants in AC seeds as chemical defense, whereas ER seeds are characterized by a higher level of macronutrients. Despite these findings, there remain many unresolved questions on the evolution of two fruit types.

**Fig 2:**
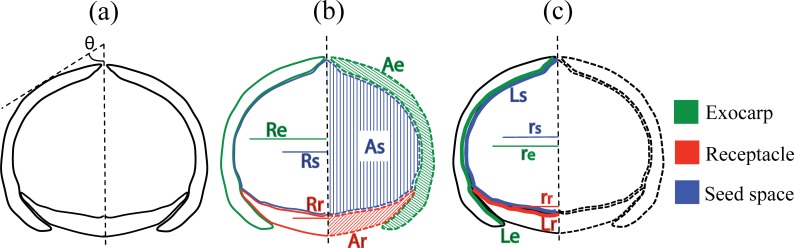
Exocarp rotation dimension and twelve morphological parameters. (a) The rotation angle of exocarp (*θ* in degree) is directly measured. (b) Parameters for estimating the volume dimension of three parts (‘e’ for exocarp, ‘r’ for receptacle and ‘s’ for seed space): *A*_e_, *A*_r_, and *A*_s_ are the area of the three parts on the left side of the longitudinal section; and *R*_e_, *R*_r_, and *R*_s_ are the distance between the rotation axis and the centroid of *A*_e_, *A*_r_ and *A*_s_ respectively. (c) Parameters for estimating the coverage and surface area dimension of three parts (‘e’ for exocarp, ‘r’ for receptacle and ‘s’ for seed space): *L*_s_, *L*_e_, and *L*_r_ are length of seed space and the internal lengths of exocarp and receptacle respectively; and *r*_s_, *r*_e_, and *r*_r_ are the distance traveled by the centroid of *L*_s_, *L*_e_, and *L*_r_ respectively.

The previous studies [4,8] applied visual identification for fruit type identification, but quantifying and comparing the seed coverage level by exocarp and receptacle tissue would be more precise for distinguishing two fruit types. As chemical composition of seeds could be associated with the degree of physical protection [8], examining the mechanical defense by exocarp and receptacle could clarify the distinctions of defensive mechanisms between two fruit types. Moreover, besides the degree of coverage by exocarp and receptacle [4], such characteristics as fruit size, seed size and exocarp rotation angle (Fig 2) would be important dimensions for characterizing the morphological variation between and within the two fruit types. It is observed that the overall fruit size and seed volume can be greater for ER-fruit species [4], which may reflect greater offspring investment per fruit compared to AC-fruit species. The dimension of the exocarp rotation angle quantifies the overall fruit shape. ER fruits are observed to be rounder than AC fruits, and the exocarp rotation angle would quantify the general fruit shape of two fruit types. By combining the variation of exocarp and receptacle with these detailed morphological dimensions, the inter- and intraspecific variation in *Lithocarpus* fruits could be described, and the evolutionary and functional significance of each dimension could be clarified. Last but not least, comparing the effectiveness of Fourier coefficients and our morphometric dimensions could provide valuable reference for future fruit morphometric analysis of the stone oaks and taxa with similar fruit morphology.

There are a number of factors that determine the geographic distribution of plant species: the ecological, physiological and anatomical traits over life history stages, the phenotypic and genotypic variation in these traits, and the evolutionary and biogeographic history of the taxon [10]. Among them, traits related to seed dispersal and establishment are particularly important in geographic distribution [11]. As abundant canopy tree species, stone oaks with outstanding species richness are distributed over a wide geographic range in East Asia. Together with divergent fruit morphology, these facts make this genus an ideal model for examining speciation and radiation. In this article, we link the variation in fruit type, seed size and mechanistic seed defense with geographic distribution across species of *Lithocarpus*, and discuss possible selective forces behind fruit type evolution and diversification.

## Materials and methods

### Sampling design and fruit image preparation

In total, 595 mature fruits of 98 species were applied in the study (Table 1 and S1 Table). The majority of the fruit specimens were collections from the National Herbarium Netherlands, the Harvard University Herbaria, and the Herbarium of Kunming Institute of Botany of the Chinese Academy of Sciences. The rest of the fruit specimens were field collections from the Hengduan Mountains region of China. All the species names were confirmed to remove synonyms. To ensure the maturity of the fruit samples, size comparisons were made between our fruit samples and fruits from other specimens. In case where fruits from other specimens were not available, apparent fully-developed seeds were assumed to indicate the fruit maturity. The dissected taxa represent about 1/3 of the recorded *Lithocarpus* species, which encompass a wide range of morphological variation and geographic distribution.

**Table 1:** The fruit type classification based on the species average seed coverage by exocarp and receptacle.

Fruit type	Species	Sample number	*S*_s_ (cm^2^)	*S*_e_ (cm^2^)	*S*_r_ (cm^2^)
AC	*Lithocarpus bacgiangensis* (Hickel & A. Camus) A. Camus	3	8.46	8.39	4.70
AC	*L*. *bancanus* (Scheff.) Rehder	2	13.97	10.71	3.35
AC	*L*. *bennettii* (Miq.) Rehder	5	5.04	4.41	0.93
AC	*L*. *blumeanus* (Korth.) Rehder	4	10.04	7.81	2.26
AC	*L*. *brachystachyus* Chun	1	4.77	4.79	1.02
AC	*L*. *brevicaudatus* (Skan) Hayata	1	8.86	8.29	1.85
AC	*L*. *calolepis* Y.C. Hsu & H. Wei Jen	5	13.17	12.02	5.67
AC	*L*. *calophyllus* Chun ex. C.C. Hunag & Y.T.Chang	1	6.88	6.43	2.25
AC	*L*. *carolinae* (Skan ex Dunn) Rehder	1	13.80	13.01	4.29
AC	*L*. *caudatilimbus* (Merr.) A. Camus	1	11.80	10.81	1.02
AC	*L*. *celebicus* (Miq.) Rehder	5	8.61	7.81	1.10
AC	*L*. *chrysocomus* Chun & Tsiang	1	5.80	4.03	2.20
AC	*L*. *confertus* Soepadmo	3	7.40	6.09	1.17
AC	*L*. *confinis* S.H. Huang ex Y.C. Hsu & H.W. Jen	7	5.66	5.75	1.35
AC	*L*. *conocarpus* (Oudem.) Rehder	1	11.56	9.99	1.37
AC	*L*. *craibianus* Barnett	38	4.77	4.30	1.23
AC	*L*. *crassinervius* (Blume) Rehder	2	12.69	13.86	1.62
AC	*L*. *cryptocarpus* A. Camus *	1	27.20	32.78	30.24
AC	*L*. *cyclophorus* (Endl.) A. Camus	3	34.99	26.19	24.71
AC	*L*. *dasystachyus* (Miq.) Rehder	3	4.15	3.96	0.36
AC	*L*. *dealbatus* (Hook.f. & Thomson ex Miq.) Rehder	23	5.00	4.19	2.27
AC	*L*. *echinotholus* (Hu) H.Y. Chun & Huang ex Y.C. Hsu & H.W. Jen	3	10.09	8.90	2.10
AC	*L*. *edulis* (Makino) Nakai	8	7.64	7.06	1.83
AC	*L*. *elegans* (Blume) Hatus. ex Soepadmo	24	8.74	8.26	3.00
AC	*L*. *elmerrillii* Chun	4	6.43	5.33	0.99
AC	*L*. *encleisocarpus* (Korth.) A. Camus	4	7.01	7.45	0.83
AC	*L*. *ewyckii* (Korth.) Rehder	1	15.58	14.10	2.79
AC	*L*. *farinulentus* (Hance) A. Camus	4	3.85	3.26	0.63
AC	*L*. *fenestratus* (Roxb.) Rehder	19	6.63	5.75	1.35
AC	*L*. *ferrugineus* Soepadmo	10	7.02	5.90	1.23
AC	*L*. *fohaiensis* (Hu) A. Camus	6	8.81	7.30	2.88
AC	*L*. *formosanus* (Skan) Hayata	2	6.86	7.49	1.54
AC	*L*. *glaber* (Thunb.) Nakai	12	5.40	5.56	0.46
AC	*L*. *glutinosus* (Blume) Soepadmo	1	10.35	7.52	2.60
AC	*L*. *gracilis* (Korth.) Soepadmo	9	7.67	5.94	1.40
AC	*L*. *hancei* (Benth.) Rehder	75	7.83	7.58	1.70
AC	*L*. *handelianus* A. Camus	9	12.04	9.12	3.22
AC	*L*. *harlandii* (Hance ex Walp.) Rehder	8	9.31	9.16	1.53
AC	*L*. *henryi* (Seemen) Rehder & E.H. Wilson	5	10.17	9.95	2.26
AC	*L*. *himalaicus* C. C.Huang & Y.T. Chang	4	9.03	8.26	2.24
AC	*L*. *howii* Chun	1	5.09	4.27	0.49
AC	*L*. *hypoglaucus* (Hu) C.C. Huang ex Y.C. Hsu & H.W. Jen	8	8.16	6.86	2.54
AC	*L*. *indutus* (Blume) Rehder *	3	13.12	18.00	12.05
AC	*L*. *jacobsii* Soepadmo	4	14.35	15.57	3.80
AC	*L*. *kawakamii* (Hayata) Hayata	3	14.55	16.68	8.60
AC	*L*. *konishii* (Hayata) Hayata *	8	5.37	7.93	5.57
AC	*L*. *lappaceus* (Roxb.) Rehder	2	6.95	5.47	1.04
AC	*L*. *leptogyne* (Korth.) Soepadmo	5	4.99	4.25	1.20
AC	*L*. *lindleyanus* (Wall. ex A. DC.) A. Camus	1	5.51	6.20	1.65
AC	*L*. *litseifolius* (Hance) Chun	16	6.93	6.78	2.18
AC	*L*. *longanoides* C.C. Huang & Y.T. Chang	1	6.11	5.15	0.76
AC	*L*. *longipedicellatus* (Hickel & A. Camus) A. Camus	3	5.78	5.36	1.03
AC	*L*. *lucidus* (Roxb.) Rehder	1	9.01	11.26	8.86
AC	*L*. *luteus* Soepadmo	1	6.74	6.79	1.88
AC	*L*. *mairei* (Schottky) Rehder	10	4.91	4.15	0.71
AC	*L*. *meijeri* Soepadmo	1	9.21	6.92	1.80
AC	*L*. *naiadarum* (Hance) Chun	10	7.57	6.65	0.92
AC	*L*. *nieuwenhuisii* (Seemen) A. Camus	1	9.13	9.88	0.32
AC	*L*. *nodosus* Soepadmo	1	10.02	9.80	2.26
AC	*L*. *oblanceolatus* C.C. Huang & Y.T. Chang	3	10.73	8.46	2.05
AC	*L*. *obscurus* C.C. Huang & Y.T. Chang	4	7.37	7.02	1.62
AC	*L*. *pallidus* (Blume) Rehder	2	34.76	30.75	13.27
AC	*L*. *petelotii* A. Camus	1	10.50	10.32	3.84
AC	*L*. *polystachyus* (Wall. ex A. DC.) Rehder	16	5.10	5.04	1.30
AC	*L*. *pseudokunstleri* A. Camus	2	9.55	9.44	0.32
AC	*L*. *pseudomoluccus* (Blume) Rehder	4	26.47	22.90	13.53
AC	*L*. *pseudovestitus* A. Camus	4	3.41	3.76	0.70
AC	*L*. *pusillus* Soepadmo	1	23.14	18.25	10.11
AC	*L*. *rhabdostachyus* (Hickel & A. Camus) A. Camus	2	7.75	6.22	1.84
AC	*L*. *rosthornii* (Schottky) Barnett	3	6.55	5.49	0.69
AC	*L*. *silvicolarum* (Hance) Chun	8	9.68	7.67	2.40
AC	*L*. *skanianus* (Dunn) Rehder	2	8.24	6.94	1.59
AC	*L*. *sundaicus* (Blume) Rehder	2	7.03	5.78	1.36
AC	*L*. *taitoensis* (Hayata) Hayata	7	6.80	6.37	1.74
AC	*L*. *touranensis* (Hickel & A. Camus) A. Camus	2	7.02	5.69	1.88
AC	*L*. *trachycarpus* (Hickel & A.Camus) A. Camus	3	5.76	4.91	0.47
AC	*L*. *vestitus* (Hickel & A. Camus) A. Camus	4	4.21	4.35	1.41
ER	*L*. *amygdalifolius* (Skan) Hayata	4	12.26	8.10	10.37
ER	*L*. *balansae* (Drake) A. Camus	1	11.08	3.34	8.78
ER	*L*. *beccarianus* (Benth.) A. Camus	1	15.67	7.78	15.84
ER	*L*. *cleistocarpus* (Seemen) Rehder & E.H. Wilson	7	7.16	3.71	5.58
ER	*L*. *corneus* (Lour.) Rehder	5	14.15	11.63	12.11
ER	*L*. *damiaoshanicus* C.C. Huang & Y.T. Chang	1	5.24	2.37	4.23
ER	*L*. *fordianus* (Hhemsl.) Chun	2	3.86	3.34	4.02
ER	*L*. *javensis* Blume	7	23.00	5.86	22.52
ER	*L*. *lampadarius* (Gamble) A. Camus *	1	15.05	15.32	16.54
ER	*L*. *laoticus* (Hhickel & A. Camus) A. Camus	1	10.99	1.29	10.28
ER	*L*. *lepidocarpus* (Hayata) Hayata	1	13.80	2.16	14.03
ER	*L*. *megacarpus* Soepodmo	1	19.31	6.34	18.11
ER	*L*. *pachylepis* A. Camus	5	23.92	21.58	23.24
ER	*L*. *pachyphyllus* (Kruz) Rehder	2	30.79	3.30	32.39
ER	*L*. *platycarpus* (Blume) Rehder	5	24.71	18.80	19.67
ER	*L*. *pseudoxizangensis* Z.K. Zhou & H. Sun	2	6.26	3.33	3.60
ER	*L*. *truncatus* (King ex Hook. f.) Rheder	26	5.15	1.61	4.10
ER	*L*. *turbinatus* (Stapf) Forman	2	14.05	8.87	13.78
ER	*L*. *uvariifolius* (Hance) Rehder	5	12.13	10.46	11.24
ER	*L*. *variolosus* (Franch.) Chun	5	8.55	4.43	5.55
ER	*L*. *xylocarpus* (Kurz) Markgr.	42	12.90	5.62	12.42

*S*_s_, *S*_e_ and *S*_r_ are surface area of seed space, and seed-enclosure level by exocarp and receptacle on the species level respectively. When *S*_e_ > *S*_r,_ the fruit is categorized to AC (acorn) fruit type. If *S*_e_ < *S*_r_, the fruit is categorized to ER (enclosed receptacle) fruit type. The species marked with asterisks (*) were highly enclosed by both exocarp and receptacle tissue.

The mature nuts were cut through the stylar column as the longitudinal axis defined the dissection plane for all fruit specimens [4]. Based on the fruit size and the thickness of the fruit wall, the dissecting tools ranged from hand saws, band saws to razor blades. The images of the longitudinal sections were captured by Nikon and Canon digital SLR cameras. A ruler at the side of each dissected fruit was used as a size reference and also for ensuring image fidelity, as some lens at certain magnification can wrap images at their margins. The proximal ends of receptacle were aligned to standardize the fruit orientation, and the longitudinal axis of the fruit was depicted in Photoshop CS5.1.

### Fruit morphological dimensions and parameters

Only one dimension, the rotation angle (*θ* in degree) of the exocarp from the rotation axis was directly measured on the image (Fig 2A and S1 Table). We employed the Pappus-Guldinus Theorem to reconstruct 3D fruit shape and to obtain the coverage and the volume of three fruit parts (seed space, receptacle and exocarp) from the 2D image of longitudinal fruit section, assuming a fruit to be a perfect rotation body. From the left-hand side of the longitudinal axis of fruit images, we measured the following 12 parameters using Image J 1.51h [12]: the section area of exocarp (*A*_e_), receptacle (*A*_r_), and seed space (*A*_s_), the distance from the rotation axis to the centroid of *A*_e_, *A*_r_, and *A*_s_, namely *R*_e_, *R*_r_ and *R*_s_, respectively (Fig 2B); the internal curve length of exocarp (*L*_e_), receptacle (*L*_r_), and curve length of seed space (*L*_s_), and the distance from rotation axis to the centroid of *L*_e_, *L*_r_ and *L*_s_, namely *r*_e_, *r*_r_, and *r*_s_, respectively (Fig 2C and S1 Table).

The volume *V*_*x*_ for each of exocarp (*V*_*e*_), receptacle (*V*_*r*_) and seed space (*V*_*s*_) was calculated by Eq 1; and the dimension *S*_*x*_ for each of the surface area of the seed space (*S*_*s*_), the coverage by exocarp (*S*_*e*_) and the coverage by receptacle (*S*_*r*_) was calculated by Eq 2. The total fruit size was represented by the fruit volume (*V*_*f*_), defined by *V*_f_ = *V*_s_ + *V*_e_ + *V*_r_.

$$V_{x}=A_{x}∙R_{x}$$ (1)

$$S_{x}=L_{x}∙r_{x}$$ (2)

### Morphometric analysis

All the data analysis was performed in R 3.3.1 (the R core team, 2016) [13]. Within 98 species sampled, there were 27 species with a single fruit (Table 1). For species with multiple fruit samples, the geometric mean of each dimension was used for species average. All 98 species were classified into two fruit types based on the species average seed surface coverage by exocarp and receptacle. If average seed surface coverage by exocarp was greater than that of receptacle, it was categorized as AC type; otherwise it was categorized as ER type (Table 1). We used Welch *t*-test for unequal variance to examine the distinction of the two fruit types from dimensions including the volume of three parts (exocarp, receptacle, and seed), the fruit size, and exocarp rotation angle (Table 2). Interspecific variation was examined across the 98 species (S1 Table), and intraspecific variation was examined on 4 ER-type species (each with the total of 7 or more fruits) and 7 AC-type species (each with the total of 16 or more fruits) (S2 Table). After logarithm transformation of all the morphological dimensions, we employed the standard major axis (MA) regression (package smatr 3.4.3 [14]) for among and within species allometry across selected pairs of dimensions. The within and among species collective properties were further examined by principal component analysis (PCA) on all dimensions except for the internal space surface area (*S*_*s*_), as *S*_*s*_ is proportional to seed space volume (*V*_*s*_), as well as to the sum of area enclosed by exocarp (*S*_*e*_) and receptacle (*S*_*r*_). We also performed H_ANGLE_ Fourier shape analysis [15] (S1 File) for cross-section images of fruit samples to compare the Fourier coefficients with the morphological dimensions, in terms of their effectiveness in capturing the morphological variations among species and between fruit types.

**Table 2:** The comparison of morphometric dimensions between the two fruit types.

Morphometric dimensions	AC	ER	d.f.	*P*
Fruit volume (cm^3^)	4.61	12.07	25.2	< 0.01
Seed volume (cm^3^)	2.42	4.66	24.9	< 0.05
Exocarp volume (cm^3^)	1.46	1.77	41.3	> 0.05
Receptacle volume (cm^3^)	0.74	5.64	21.9	< 0.05
Exocarp rotation angle (degree)	61.17	80.61	33.0	< 0.001

AC and ER represent acorn and enclosed receptacle fruit type respectively.

### Geospatial analysis

To study the variation of morphometric traits over geographic space, distribution range in latitude and elevation of 93 out of 98 examined species was retrieved from multiple sources including Flora of China (Vol.4; 1999), Flora Malesiana (Vol. 72; 1972), Flora of Japan (1984), and Global Biodiversity Information Facility (GBIF, www.gbif.org) (S5 Table). Each location name was translated to one pair of corresponding coordinates in decimal degrees by Google Earth. The latitudes in northern and southern hemisphere were translated to be positive and negative respectively, and the southernmost bond and northernmost bond were selected as the distribution range in latitude. We carried out generalized linear model (GLM) analysis (package lme4 1.1.13) [16] to examine the association between species geographic distribution with fruit type, fruit mechanical investment (fruit wall volume) and seed volume, in which fruit types were assumed to follow a binomial distribution, and log-transformed volumes were assumed to be normally distributed. We then selected parameters with respect to the Akaike Information Criterion (AIC). The geographic range of each species was represented by five points, i.e., (1) latitudinal midpoint and altitudinal midpoint, (2) latitudinal midpoint and altitudinal minimum, (3) latitudinal midpoint and altitudinal maximum, (4) altitudinal midpoint and latitudinal minimum, and (5) altitudinal midpoint and latitudinal maximum. The first point presents the distribution center, and the other four points are the distribution boundaries of four directions extended from the distribution center.

## Results

### Classification of fruit types

According to their definitions [4], 98 species were categorized into either fruit type through comparing the species mean seed enclosure by exocarp and receptacle tissue (Table 1). The problem of not enough variation for 27 species with single fruit sample (S1 Table) was redeemed by the significant seed coverage by either tissue (exocarp or receptacle), which made mis-categorization less likely. In total, there were 77 species with AC fruit type and 21 species with ER fruit type (Table 1). Fruits of 4 species exhibited heavy coverage by both exocarp and receptacle tissue (Table 1). They shared intermediate fruit morphology between AC and ER fruit with an elongated receptacle which almost covered the entire seed but the exocarp tissue was not reduced, sometimes even thickened (S3 Fig). Among them, three (*L*. *cryptocarpus*, *L*. *indutus*, and *L*. *konishii*) were categorized as AC type, and one (*L*. *lampadarius*) was categorized as ER type (Table 1).

### Morphometric differences between the two fruit types

**Fig 3:**
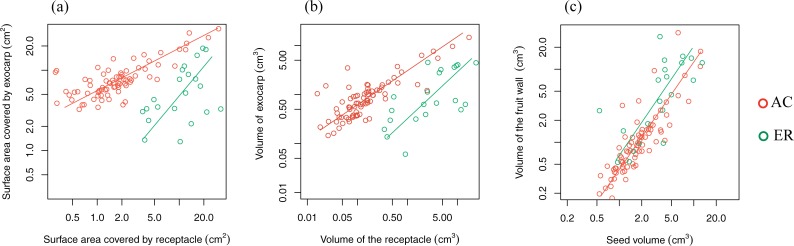
The interspecific variation of 98 species and between two fruit types. (a) The level of seed coverage by exocarp and receptacle. (b) The volume of exocarp and receptacle. (c) The fruit wall and seed volume. AC and ER stand for acorn and enclosed fruit type respectively. Each circle represents one species. The x and y axis of each graph are standardized.

Compared to AC species, the fruit and seed size of ER species were significantly larger (Table 2). The bigger exocarp rotation angle of ER fruits (Table 2) reflected a three-dimensional shape shift between the two fruit types: AC fruits tend to be semi-ellipsoid, while ER fruits are more spherical (Fig 1). The seeds of AC and ER fruits were enclosed and defended mainly by exocarp and receptacle tissue respectively (Fig 3A and 3B). Assuming zero density difference, the fruit wall volume (the total of exocarp and receptacle volume) represents the mechanical defense investment in biomass, so the ratio between fruit wall volume and seed surface area represents the relative mechanical protection. We found ER fruits were more physically protected than AC fruits (*P* < 0.01), which is further supported by MA analysis. With increasing in seed size, both fruit types increased their fruit wall volume accordingly (Fig 3C). But the higher elevation and bigger slope of ER species revealed a stronger physical defense investment compared to AC species. In terms of mechanical defense partitioning, there was no obvious difference in exocarp volume between the two fruit types, but there was a significantly higher investment in receptacle for ER fruits as mechanical defense (Table 2). From these results, we can see that the stronger physical defense of ER fruits is mainly contributed by receptacle tissue.

### The inter- and intraspecific variation of the two fruit types

The mechanical investment increased with seed size (Fig 3C). Despite the convergence of AC and ER fruit types with increasing fruit size, the two fruit types were well distinguished from each other by exocarp and receptacle dimensions (Fig 3A and 3B). For AC species, compared to two reconstructed dimensions of exocarp (seed coverage by exocarp and volume of exocarp), there was greater variation in two reconstructed dimensions of receptacle (with both allometric slope < 1), In contrast, for ER species, there was greater variation in the two dimensions of exocarp than those of receptacle (with both allometric slope >1).

**Fig 4:**
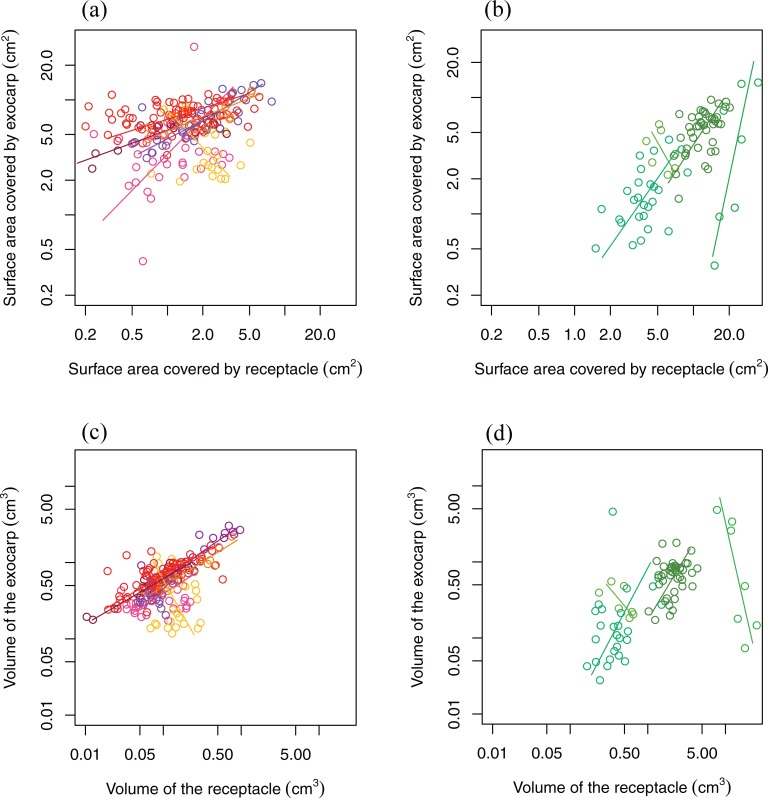
The intraspecific variation of the two fruit types. (a) The seed coverage by exocarp and receptacle of AC species. (b) The seed coverage by exocarp and receptacle of ER species. (c) The volume of exocarp and receptacle of AC species. (d) The volume of exocarp and receptacle of ER species. The scale of (a) and (b) are standardized as the scales in Fig 3 (A). The scale of (c) and (d) are standardized as the scales in Fig 3 (B). There are 7 AC species in (a) and (c), each species is represented by a specific color with the total of 16 or more fruits. There are 4 ER species in (b) and (d) each species is represented by a specific color with the total of 7 or more fruits. Each circle represents a single fruit.

The between-fruit-type variation was larger than within-fruit-type variation (Figs 3 and 4). It was observed that within AC species, *L*. *dealbatus* exhibited a ‘negative’ intraspecific allometric slope compared to the rest (Fig 4A and 4C). This could result from the distinct fruit morphology of this species: the spherical fruit with the elongated and curved receptacle tissue was somewhat similar to ER fruit morphology (S1 Fig). And the ‘negative’ slope of the two ER species (*L*. *cleistocarpus* and *L*. *javensis*) (Fig 4B and 4D) could have been caused by the small sample size, as both species were represented by 7 fruits only.

**Fig 5:**
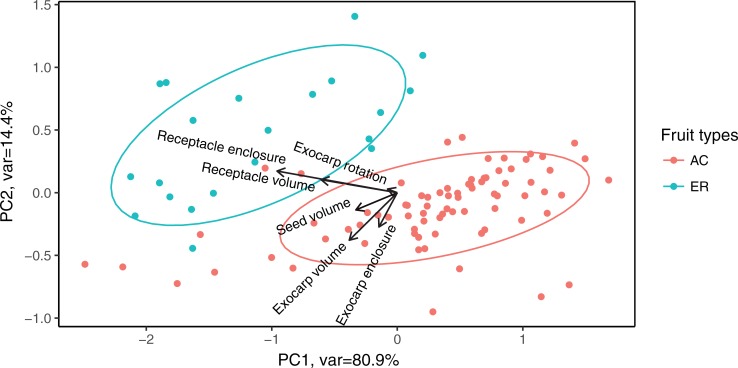
PCA of 98 species geometric mean of all dimensions of the two fruit types. AC and ER stand for acorn and enclosed fruit type respectively. Each dot represents a single species. The ellipses represent 68% confidence interval. The arrows represented the contributions from each variable.

**Fig 6:**
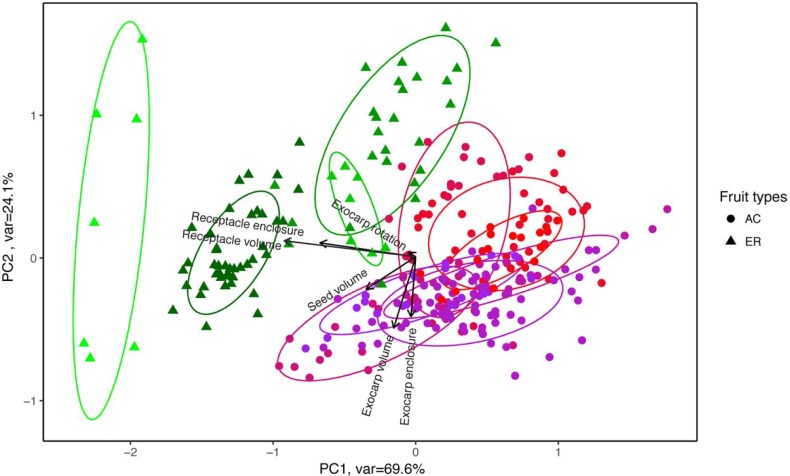
PCA of the morphometric dimensions on fruit individuals of 11 species. AC (7 species) and ER (4 species) stand for acorn and enclosed fruit type respectively. Each dot or triangle represents a single fruit. Each color represents a single species. The ellipses represent 68% confidence interval. The arrows represented the contributions from each variable.

From the PCA analysis of all 98 species (represented by 595 fruits in total), we found that the first two principal components captured 95.3% of the overall morphological distinction (Fig 5). The high degree of variation in the few AC species that did not cluster tightly with other AC species and even overlapped with some ER species is due to small sample sizes. Confining the analysis to only those species with more than 6 fruits greatly reduces the variation within AC-type species (the subset PCA figure not shown). PCA analysis for within species variation (Fig 6) showed a similar pattern that the first two axes accounted for 92.6% of the total variation. AC species formed a clear compact cluster, while each ER species was somewhat separated from each other, suggesting larger morphological variation among ER species. The additional PCA based on all data for inter- and intraspecific variation (S2 Fig) showed larger degree of divergence among species than within-species variation: the standard deviation of the scores of the first principle component among species was 0.99, meanwhile the maximum, median and minimum of intraspecific standard deviation of the first principle component were 0.56, 0.36 and 0.11 respectively. Despite the strong correlation between coverage and volume of both tissues (Figs 5 and 6), receptacle and exocarp represented the major morphometric differentiation between ER and AC types. Meanwhile, the PCA of the Fourier coefficients (S4 Fig) did not capture the morphological variance between the fruit types as effectively as the reconstructed morphological dimensions.

### Geospatial pattern of fruit morphological traits

**Fig 7:**
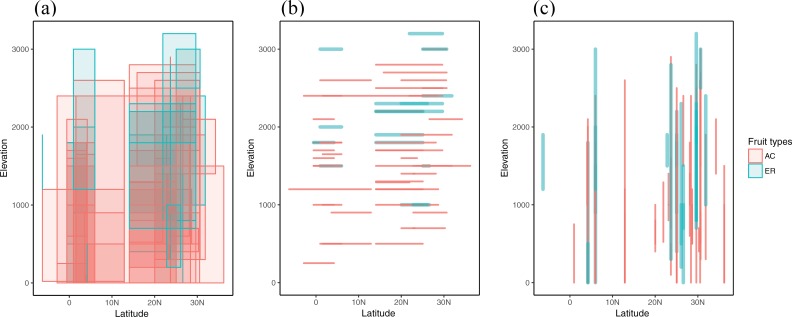
The geographic distribution of 93 *Lithocarpus* species. (a) Species distribution in altitudinal and latitudinal range. (b) Species distribution in latitudinal range with midpoint elevation. (c) Species distribution in altitudinal range with midpoint latitude. AC and ER stand for acorn and enclosed fruit type respectively.

Among 93 species with retrieved geostatistical information, each one was distributed in either region, except for *L*. *elegans*, which was documented by both Flora of China and Flora of Malesiana as a widely-distributed species in east Asia. The elevation for AC species ranged from sea level to 3000 m in southern China and from sea level to 2600 m in southeast Asia (Fig 7). For ER species, the altitudinal distribution ranged from 0 to over 3000 m in southern China, and 0 to 3000 m in southeast Asia. The latitudinal distributions for AC species ranged from 36N to 14N in southern China and 13N to 6S in southeast Asia, whereas those for ER species ranged from 32N to 14N in southern China, and 7N to 6S in southeast Asia. The great geographical overlap in the occurrence of AC and ER fruit types indicate that these two fruit types shared two common geographic distribution centers: southern China and southeast Asia. At higher elevation, ER-type species were more common than AC-type species (Table 3). Seed size increased with elevation but decreased with latitude. The fruit wall volume representing mechanical defense increased with both elevation and latitude (toward subtropical area). These suggested that species in bigger seed size and with better physical defense found on higher elevation are more likely to be ER fruits, which is consistent with the description of ER fruits based on the previous results (Table 2 and Fig 3). With increasing in latitude, the seed size decreases but the fruit physical defense increases.

**Table 3:** Dependence of fruit morphological dimensions on elevation and latitude.

Fruit traits	Constant	Elevation (km)	Latitude (degree)	Elevation by latitude interaction
Fruit type (AC ratio)	0.78	0.79		
Seed space volume (cm^3^)	2.59	0.17	–0.22 10^−2^	
Fruit wall volume (cm^3^)	1.17	0.72	0.64 10^−3^	–0.28 10^−1^

The appeared coefficients are significant based on model selection by Akaike’s Information Criterion (AIC).

## Discussion

The present study examined 595 individuals of 98 species, which represent 1/3 of the over 300 described *Lithocarpus* species. Our sample encompasses the fruit morphological diversity and the geographic distribution of species. We found that acorn (AC) and enclosed receptacle (ER) fruit types were the two dominant fruit types of stone oaks, with greater species richness in AC type (Table 1, 77 AC and 21 ER type). The greater species diversity of AC fruit type should not be a result of biased sampling, as our result was coincided with field observations and floristic literature surveys in China and southeast Asia [17–19], which suggested that AC-types species were more common than ER species at different locations with different forest types. Shape descriptors of exocarp and receptacle, also applied in the previous studies [4,8], were effective in capturing morphological variation within and between the fruit types (Figs 3 and 4). Combined with other morphological measurements, we found that morphometric difference among sampled fruits was considerably larger between two fruit types than that either within each fruit type or within each species (Figs 4, 5 and 6 and S2 Fig). This convergence on the same fruit type among a diversity of species indicates the presence of two fruit type ‘syndromes’ in the genus *Lithocarpus*, suggesting that the two fruit types are under sustained divergent selection. Combining with the previous fruit morphometric [4] and seed chemical study [4,8], the morphological distinction between two fruit types could be described as follows. With smaller seed and fruit size, semi-ellipsoid shaped AC fruits have their seeds enclosed mainly by exocarp tissue representing weak mechanical defense [4,8], and the seed antifeedants as chemical defense could be an important mechanism to reduce predation. Larger in seed and fruit size, ER fruits are spherical shaped and mainly enclosed by woody receptacle tissue representing strong mechanical defense with potentially higher nutrient content in the seeds [8]. And the less variability in exocarp and receptacle within fruit types implies the importance of these tissues in defining AC and ER fruits (Fig 3A and 3B). These distinctions between the two fruit types are stronger for species with smaller fruit size, while species with larger fruits exhibit an intermediate morphology that the seeds are heavily covered by both exocarp and receptacle tissues (Fig 3). This suggests that for large fruits, the mechanical protection by exocarp besides receptacle may be important and necessary for defending the seed. Four large-fruit species exhibit this intermediate fruit habit (Table 1 and S3 Fig). The phylogenetic relationship within *Lithocarpus* is under studied, and these species can be ideal materials for examining the phylogenetic relationship between two fruit types and the fruit evolution of *Lithocarpus*.

It turned out that compared to the H_ANGLE_ coefficients (S3 and S4 Tables), the seed-surface coverage and volume allocation among seed space, exocarp and receptacle were more efficient in capturing the fruit morphological variation (Figs 3 and 4). This could result from two possible reasons. First, the great number of species and fruits gave rise to big fruit morphological variation, which was hard to capture by Fourier analysis. Moreover, the outline smoothing in H_ANGLE_ could cause losing the fine-scale morphological variation of the fruit outlines.

Besides fruit morphology, the cupule of *Lithocarpus* fruits also exhibited great variation in texture, thickness and surface scale patterns [7]. Cupules vary from papery film to thickened texture, and from long tapering scales to reduced rings [20]. The main function of cupule is to protect seed during early fruit development [7], possibly against herbivory, but cupule is less likely to be the main physical protection of seed at maturity. Even though the embedment level of a mature fruit in the cupule varies greatly, the majority of species have the shallow cupule cover at the base of fruit, while only a relatively small number of species (mainly in the ER-type fruits) demonstrate cupule-enclosed fruits. Even in those cupule-enclosed fruits, cupules are often less woody compared to the fruit wall made of receptacle and exocarp, and often break easily into chunks during dissection. Therefore, even though cupule may be a partial reproductive energy investment, exocarp and receptacle provide major physical protection for a mature seed. Besides, a large amount of herbaria fruit samples lack of cupules, as the separation of the fruit from cupule during natural and artificial drying process. If morphological analysis of fruits including the cupule is to be carried out, we recommend using fresh fruit samples to avoid cupule fracture, and applying similar morphometric procedure for cupule/fruit sets that we employed for exocarp/receptacle/seed sets.

Previous studies on the altitudinal trends in seed size find different patterns of seed-size cline: the seed mass of pines increases with elevation among related species [21]; and the seed size of *Castanopsis* is larger in higher and lower altitudinal margins in Japan [22]. We found that the seed size of stone oaks increases with elevation (Table 3). This pattern could be explained as favorable conditions for seedling recruitment decreases with elevation [23], as factors such as short and cold growing season, soil instability could suppress seedling success. Large reserves per seed could compensate for various environmental stresses [21], which are essential for successful seedling establishment [6,24]. Similarly, the latitudinal trends in seed size also varied among taxa and studies: North American *Quercus* species with larger acorns are distributed in farther north [25]; while the larger-seeded *Castanopsis* species are found in the latitudinal margins in Japan [22]. Our study supports the general pattern revealed by most studies that larger seeds appear in tropics across taxa (Table 3) [26–28]. Several factors might contribute to the decrease in seed mass with latitude, such as the greater habitat shadiness, a greater variety of vertebrate dispersal agents, the highly reproductive climate, and larger stature of adult forms towards the equator [28]. Besides these factors, as one of the reproductive traits, seed size is more likely to be regulated by genetic differentiation rather than adaptive variation to local environments [22,29–34]. Overall, larger seeded *Lithocarpus* fruits with bigger per-seed resources in tropics and at higher elevation could indicate greater establishment success of offspring.

It is a widely accepted hypothesis that species at lower latitude have experienced more intensive predation pressure, and they have evolved to develop higher level of defense [35,36]. However, this hypothesis has been challenged by many recent large-scale meta analyses [26,37–40], as they all failed to support that predation is more intense towards the equator. Most of these studies focus on leaf defense against herbivory, with only a limited number of them [37] examining seed defense and predation. We found that the seeds of *Lithocarpus* are less physically protected at lower latitude (Table 3). One plausible explanation is the resource availability hypothesis [41,42], which suggests plants in resource-limited environments have higher defensive investment than those from more productive habitats [37]. Another possibility is that beside physical defense, chemical defense is alternative mechanism against predation [8]. Combining our results with the findings of recent studies [37,40], we challenge the traditional paradigm that higher predation pressure in the tropics and plants there will be better defended, and suggest this subject is mature for new theory and studies.

Even though insect and vertebrate predators have been found to decline with increasing elevation [43], different taxa, or biogeographic zones show different signs (positive versus negative) of the association between elevation and predation [44]. We found the seed physical defense increases with elevation (Table 3). Relating to the previous finding of larger seed at higher elevation, this is likely a strategy to reduce seed consumption. The species with bigger seeds and better mechanical protection at higher elevation coincide with the finding that ER-type species are common in higher elevation (Table 3).

Despite the more occurrence of ER at higher elevation, the two fruit types share two common distribution centers, southern China and southeast Asia (Fig 7, S5 Fig and Table 4). In addition, our field work and literatures [8,17–19] suggest that species with AC and ER fruits often co-occur and sometimes co-dominate in the same forest stand across geographic regions. One possibility is that the coexistence of the two fruit types could be a consequence of fruit-animal interactions (including antagonism and mutualism) [6]. Weevils, bark beetles, gall wasps, and crane flies are identified as herbivory pests [45], and granivore rodents of the genera *Niviventer*, *Rattus*, *Berylmys*, *Apodermus*, *Leopoldamys* and *Micromys* [46–48] and wild pigs (X Chen, pres. obs.) are scatter-hoarders of *Lithocarpus*. These vertebrate scatter-hoarders differ in their body size, gut characteristics, and their ability to cope with chemical and physical defenses [49]. The thickened and lignified husk of ER fruits representing strong physical protection could be a strategy for reducing pre-dispersal predation by insect pests [6], with evidence indicating the thicker pericarp is triggered by weevil infestation [50–54]. The scatter-hoarders with relatively large body size and strong jaws could better relocate and handle the large ER fruits [55]. In contrast, AC fruits with smaller seeds could attract a wider range of dispersal agents, but their weaker physical defense could be easy targets for insect predators. The antifeedants in the seeds could be an alternative defensive mechanism [8] as evidence suggests chemical defense reduces insect infestation [56].

**Table 4:** The geographical distribution of species with two fruit types.

Fruit types	Southern China region (> 15^o^N)	Southeast Asia region (< 15^o^N)
AC species number	45	27
ER species number	15	6

AC and ER stand for acorn and enclosed fruit type respectively. The AC type species, *L*. *elegans* is the only species documented to distribute in both regions of southern China and southeast Asia.

Comparative studies of species distribution along environmental gradients help one to uncover species interactions and fruit evolution [35,57,58]. The variation in fruit traits across the geospatial range of stone oaks shaped their diversity and the distribution pattern of the two main fruit types, which provides a background for future studies on the fruit evolution of *Lithocarpus* and the abiotic and biotic interactions over geographic gradients.

## Supporting information

S1 Fig The longitudinal section image of *L*. *dealbatus*. The fruit is enclosed within cupule (note the cupule here enclose the fruit at maturity is a rare case, which only happens in small number of species). The white line is the longitudinal axis.
(TIF)

S2 Fig PCA of interspecific and intraspecific geometric mean of all dimensions. The species appeared in both group was removed from the interspecific group to avoid duplication. Each dot and triangle represents one species.
(EPS)

S3 Fig Species with fruits covered by both exocarp and receptacle tissue. (TIF)

S4 Fig PCA by the exocarp and receptacle Fourier coefficients of the first 20 harmonics. (a) PCA of exocarp Fourier coefficients. (b) PCA of receptacle Fourier coefficients.
(TIF)

S5 Fig **The change in seed volume (a) and fruit wall size (b) of 93 *Lithocarpus* species with altitudinal and latitudinal midpoint.** The size of each point is proportional to the seed internal space volume in (a) and fruit husk volume in (b). AC and ER stand for acorn and enclosed fruit type respectively.
(EPS)

S1 File Description of H_ANGLE_ analysis. (DOCX)

S1 Table Exocarp rotation angle dimension and measured 12 morphometric parameters. (DOCX)

S2 Table Selected species for intraspecific variation analysis. (DOCX)

S3 Table The H_ANGLE_ coefficients of 2^nd^ to 10^th^ harmonics of exocarp. (DOCX)

S4 Table The H_ANGLE_ coefficients of 2^nd^ to 10^th^ harmonics of receptacle. (DOCX)

S5 Table The elevation and distribution information of 94 *Lithocarpus* species. (DOCX)
