# Correction: Associated morphometric and geospatial differentiation among 98 species of stone oaks (*Lithocarpus*)

**DOI:** 10.1371/journal.pone.0202461

**Published:** 2018-08-14

**Authors:** Xi Chen, Takashi S. Kohyama, Charles H. Cannon

In the Materials and Methods section, the first and second equations in the sub-section titled “Fruit morphological dimensions and parameters” are incorrect. Please view the complete, correct equations here:

V=2πAR

S=2πLr
