# Supplementary figures and images for: Associated morphometric and geospatial differentiation among 98 species of stone oaks (Lithocarpus)
Source: PLoS One. 2018 Jun 26;13(6):e0199538. doi: 10.1371/journal.pone.0199538 (PMC6019760; doi:10.1371/journal.pone.0199538)

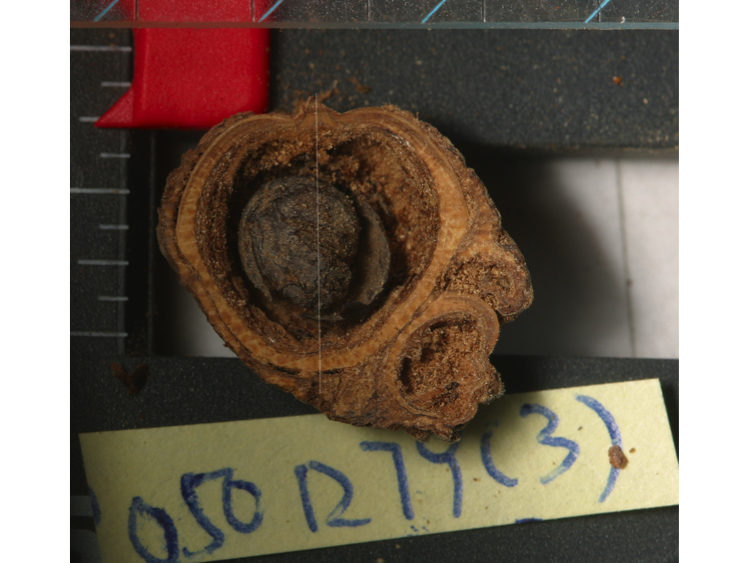

Supplement: S1 Fig — The fruit is enclosed within cupule (note the cupule here enclose the fruit at maturity is a rare case, which only happens in small number of species). The white line is the longitudinal axis. (TIF) [file pone.0199538.s001.tif]

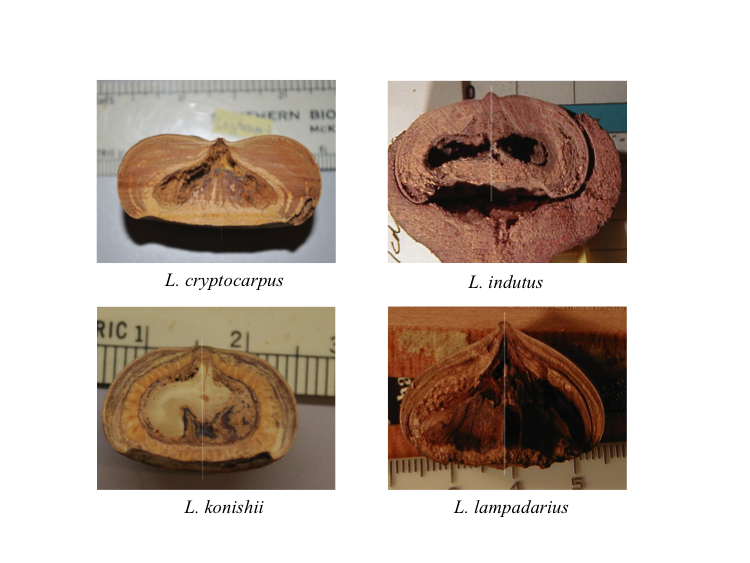

Supplement: S3 Fig — (TIF) [file pone.0199538.s003.tif]

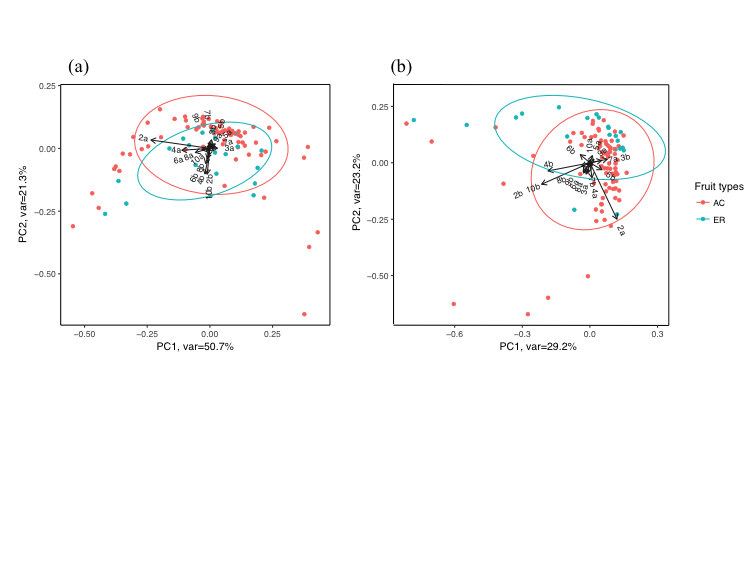

Supplement: S4 Fig — (a) PCA of exocarp Fourier coefficients. (b) PCA of receptacle Fourier coefficients. (TIF) [file pone.0199538.s004.tif]
