# Supplementary material for: Associated morphometric and geospatial differentiation among 98 species of stone oaks (Lithocarpus)
Source: PLoS One. 2018 Jun 26;13(6):e0199538. doi: 10.1371/journal.pone.0199538 (PMC6019760; doi:10.1371/journal.pone.0199538)
Supplement: S1 File — (DOCX) [file pone.0199538.s006.docx]

H_ANGLE_ analysis description: we applied an improved Fourier analysis program H_ANGLE_ to collect the coefficients of exocarp and receptacle outlines in the longitudinal section image (left-hand side). One fruit, sectioned and photographed, was selected to represent each species for all 98 stone oaks. The outlines of exocarp and receptacle for each species were traced manually in tps DIG 2.26 (F. James Rholf). The coordinate lists were exported to H_ANGLE_ to perform Fourier transformation with size standardization. As exocarp and receptacle tissue outlines are quite simple with less than 30 coordinates, 10 harmonics were retained for each outline. The first harmonic is usually very small and could be reconstructed by the rest of the harmonics, so only the second to the tenth harmonics (18 coefficients) were present for each tissue type (S3 and S4 Table) in the data analysis. PCA was applied to the original coefficients to evaluate the effectiveness of Fourier coefficients in discriminating AC and ER fruit types without standardization. As all the harmonics and coefficients generated from H_ANGLE_ were independent of each other, standardization would compromise the significance of the coefficients and introduce ‘noise’ to the result.
