# Supplementary material for: Associated morphometric and geospatial differentiation among 98 species of stone oaks (Lithocarpus)
Source: PLoS One. 2018 Jun 26;13(6):e0199538. doi: 10.1371/journal.pone.0199538 (PMC6019760; doi:10.1371/journal.pone.0199538)
Supplement: S1 Table — (DOCX) [file pone.0199538.s007.docx]

| Fruit type | Sample Source | Species | Specimennumber | Fruit  Individual | θ (degree) | A_e_ (cm^2^) | R_e_ (cm) | A_r_ (cm^2^) | R_r_ (cm) | A_s_ (cm^2^) | R_s_ (cm) | L_e_ (cm) | r_e_ (cm) | L_r_ (cm) | R_r_ (cm) | L_s_ (cm) | R_s_ (cm) |
| --- | --- | --- | --- | --- | --- | --- | --- | --- | --- | --- | --- | --- | --- | --- | --- | --- | --- |
| ER | Harvard | *L. amygdalifolius* | 10891 | Amy 1-1 | 61.50 | 0.33 | 0.78 | 0.62 | 0.68 | 1.28 | 0.39 | 2.12 | 0.87 | 2.26 | 0.80 | 3.00 | 0.65 |
| ER | Harvard | *L. amygdalifolius* | 10891 | Amy 1-2 | 63.50 | 0.27 | 0.64 | 0.33 | 0.63 | 1.30 | 0.41 | 1.86 | 0.80 | 2.05 | 0.72 | 2.99 | 0.56 |
| ER | Harvard | *L. amygdalifolius* | 10891 | Amy 1-3 | 67.50 | 0.21 | 0.64 | 0.67 | 0.69 | 1.16 | 0.42 | 1.46 | 0.57 | 1.96 | 0.59 | 2.66 | 0.52 |
| ER | Harvard | *L. amygdalifolius* | 10891 | Amy 1-4 | 67.50 | 0.25 | 0.70 | 0.79 | 0.80 | 1.59 | 0.47 | 1.67 | 0.59 | 2.97 | 0.73 | 3.35 | 0.84 |
| AC | Harvard | *L. bacgiangensis* | 11596 | Bac 1-1 | 76.00 | 0.39 | 0.81 | 0.09 | 0.53 | 0.96 | 0.45 | 2.24 | 0.76 | 1.28 | 0.57 | 2.71 | 0.57 |
| AC | Harvard | *L. bacgiangensis* | 78444 | Bac 2-1 | 85.00 | 0.31 | 0.75 | 0.13 | 0.54 | 0.65 | 0.40 | 1.87 | 0.62 | 1.39 | 0.79 | 2.33 | 0.60 |
| AC | KIB | *L. bacgiangensis* | 500884 | Bac 3-1 | 60.00 | 0.33 | 0.64 | 0.10 | 0.39 | 0.56 | 0.33 | 1.96 | 0.59 | 0.92 | 0.46 | 2.12 | 0.51 |
| ER | KIB | *L. balansae* | 500911 | Bal 1-1 | 67.50 | 0.12 | 0.42 | 0.44 | 0.53 | 1.20 | 0.32 | 1.26 | 0.42 | 3.03 | 0.46 | 3.03 | 0.58 |
| AC | Leiden | *L. bancanus* | B-102 | Bal 2-1 | 57.50 | 0.19 | 0.82 | 0.06 | 0.30 | 1.11 | 0.51 | 2.47 | 0.65 | 1.05 | 0.65 | 3.23 | 0.64 |
| AC | Leiden | *L. bancanus* | B-102 | Bal 3-1 | 48.96 | 0.18 | 0.82 | 0.06 | 0.23 | 1.42 | 0.52 | 2.76 | 0.66 | 0.90 | 0.42 | 3.37 | 0.71 |
| ER | Leiden | *L. beccarianus* | 99207 | Bec 1-1 | 81.50 | 0.14 | 0.86 | 1.67 | 0.87 | 1.94 | 0.34 | 1.46 | 0.85 | 3.75 | 0.67 | 4.14 | 0.60 |
| AC | Leiden | *L. bennettii* | Ashton5236 | Ben 1-1 | 52.50 | 0.06 | 0.42 | 0.02 | 0.16 | 0.44 | 0.27 | 1.62 | 0.41 | 0.41 | 0.22 | 1.94 | 0.43 |
| AC | Leiden | *L. bennettii* | IliasPaieS.15878 | Ben 2-1 | 50.00 | 0.06 | 0.53 | 0.02 | 0.26 | 0.50 | 0.34 | 1.62 | 0.51 | 0.62 | 0.27 | 2.18 | 0.39 |
| AC | Leiden | *L. bennettii* | JugahS.23688 | Ben 3-1 | 46.00 | 0.11 | 0.49 | 0.05 | 0.40 | 0.61 | 0.27 | 1.81 | 0.49 | 0.71 | 0.24 | 2.16 | 0.31 |
| AC | Leiden | *L. bennettii* | Wiriandinata3529 | Ben 4-1 | 51.00 | 0.06 | 0.41 | 0.02 | 0.21 | 0.32 | 0.26 | 1.30 | 0.43 | 0.56 | 0.27 | 1.82 | 0.37 |
| AC | Leiden | *L. bennettii* | Wood&WyattSmithA.4569 | Ben 5-1 | 57.50 | 0.07 | 0.46 | 0.03 | 0.26 | 0.31 | 0.32 | 1.44 | 0.40 | 0.68 | 0.25 | 2.02 | 0.49 |
| AC | Leiden | *L. blumeanus* | Arsat1150 | Blu 1-1 | 57.50 | 0.12 | 0.71 | 0.06 | 0.31 | 0.82 | 0.49 | 2.04 | 0.71 | 1.27 | 0.40 | 3.01 | 0.62 |
| AC | Leiden | *L. blumeanus* | Koorders33442 | Blu 2-1 | 63.00 | 0.34 | 0.69 | 0.05 | 0.30 | 0.66 | 0.39 | 1.98 | 0.64 | 0.64 | 0.29 | 2.48 | 0.47 |
| AC | Leiden | *L. blumeanus* | 34/1-35 | Blu 3-1 | 67.50 | 0.29 | 0.46 | 0.13 | 0.47 | 1.06 | 0.45 | 2.45 | 0.54 | 1.09 | 0.40 | 2.86 | 0.75 |
| AC | Leiden | *L. blumeanus* | Wood&Forest16199 | Blu 4-1 | 43.50 | 0.15 | 0.57 | 0.05 | 0.40 | 0.65 | 0.35 | 1.84 | 0.51 | 0.75 | 0.42 | 2.40 | 0.52 |
| AC | KIB | *L. brachystachyus* | 500926 | Bra 1-1 | 60.00 | 0.10 | 0.51 | 0.03 | 0.31 | 0.49 | 0.29 | 1.63 | 0.47 | 0.64 | 0.26 | 1.82 | 0.42 |
| AC | Harvard | *L. brevicaudatus* | 3637 | Bre 1-1 | 62.50 | 0.18 | 0.60 | 0.05 | 0.33 | 0.96 | 0.34 | 2.33 | 0.57 | 0.74 | 0.40 | 2.50 | 0.57 |
| AC | Field08 | *L. calolepis* | meibao2008-01 | Cal 1-1 | 87.50 | 0.28 | 0.96 | 0.21 | 0.63 | 1.53 | 0.52 | 2.50 | 0.82 | 1.59 | 0.64 | 3.26 | 0.77 |
| AC | Field08 | *L. calolepis* | meibao2008-02 | Cal 1-2 | 91.50 | 0.31 | 0.96 | 0.20 | 0.50 | 1.62 | 0.51 | 2.55 | 0.83 | 1.29 | 0.51 | 3.29 | 0.58 |
| AC | Field08 | *L. calolepis* | meibao2008-03 | Cal 1-3 | 82.50 | 0.28 | 0.93 | 0.18 | 0.59 | 1.47 | 0.51 | 2.42 | 0.81 | 1.39 | 0.74 | 3.20 | 0.73 |
| AC | Field08 | *L. calolepis* | meibao2008-04 | Cal 1-4 | 80.00 | 0.27 | 0.94 | 0.17 | 0.57 | 1.55 | 0.55 | 2.52 | 0.92 | 1.50 | 0.73 | 3.31 | 0.73 |
| AC | Field08 | *L. calolepis* | 024HD | Cal 1-5 | 92.50 | 0.39 | 0.74 | 0.16 | 0.50 | 0.95 | 0.39 | 1.84 | 0.61 | 1.38 | 0.51 | 2.61 | 0.49 |
| AC | KIB | *L. calophyllus* | 804301 | Clp 1-1 | 54.00 | 0.29 | 0.61 | 0.06 | 0.38 | 0.88 | 0.33 | 2.28 | 0.45 | 0.80 | 0.45 | 2.43 | 0.45 |
| AC | KIB | *L. carolinae* | 500952 | Car 1-1 | 91.00 | 0.40 | 0.89 | 0.15 | 0.59 | 1.03 | 0.55 | 2.24 | 0.93 | 1.17 | 0.58 | 3.19 | 0.69 |
| AC | KIB | *L. caudatilimbus* | 500951 | Cau 1-1 | 77.50 | 0.28 | 0.81 | 0.05 | 0.23 | 1.33 | 0.43 | 2.44 | 0.71 | 0.57 | 0.28 | 2.91 | 0.65 |
| AC | Leiden | *L. celebicus* | A.G.Waturaudaug25.1934 | Cel 1-1 | 50.00 | 0.27 | 0.69 | 0.17 | 0.31 | 1.00 | 0.41 | 2.45 | 0.69 | 1.11 | 0.29 | 3.18 | 0.63 |
| AC | Leiden | *L. celebicus* | H.N.Reppie159.1934 | Cel 2-1 | 62.50 | 0.24 | 0.58 | 0.04 | 0.17 | 0.80 | 0.33 | 2.04 | 0.52 | 0.55 | 0.09 | 2.43 | 0.41 |
| AC | Leiden | *L. celebicus* | H.N.Reppie159.1934 | Cel 3-1 | 56.56 | 0.19 | 0.51 | 0.04 | 0.18 | 0.74 | 0.34 | 2.07 | 0.49 | 0.51 | 0.04 | 2.45 | 0.49 |
| AC | Leiden | *L. celebicus* | Koorders16622 | Cel 4-1 | 47.50 | 0.32 | 0.58 | 0.07 | 0.19 | 0.85 | 0.33 | 2.24 | 0.54 | 0.63 | 0.31 | 2.73 | 0.51 |
| AC | Leiden | *L. celebicus* | Koorders16622 | Cel 5-1 | 42.51 | 0.25 | 0.54 | 0.05 | 0.28 | 0.67 | 0.35 | 2.14 | 0.57 | 0.66 | 0.45 | 2.56 | 0.49 |
| AC | KIB | *L. chrysocomus* | 500958 | Cry 1-1 | 70.00 | 0.10 | 0.49 | 0.07 | 0.37 | 0.58 | 0.31 | 1.23 | 0.52 | 0.83 | 0.42 | 2.01 | 0.46 |
| ER | Harvard | *L. cleistocarpus* | 728 | Cle 1-1 | 85.00 | 0.08 | 0.49 | 0.13 | 0.53 | 0.84 | 0.34 | 1.05 | 0.49 | 1.79 | 0.63 | 2.35 | 0.59 |
| ER | Harvard | *L. cleistocarpus* | 728 | Cle 1-2 | 85.00 | 0.07 | 0.49 | 0.19 | 0.56 | 0.84 | 0.33 | 0.99 | 0.40 | 2.21 | 0.50 | 2.39 | 0.49 |
| ER | Harvard | *L. cleistocarpus* | 728 | Cle 1-3 | 87.50 | 0.07 | 0.42 | 0.16 | 0.57 | 0.79 | 0.32 | 0.90 | 0.38 | 1.79 | 0.54 | 2.29 | 0.50 |
| ER | Harvard | *L. cleistocarpus* | 4009 | Cle 2-1 | 82.50 | 0.09 | 0.40 | 0.20 | 0.51 | 0.63 | 0.26 | 1.12 | 0.39 | 1.42 | 0.51 | 2.07 | 0.39 |
| ER | KIB | *L. cleistocarpus* | 500987 | Cle 3-1 | 67.50 | 0.16 | 0.57 | 0.11 | 0.47 | 1.03 | 0.30 | 1.64 | 0.41 | 1.50 | 0.43 | 2.63 | 0.51 |
| ER | KIB | *L. cleistocarpus* | 500971 | Cle 3-2 | 85.00 | 0.11 | 0.57 | 0.08 | 0.47 | 0.79 | 0.33 | 1.55 | 0.60 | 1.50 | 0.55 | 2.30 | 0.44 |
| ER | KIB | *L. cleistocarpus* | 501036 | Cle 4-1 | 87.50 | 0.14 | 0.57 | 0.16 | 0.49 | 0.77 | 0.28 | 1.54 | 0.54 | 1.55 | 0.55 | 2.24 | 0.50 |
| AC | Leiden | *L. confertus* | J.&M.S.Clemens29265 | Con 1-1 | 55.00 | 0.09 | 0.50 | 0.04 | 0.33 | 0.51 | 0.34 | 1.61 | 0.54 | 0.68 | 0.25 | 2.22 | 0.44 |
| AC | Leiden | *L. confertus* | J.&M.S.Clemens29265 | Con 1-2 | 50.26 | 0.12 | 0.59 | 0.04 | 0.28 | 0.62 | 0.38 | 1.82 | 0.58 | 0.67 | 0.33 | 2.37 | 0.53 |
| AC | Leiden | *L. confertus* | J.&M.S.Clemens29265 | Con 1-3 | 47.08 | 0.10 | 0.49 | 0.03 | 0.20 | 0.49 | 0.34 | 1.84 | 0.54 | 0.49 | 0.34 | 2.19 | 0.59 |
| AC | Harvard | *L. confinis* | 34945 | Cnf 1-1 | 50.00 | 0.10 | 0.47 | 0.05 | 0.38 | 0.57 | 0.29 | 1.81 | 0.48 | 0.92 | 0.44 | 2.10 | 0.47 |
| AC | KIB | *L. confinis* | 501068 | Cnf 2-1 | 51.50 | 0.14 | 0.55 | 0.05 | 0.27 | 0.73 | 0.32 | 2.03 | 0.59 | 0.58 | 0.23 | 2.30 | 0.49 |
| AC | KIB | *L. confinis* | 501068 | Cnf 2-2 | 52.00 | 0.13 | 0.50 | 0.05 | 0.31 | 0.70 | 0.32 | 1.95 | 0.49 | 0.73 | 0.37 | 2.22 | 0.46 |
| AC | KIB | *L. confinis* | 501068 | Cnf 2-3 | 47.50 | 0.14 | 0.48 | 0.03 | 0.26 | 0.62 | 0.27 | 1.89 | 0.50 | 0.55 | 0.23 | 2.10 | 0.41 |
| AC | KIB | *L. confinis* | 501068 | Cnf 2-4 | 52.50 | 0.11 | 0.52 | 0.06 | 0.29 | 0.62 | 0.28 | 1.73 | 0.48 | 0.61 | 0.31 | 2.02 | 0.35 |
| AC | KIB | *L. confinis* | 501059 | Cnf 3-1 | 50.00 | 0.20 | 0.49 | 0.09 | 0.34 | 0.55 | 0.27 | 1.86 | 0.48 | 0.70 | 0.28 | 1.94 | 0.46 |
| AC | KIB | *L. confinis* | 501059 | Cnf 3-2 | 50.00 | 0.21 | 0.48 | 0.05 | 0.28 | 0.58 | 0.25 | 1.88 | 0.40 | 0.59 | 0.32 | 1.98 | 0.36 |
| AC | Leiden | *L. conocarpus* | H.Hallier2518 | Cno 1-1 | 51.50 | 0.32 | 0.76 | 0.06 | 0.41 | 1.09 | 0.42 | 2.47 | 0.65 | 0.74 | 0.29 | 2.98 | 0.62 |
| ER | Harvard | *L. corneus* | 24803 | Cor 1-1 | 95.00 | 0.94 | 1.38 | 2.16 | 0.91 | 1.26 | 0.48 | 2.01 | 1.12 | 3.36 | 0.50 | 3.37 | 0.73 |
| ER | Harvard | *L. corneus* | 24803 | Cor 1-2 | 87.50 | 0.87 | 0.41 | 2.39 | 0.87 | 3.25 | 0.54 | 1.98 | 0.99 | 3.78 | 0.56 | 3.76 | 0.70 |
| ER | Harvard | *L. corneus* | 24159 | Cor 2-1 | 100.00 | 0.42 | 1.14 | 2.00 | 0.77 | 0.80 | 0.34 | 1.61 | 0.91 | 2.55 | 0.45 | 2.53 | 0.48 |
| ER | Harvard | *L. corneus* | 8523 | Cor 3-1 | 89.00 | 0.89 | 1.25 | 1.61 | 0.93 | 1.85 | 0.54 | 2.27 | 1.05 | 3.65 | 0.81 | 3.66 | 0.92 |
| ER | KIB | *L. corneus* | 501163 | Cor 4-1 | 87.50 | 0.35 | 1.03 | 1.21 | 0.68 | 0.84 | 0.46 | 1.58 | 0.76 | 2.97 | 0.59 | 3.01 | 0.53 |
| AC | Field08 | *L. craibianus* | cra2008-in5 | Cra 1-1 | 65.00 | 0.11 | 0.48 | 0.04 | 0.22 | 0.44 | 0.26 | 1.46 | 0.46 | 0.49 | 0.22 | 1.72 | 0.38 |
| AC | Field08 | *L. craibianus* | cra2008-in5 | Cra 1-2 | 70.00 | 0.09 | 0.41 | 0.03 | 0.19 | 0.33 | 0.22 | 1.20 | 0.35 | 0.43 | 0.20 | 1.50 | 0.35 |
| AC | Field08 | *L. craibianus* | cra2008-in5 | Cra 1-3 | 67.50 | 0.11 | 0.42 | 0.03 | 0.18 | 0.36 | 0.22 | 1.28 | 0.22 | 0.42 | 0.18 | 1.56 | 0.33 |
| AC | Field08 | *L. craibianus* | cra2008-in5 | Cra 1-4 | 69.00 | 0.09 | 0.44 | 0.03 | 0.21 | 0.34 | 0.23 | 1.21 | 0.36 | 0.50 | 0.32 | 1.56 | 0.34 |
| AC | Field08 | *L. craibianus* | cra2008-in5 | Cra 1-5 | 70.00 | 0.09 | 0.48 | 0.04 | 0.20 | 0.41 | 0.26 | 1.30 | 0.36 | 0.48 | 0.25 | 1.67 | 0.36 |
| AC | Field08 | *L. craibianus* | cra2008-in5 | Cra 1-6 | 70.00 | 0.13 | 0.53 | 0.05 | 0.26 | 0.45 | 0.30 | 1.47 | 0.53 | 0.58 | 0.25 | 1.74 | 0.37 |
| AC | Field08 | *L. craibianus* | cra2008-in5 | Cra 1-7 | 65.00 | 0.11 | 0.44 | 0.04 | 0.20 | 0.42 | 0.25 | 0.14 | 0.44 | 0.49 | 0.20 | 1.68 | 0.37 |
| AC | Field08 | *L. craibianus* | cra2008-in5 | Cra 1-8 | 65.00 | 0.13 | 0.51 | 0.06 | 0.25 | 0.40 | 0.28 | 1.35 | 0.49 | 0.58 | 0.14 | 1.71 | 0.43 |
| AC | Field08 | *L. craibianus* | cra2008-in5 | Cra 1-9 | 75.00 | 0.16 | 0.63 | 0.05 | 0.29 | 0.69 | 0.35 | 1.69 | 0.60 | 0.60 | 0.27 | 2.19 | 0.53 |
| AC | KIB | *L. craibianus* | 501190 | Cra 2-1 | 65.00 | 0.10 | 0.49 | 0.04 | 0.29 | 0.48 | 0.24 | 1.29 | 0.38 | 0.72 | 0.21 | 1.79 | 0.34 |
| AC | KIB | *L. craibianus* | 501241 | Cra 3-1 | 70.00 | 0.10 | 0.51 | 0.06 | 0.30 | 0.67 | 0.27 | 1.37 | 0.44 | 0.73 | 0.31 | 2.07 | 0.45 |
| AC | KIB | *L. craibianus* | 501243 | Cra 3-1 | 70.00 | 0.09 | 0.58 | 0.07 | 0.41 | 0.73 | 0.33 | 1.40 | 0.38 | 1.13 | 0.42 | 2.21 | 0.45 |
| AC | Field08 | *L. craibianus* | cra2008-in1 | Cra 4-1 | 70.00 | 0.14 | 0.60 | 0.05 | 0.28 | 0.59 | 0.34 | 1.61 | 0.63 | 0.59 | 0.32 | 2.03 | 0.51 |
| AC | KIB | *L. craibianus* | 468793 | Cra 5-1 | 65.00 | 0.08 | 0.41 | 0.04 | 0.22 | 0.39 | 0.21 | 1.20 | 0.36 | 0.64 | 0.34 | 1.58 | 0.27 |
| AC | KIB | *L. craibianus* | 468793 | Cra 5-2 | 62.50 | 0.09 | 0.41 | 0.03 | 0.23 | 0.40 | 0.21 | 1.21 | 0.37 | 0.65 | 0.33 | 1.60 | 0.12 |
| AC | KIB | *L. craibianus* | 468793 | Cra 5-3 | 60.00 | 0.09 | 0.40 | 0.03 | 0.21 | 0.42 | 0.20 | 1.26 | 0.20 | 0.52 | 0.21 | 1.64 | 0.36 |
| AC | Field08 | *L. craibianus* | cra2008-in1 | Cra 4-2 | 72.50 | 0.15 | 0.61 | 0.07 | 0.36 | 0.65 | 0.33 | 1.68 | 0.63 | 0.87 | 0.32 | 2.10 | 0.51 |
| AC | Field08 | *L. craibianus* | cra2008-in1 | Cra 4-3 | 75.00 | 0.15 | 0.59 | 0.05 | 0.30 | 0.62 | 0.32 | 1.58 | 0.59 | 0.77 | 0.22 | 2.04 | 0.41 |
| AC | Field08 | *L. craibianus* | cra2008-in6 | Cra 6-1 | 67.50 | 0.12 | 0.53 | 0.05 | 0.22 | 0.51 | 0.30 | 1.58 | 0.49 | 0.48 | 0.24 | 1.90 | 0.49 |
| AC | Field08 | *L. craibianus* | cra2008-in6 | Cra 6-2 | 62.50 | 0.11 | 0.43 | 0.04 | 0.17 | 0.40 | 0.24 | 1.42 | 0.21 | 0.42 | 0.20 | 1.69 | 0.40 |
| AC | Field08 | *L. craibianus* | cra2008-in6 | Cra 6-3 | 70.00 | 0.12 | 0.48 | 0.05 | 0.22 | 0.46 | 0.28 | 1.51 | 0.46 | 0.51 | 0.31 | 1.81 | 0.38 |
| AC | Field08 | *L. craibianus* | cra2008-in2 | Cra 7-1 | 70.00 | 0.13 | 0.52 | 0.06 | 0.25 | 0.48 | 0.28 | 1.55 | 0.50 | 0.65 | 0.42 | 1.84 | 0.47 |
| AC | Field08 | *L. craibianus* | cra2008-in2 | Cra 7-2 | 72.50 | 0.15 | 0.52 | 0.05 | 0.20 | 0.52 | 0.28 | 1.63 | 0.47 | 0.50 | 0.24 | 1.90 | 0.38 |
| AC | Field08 | *L. craibianus* | cra2008-in2 | Cra 7-3 | 70.00 | 0.13 | 0.52 | 0.06 | 0.24 | 0.48 | 0.30 | 1.56 | 0.55 | 0.58 | 0.23 | 1.88 | 0.43 |
| AC | Field08 | *L. craibianus* | cra2008-in3 | Cra 8-1 | 67.50 | 0.14 | 0.50 | 0.03 | 0.17 | 0.55 | 0.27 | 1.72 | 0.47 | 0.36 | 0.10 | 1.89 | 0.44 |
| AC | Field08 | *L. craibianus* | cra2008-in3 | Cra 8-2 | 70.00 | 0.13 | 0.56 | 0.04 | 0.26 | 0.60 | 0.31 | 1.66 | 0.48 | 0.58 | 0.26 | 1.99 | 0.36 |
| AC | Field08 | *L. craibianus* | cra2008-in3 | Cra 8-3 | 67.50 | 0.14 | 0.57 | 0.04 | 0.23 | 0.61 | 0.31 | 1.69 | 0.43 | 0.50 | 0.12 | 2.04 | 0.49 |
| AC | Field08 | *L. craibianus* | 017HD | Cra 9-1 | 80.00 | 0.06 | 0.44 | 0.05 | 0.34 | 0.30 | 0.24 | 0.90 | 0.38 | 0.78 | 0.17 | 1.48 | 0.37 |
| AC | Field08 | *L. craibianus* | 017HD | Cra 9-2 | 60.00 | 0.07 | 0.42 | 0.04 | 0.24 | 0.37 | 0.22 | 1.10 | 0.20 | 0.54 | 0.22 | 1.56 | 0.30 |
| AC | Field08 | *L. craibianus* | 007HD | Cra 9-3 | 72.50 | 0.08 | 0.48 | 0.08 | 0.37 | 0.51 | 0.27 | 1.07 | 0.37 | 0.89 | 0.34 | 1.88 | 0.42 |
| AC | Field08 | *L. craibianus* | 007HD | Cra 9-4 | 75.00 | 0.13 | 0.56 | 0.07 | 0.36 | 0.61 | 0.29 | 1.33 | 3.46 | 0.79 | 0.34 | 2.00 | 0.47 |
| AC | Field08 | *L. craibianus* | 007HD | Cra 9-5 | 70.00 | 0.08 | 0.53 | 0.04 | 0.20 | 0.45 | 0.28 | 1.28 | 0.42 | 0.61 | 0.15 | 1.78 | 0.37 |
| AC | Field08 | *L. craibianus* | 003HD | Cra 10-1 | 60.00 | 0.08 | 0.57 | 0.08 | 0.41 | 0.61 | 0.30 | 1.18 | 0.48 | 1.03 | 0.39 | 2.03 | 0.41 |
| AC | Field08 | *L. craibianus* | 003HD | Cra 10-2 | 65.00 | 0.05 | 3.13 | 0.07 | 0.39 | 0.45 | 0.25 | 0.92 | 0.37 | 1.13 | 0.20 | 1.72 | 0.44 |
| AC | Field08 | *L. craibianus* | 003HD | Cra 10-3 | 62.50 | 0.08 | 0.58 | 0.09 | 0.40 | 0.57 | 0.35 | 1.11 | 0.42 | 1.13 | 0.35 | 2.08 | 0.47 |
| AC | Field08 | *L. craibianus* | 002HD | Cra 11-1 | 65.00 | 0.09 | 0.55 | 0.07 | 0.40 | 0.71 | 0.30 | 1.30 | 0.39 | 1.24 | 0.43 | 2.15 | 0.49 |
| AC | Field08 | *L. craibianus* | 002HD | Cra 11-2 | 62.50 | 0.07 | 0.56 | 0.06 | 0.43 | 0.65 | 0.31 | 0.93 | 0.47 | 1.05 | 0.44 | 2.09 | 0.48 |
| AC | Field08 | *L. craibianus* | 002HD | Cra 11-3 | 60.00 | 0.08 | 0.51 | 0.06 | 0.38 | 0.55 | 0.28 | 1.18 | 0.48 | 0.83 | 0.36 | 1.92 | 0.47 |
| AC | Leiden | *L. crassinervius* | Koorders39442 | Crs 1-1 | 50.00 | 0.58 | 0.81 | 0.02 | 0.23 | 1.93 | 0.43 | 3.52 | 0.65 | 0.75 | 0.26 | 3.79 | 0.59 |
| AC | Leiden | *L. crassinervius* | Koorders39442 | Crs 1-2 | 47.50 | 0.79 | 0.83 | 0.06 | 0.36 | 1.51 | 0.37 | 3.16 | 0.68 | 0.82 | 0.39 | 3.25 | 0.55 |
| ER | Harvard | *L. cryptocarpus* | 8345 | Cry 1-1 | 92.50 | 1.26 | 1.84 | 2.53 | 1.07 | 1.16 | 0.85 | 3.88 | 1.35 | 4.22 | 1.14 | 4.55 | 0.95 |
| ER | Leiden | *L. cyclophorus* | Boschproefstation7429 | Cyc 1-1 | 97.50 | 1.68 | 2.07 | 3.25 | 1.65 | 2.87 | 0.94 | 3.73 | 1.54 | 5.38 | 1.13 | 6.22 | 1.17 |
| ER | Leiden | *L. cyclophorus* | vanSteenis3597 | Cyc 2-1 | 95.00 | 0.69 | 1.57 | 0.87 | 0.86 | 1.79 | 0.77 | 2.61 | 1.27 | 2.91 | 0.76 | 4.62 | 1.10 |
| ER | Leiden | *L. cyclophorus* | vanSteenis3597 | Cyc 2-2 | 90.00 | 0.67 | 1.41 | 0.59 | 0.88 | 2.33 | 0.87 | 2.93 | 1.18 | 3.22 | 1.09 | 4.96 | 0.88 |
| ER | KIB | *L. damiaoshanicus* | 501297 | Dam 1-1 | 77.50 | 0.07 | 0.44 | 0.11 | 0.51 | 0.74 | 0.29 | 0.92 | 0.41 | 1.47 | 0.46 | 2.18 | 0.38 |
| AC | Leiden | *L. dasystachyus* | Abbe,Smythie&Asah9825 | Das 1-1 | 45.00 | 0.13 | 0.43 | 0.03 | 0.19 | 0.53 | 0.24 | 1.83 | 0.42 | 0.46 | 0.20 | 1.98 | 0.40 |
| AC | Leiden | *L. dasystachyus* | Pasqual2373(207) | Das 2-1 | 47.50 | 0.07 | 0.37 | 0.02 | 0.15 | 0.42 | 0.19 | 1.57 | 0.30 | 0.34 | 0.16 | 1.74 | 0.30 |
| AC | Leiden | *L. dasystachyus* | RidsdalePBU.470 | Das 3-1 | 45.00 | 0.08 | 0.37 | 0.02 | 0.10 | 0.51 | 0.21 | 1.80 | 0.36 | 0.24 | 0.12 | 1.90 | 0.35 |
| AC | Harvard | *L. dealbatus* | 3630 | Dea 1-1 | 52.50 | 0.23 | 0.66 | 0.09 | 0.31 | 0.95 | 0.38 | 2.31 | 0.60 | 0.71 | 0.32 | 2.72 | 0.55 |
| AC | Harvard | *L. dealbatus* | 3630 | Dea 1-2 | 56.00 | 0.26 | 0.69 | 0.07 | 0.28 | 0.90 | 0.35 | 2.26 | 0.58 | 0.65 | 0.23 | 2.53 | 0.18 |
| AC | Harvard | *L. dealbatus* | 6505 | Dea 2-1 | 65.00 | 0.31 | 0.61 | 0.04 | 0.30 | 0.87 | 0.33 | 2.24 | 0.47 | 0.69 | 0.29 | 2.41 | 0.48 |
| AC | Harvard | *L. dealbatus* | 6505 | Dea 2-2 | 62.50 | 0.24 | 0.57 | 0.07 | 0.36 | 0.77 | 0.32 | 2.18 | 0.58 | 0.79 | 0.30 | 2.31 | 0.47 |
| AC | Harvard | *L. dealbatus* | 6505 | Dea 2-3 | 65.00 | 0.27 | 0.58 | 0.04 | 0.32 | 0.73 | 0.31 | 2.06 | 0.50 | 0.65 | 0.33 | 2.20 | 0.41 |
| AC | Harvard | *L. dealbatus* | 3106 | Dea 3-1 | 70.00 | 0.08 | 0.47 | 0.06 | 0.38 | 0.40 | 0.23 | 1.04 | 0.38 | 1.10 | 0.39 | 1.64 | 0.37 |
| AC | Harvard | *L. dealbatus* | 3106 | Dea 3-2 | 75.00 | 0.07 | 0.49 | 0.06 | 0.38 | 0.46 | 0.28 | 1.15 | 0.46 | 0.98 | 0.44 | 1.75 | 0.40 |
| AC | Harvard | *L. dealbatus* | 3106 | Dea 3-3 | 65.00 | 0.06 | 0.40 | 0.06 | 0.37 | 0.39 | 0.23 | 0.91 | 0.38 | 1.00 | 0.39 | 1.60 | 0.38 |
| AC | Harvard | *L. dealbatus* | 62--4 | Dea 4-1 | 70.00 | 0.06 | 0.48 | 0.08 | 0.33 | 0.53 | 0.27 | 1.11 | 0.39 | 0.92 | 0.39 | 1.96 | 0.41 |
| AC | Harvard | *L. dealbatus* | 62--5 | Dea 5-1 | 57.50 | 0.06 | 0.45 | 0.09 | 0.34 | 0.45 | 0.25 | 1.08 | 0.53 | 0.97 | 0.36 | 1.75 | 0.34 |
| AC | KIB | *L. dealbatus* | 671534 | Dea 6-1 | 67.50 | 0.06 | 0.43 | 0.06 | 0.33 | 0.37 | 0.13 | 1.04 | 0.39 | 1.12 | 0.34 | 1.61 | 0.24 |
| AC | KIB | *L. dealbatus* | 671534 | Dea 6-2 | 65.00 | 0.06 | 0.38 | 0.04 | 0.27 | 0.31 | 0.20 | 0.94 | 0.33 | 0.74 | 0.27 | 1.41 | 0.30 |
| AC | KIB | *L. dealbatus* | 671568 | Dea 7-1 | 62.50 | 0.06 | 0.42 | 0.04 | 0.30 | 0.40 | 0.22 | 1.11 | 0.41 | 0.80 | 0.41 | 1.63 | 0.29 |
| AC | KIB | *L. dealbatus* | 671568 | Dea 7-2 | 60.00 | 0.06 | 0.41 | 0.04 | 0.31 | 0.40 | 0.22 | 1.03 | 0.39 | 0.78 | 0.36 | 1.61 | 0.31 |
| AC | KIB | *L. dealbatus* | 671568 | Dea 7-3 | 61.50 | 0.05 | 0.42 | 0.06 | 0.31 | 0.35 | 0.24 | 0.99 | 0.37 | 0.77 | 0.15 | 1.51 | 0.38 |
| AC | KIB | *L. dealbatus* | 501279 | Dea 8-1 | 67.50 | 0.05 | 0.47 | 0.12 | 0.37 | 0.33 | 0.23 | 0.83 | 0.39 | 1.33 | 0.37 | 1.51 | 0.33 |
| AC | KIB | *L. dealbatus* | 501279 | Dea 8-2 | 70.00 | 0.06 | 0.45 | 0.11 | 0.44 | 0.47 | 0.28 | 0.96 | 0.45 | 1.45 | 0.46 | 1.80 | 0.44 |
| AC | KIB | *L. dealbatus* | 501279 | Dea 8-3 | 58.50 | 0.05 | 0.38 | 0.06 | 0.33 | 0.40 | 0.22 | 0.94 | 0.35 | 1.16 | 0.39 | 1.62 | 0.34 |
| AC | Harvard | *L. dealbatus* | 7747 | Dea 9-1 | 65.00 | 0.13 | 0.54 | 0.05 | 0.36 | 0.74 | 0.30 | 1.54 | 0.53 | 0.94 | 0.35 | 2.18 | 0.58 |
| AC | Harvard | *L. dealbatus* | 7747 | Dea 9-2 | 62.50 | 0.12 | 0.56 | 0.10 | 0.43 | 0.84 | 0.32 | 1.52 | 0.58 | 1.24 | 0.31 | 2.31 | 0.48 |
| AC | Harvard | *L. dealbatus* | 7747 | Dea 9-3 | 70.00 | 0.13 | 0.59 | 0.08 | 0.40 | 0.81 | 0.31 | 1.51 | 0.56 | 1.12 | 0.44 | 2.29 | 0.57 |
| AC | Harvard | *L. dealbatus* | 7682 | Dea 10-1 | 70.00 | 0.10 | 0.53 | 0.11 | 0.41 | 0.59 | 0.29 | 1.32 | 0.51 | 1.19 | 0.68 | 2.01 | 0.52 |
| AC | Harvard | *L. dealbatus* | 7682 | Dea 10-2 | 70.00 | 0.15 | 0.57 | 0.11 | 0.38 | 0.68 | 0.29 | 1.59 | 0.50 | 1.12 | 0.36 | 2.10 | 0.49 |
| AC | KIB | *L. echinotholus* | 501560 | Ech 1-1 | 80.00 | 0.14 | 0.74 | 0.06 | 0.31 | 1.01 | 0.43 | 2.15 | 0.65 | 0.79 | 0.56 | 2.79 | 0.57 |
| AC | KIB | *L. echinotholus* | 501558 | Ech 2-1 | 80.00 | 0.17 | 0.76 | 0.05 | 0.35 | 0.99 | 0.47 | 2.07 | 0.80 | 0.92 | 0.39 | 2.81 | 0.57 |
| AC | KIB | *L. echinotholus* | 501558 | Ech 2-2 | 70.00 | 0.14 | 0.62 | 0.06 | 0.27 | 0.84 | 0.38 | 1.98 | 0.61 | 0.76 | 0.27 | 2.49 | 0.65 |
| AC | Harvard | *L. edulis* | 25 | Edu 1-1 | 50.00 | 0.26 | 0.51 | 0.09 | 0.33 | 1.27 | 0.25 | 3.20 | 0.41 | 1.27 | 0.36 | 3.17 | 0.46 |
| AC | Harvard | *L. edulis* | 25 | Edu 1-2 | 47.50 | 0.23 | 0.60 | 0.13 | 0.43 | 1.57 | 0.31 | 3.28 | 0.39 | 1.14 | 0.50 | 3.39 | 0.52 |
| AC | Harvard | *L. edulis* | 25 | Edu 1-3 | 45.00 | 0.22 | 0.52 | 0.13 | 0.32 | 1.23 | 0.27 | 3.06 | 0.46 | 1.00 | 0.38 | 3.04 | 0.48 |
| AC | Harvard | *L. edulis* | 5805 | Edu 2-1 | 45.00 | 0.22 | 0.41 | 0.08 | 0.21 | 0.75 | 0.21 | 2.55 | 0.30 | 0.74 | 0.32 | 2.48 | 0.34 |
| AC | Harvard | *L. edulis* | 258 | Edu 3-1 | 50.00 | 0.16 | 0.63 | 0.06 | 0.42 | 1.11 | 0.36 | 2.32 | 0.54 | 1.03 | 0.39 | 2.68 | 0.57 |
| AC | Harvard | *L. edulis* | 258 | Edu 3-2 | 50.00 | 0.13 | 0.57 | 0.03 | 0.22 | 1.08 | 0.32 | 2.40 | 0.52 | 0.46 | 0.19 | 2.69 | 0.46 |
| AC | Harvard | *L. edulis* | 1250 | Edu 4-1 | 45.00 | 0.13 | 0.39 | 0.06 | 0.16 | 0.70 | 0.22 | 2.32 | 0.39 | 0.44 | 0.19 | 2.30 | 0.28 |
| AC | Harvard | *L. edulis* | 1250 | Edu 4-2 | 45.00 | 0.14 | 0.38 | 0.05 | 0.19 | 0.66 | 0.21 | 2.26 | 0.36 | 0.44 | 0.25 | 2.24 | 0.34 |
| AC | KIB | *L. elegans* | 501991 | Ele 1-1 | 52.50 | 0.21 | 0.55 | 0.08 | 0.30 | 0.65 | 0.28 | 1.83 | 0.50 | 0.68 | 0.40 | 2.13 | 0.42 |
| AC | KIB | *L. elegans* | 501991 | Ele 1-2 | 45.00 | 0.20 | 0.56 | 0.08 | 0.32 | 0.53 | 0.29 | 1.67 | 0.50 | 0.70 | 0.31 | 2.11 | 0.31 |
| AC | KIB | *L. elegans* | 501991 | Ele 1-3 | 46.50 | 0.30 | 0.62 | 0.09 | 0.42 | 0.70 | 0.33 | 2.16 | 0.42 | 0.90 | 0.37 | 2.26 | 0.51 |
| AC | KIB | *L. elegans* | 502006 | Ele 2-1 | 62.50 | 0.24 | 0.67 | 0.09 | 0.51 | 0.83 | 0.36 | 1.75 | 0.71 | 1.27 | 0.50 | 2.35 | 0.52 |
| AC | KIB | *L. elegans* | 501978 | Ele 3-1 | 52.50 | 0.22 | 0.58 | 0.08 | 0.43 | 0.61 | 0.31 | 1.75 | 0.50 | 0.90 | 0.38 | 2.06 | 0.49 |
| AC | KIB | *L. elegans* | 501978 | Ele 3-2 | 64.00 | 0.20 | 0.61 | 0.09 | 0.43 | 0.66 | 0.30 | 1.66 | 0.54 | 1.01 | 0.45 | 2.11 | 0.52 |
| AC | KIB | *L. elegans* | 501978 | Ele 3-3 | 67.50 | 0.18 | 0.54 | 0.06 | 0.36 | 0.56 | 0.28 | 1.51 | 0.46 | 0.84 | 0.32 | 1.94 | 0.46 |
| AC | KIB | *L. elegans* | 501986 | Ele 4-1 | 62.00 | 0.32 | 0.78 | 0.14 | 0.62 | 0.90 | 0.38 | 2.11 | 0.75 | 1.52 | 0.49 | 2.47 | 0.51 |
| AC | KIB | *L. elegans* | 501986 | Ele 4-2 | 60.00 | 0.27 | 0.61 | 0.08 | 0.40 | 0.65 | 0.33 | 1.96 | 0.52 | 0.89 | 0.44 | 2.16 | 0.48 |
| AC | KIB | *L. elegans* | 501986 | Ele 4-3 | 63.50 | 0.34 | 0.71 | 0.10 | 0.55 | 0.84 | 0.33 | 2.04 | 0.44 | 1.11 | 0.40 | 2.44 | 0.17 |
| AC | Field08 | *L. elegans* | 006HD | Ele 5-1 | 75.00 | 0.43 | 0.99 | 0.23 | 0.67 | 1.21 | 0.52 | 2.33 | 0.95 | 1.41 | 0.71 | 3.01 | 0.71 |
| AC | Field08 | *L. elegans* | 006HD | Ele 5-2 | 65.00 | 0.42 | 0.92 | 0.17 | 0.57 | 1.02 | 0.47 | 2.21 | 0.88 | 1.31 | 0.52 | 2.85 | 0.71 |
| AC | Field08 | *L. elegans* | 006HD | Ele 5-3 | 66.50 | 0.45 | 0.83 | 0.22 | 0.60 | 1.06 | 0.39 | 2.20 | 0.70 | 1.86 | 0.66 | 2.71 | 0.59 |
| AC | Leiden | *L. elegans* | Roelofsen6274 | Ele 6-1 | 70.00 | 0.47 | 1.03 | 0.15 | 0.73 | 1.52 | 0.55 | 2.63 | 0.67 | 1.35 | 0.41 | 3.42 | 0.90 |
| AC | Leiden | *L. elegans* | W.F.Winckel286 | Ele 7-1 | 65.00 | 0.44 | 0.92 | 0.23 | 0.54 | 1.40 | 0.47 | 2.70 | 0.79 | 1.30 | 0.66 | 3.19 | 0.59 |
| AC | Leiden | *L. elegans* | W.F.Winckel286 | Ele 7-2 | 69.14 | 0.39 | 0.87 | 0.18 | 0.55 | 1.14 | 0.43 | 2.49 | 0.74 | 1.19 | 0.48 | 2.81 | 0.68 |
| AC | Leiden | *L. elegans* | 1291877 | Ele 8-1 | 64.00 | 0.20 | 0.72 | 0.03 | 0.31 | 1.21 | 0.40 | 2.45 | 0.56 | 0.66 | 0.15 | 2.87 | 0.62 |
| AC | KIB | *L. elegans* | 501578 | Ele 9-1 | 70.00 | 0.43 | 0.78 | 0.14 | 0.50 | 1.10 | 0.39 | 2.34 | 0.66 | 0.99 | 0.36 | 2.82 | 0.54 |
| AC | KIB | *L. elegans* | 501578 | Ele 9-2 | 62.50 | 0.48 | 0.76 | 0.11 | 0.42 | 1.22 | 0.42 | 2.58 | 0.77 | 0.97 | 0.48 | 2.85 | 0.51 |
| AC | Harvard | *L. elegans* | NA | Ele 10-1 | 61.00 | 0.18 | 0.60 | 0.06 | 0.33 | 0.76 | 0.33 | 2.03 | 0.56 | 0.75 | 0.25 | 2.26 | 0.44 |
| AC | Harvard | *L. elegans* | NA | Ele 10-2 | 52.50 | 0.14 | 0.51 | 0.04 | 0.21 | 0.66 | 0.28 | 1.92 | 0.48 | 0.52 | 0.21 | 2.12 | 0.46 |
| AC | Harvard | *L. elegans* | NA | Ele 10-3 | 56.00 | 0.16 | 0.56 | 0.05 | 0.29 | 0.76 | 0.33 | 2.04 | 0.54 | 0.63 | 0.26 | 2.27 | 0.51 |
| AC | Harvard | *L. elegans* | 2387 | Ele 11-1 | 50.00 | 0.22 | 0.69 | 0.12 | 0.48 | 1.00 | 0.38 | 1.67 | 0.67 | 1.17 | 0.47 | 2.61 | 0.60 |
| AC | Harvard | *L. elegans* | 2387 | Ele 11-2 | 67.50 | 0.24 | 0.72 | 0.12 | 0.52 | 1.12 | 0.42 | 2.07 | 0.52 | 1.12 | 0.49 | 2.77 | 0.63 |
| AC | KIB | *L. elmerrillii* | 804689 | Elm 1-1 | 60.00 | 0.26 | 0.58 | 0.08 | 0.35 | 0.71 | 0.32 | 2.03 | 0.44 | 0.96 | 0.50 | 2.20 | 0.50 |
| AC | KIB | *L. elmerrillii* | 804689 | Elm 1-2 | 65.00 | 0.19 | 0.53 | 0.03 | 0.15 | 0.68 | 0.28 | 1.90 | 0.46 | 0.38 | 0.09 | 2.11 | 0.45 |
| AC | KIB | *L. elmerrillii* | 804689 | Elm 1-3 | 57.50 | 0.21 | 0.57 | 0.04 | 0.18 | 0.72 | 0.31 | 2.01 | 0.42 | 0.40 | 0.22 | 2.22 | 0.49 |
| AC | KIB | *L. elmerrillii* | 804689 | Elm 1-4 | 60.00 | 0.18 | 0.51 | 0.03 | 0.16 | 0.62 | 0.27 | 1.78 | 0.44 | 0.35 | 0.09 | 2.04 | 0.46 |
| AC | Leiden | *L. encleisocarpus* | Grashoff326 | Enc 1-1 | 54.00 | 0.22 | 0.62 | 0.04 | 0.33 | 0.79 | 0.38 | 2.10 | 0.60 | 0.68 | 0.26 | 2.35 | 0.54 |
| AC | Leiden | *L. encleisocarpus* | 326 | Enc 1-2 | 54.00 | 0.18 | 0.59 | 0.04 | 0.27 | 0.70 | 0.36 | 1.89 | 0.58 | 0.61 | 0.21 | 2.28 | 0.47 |
| AC | Leiden | *L. encleisocarpus* | 326 | Enc 1-3 | 55.67 | 0.23 | 0.55 | 0.04 | 0.27 | 0.69 | 0.32 | 1.97 | 0.59 | 0.62 | 0.16 | 2.17 | 0.45 |
| AC | Leiden | *L. encleisocarpus* | PaieS.15079 | Enc 2-1 | 57.50 | 0.25 | 0.67 | 0.05 | 0.28 | 1.09 | 0.36 | 1.97 | 0.62 | 0.63 | 0.20 | 2.51 | 0.47 |
| AC | Leiden | *L. ewyckii* | AsakakUnyongS.21194 | Ewy 1-1 | 62.50 | 0.26 | 0.87 | 0.13 | 0.37 | 1.32 | 0.57 | 2.53 | 0.89 | 1.15 | 0.39 | 3.66 | 0.68 |
| AC | KIB | *L. farinulentus* | 502481 | Far 1-1 | 50.00 | 0.06 | 0.40 | 0.02 | 0.16 | 0.33 | 0.24 | 1.41 | 0.39 | 0.38 | 0.25 | 1.62 | 0.33 |
| AC | KIB | *L. farinulentus* | 502481 | Far 1-2 | 50.00 | 0.06 | 0.37 | 0.02 | 0.17 | 0.32 | 0.24 | 1.43 | 0.35 | 0.40 | 0.19 | 1.68 | 0.37 |
| AC | KIB | *L. farinulentus* | 502481 | Far 1-3 | 50.00 | 0.07 | 0.39 | 0.03 | 0.19 | 0.35 | 0.24 | 1.48 | 0.35 | 0.39 | 0.19 | 1.66 | 0.38 |
| AC | KIB | *L. farinulentus* | 502481 | Far 1-4 | 52.50 | 0.07 | 0.42 | 0.05 | 0.24 | 0.43 | 0.25 | 1.67 | 0.31 | 0.49 | 0.32 | 1.76 | 0.39 |
| AC | KIB | *L. fenestratus* | 501610 | Fen 1-1 | 65.00 | 0.15 | 0.57 | 0.04 | 0.28 | 0.70 | 0.33 | 1.80 | 0.53 | 0.58 | 0.20 | 2.18 | 0.43 |
| AC | KIB | *L. fenestratus* | 501610 | Fen 1-2 | 61.00 | 0.13 | 0.51 | 0.05 | 0.29 | 0.59 | 0.30 | 1.58 | 0.50 | 0.60 | 0.22 | 2.05 | 0.46 |
| AC | KIB | *L. fenestratus* | 501610 | Fen 1-3 | 64.00 | 0.13 | 0.56 | 0.04 | 0.24 | 0.62 | 0.32 | 1.79 | 0.53 | 0.61 | 0.22 | 2.05 | 0.57 |
| AC | KIB | *L. fenestratus* | 501614 | Fen 2-1 | 60.00 | 0.08 | 0.38 | 0.01 | 0.13 | 0.36 | 0.21 | 1.45 | 0.40 | 0.29 | 0.21 | 1.64 | 0.35 |
| AC | KIB | *L. fenestratus* | 501614 | Fen 2-2 | 60.00 | 0.10 | 0.40 | 0.03 | 0.15 | 0.38 | 0.23 | 1.48 | 0.37 | 0.37 | 0.10 | 1.65 | 0.34 |
| AC | KIB | *L. fenestratus* | 501614 | Fen 2-3 | 59.00 | 0.08 | 0.35 | 0.02 | 0.12 | 0.32 | 0.19 | 1.26 | 0.32 | 0.30 | 0.12 | 1.45 | 0.31 |
| AC | KIB | *L. fenestratus* | 501705 | Fen 3-1 | 73.50 | 0.16 | 0.61 | 0.04 | 0.23 | 0.62 | 0.36 | 1.84 | 0.57 | 0.53 | 0.34 | 2.20 | 0.48 |
| AC | KIB | *L. fenestratus* | 501753 | Fen 3-2 | 65.00 | 0.23 | 0.70 | 0.05 | 0.38 | 0.84 | 0.38 | 1.80 | 0.67 | 0.75 | 0.34 | 2.48 | 0.65 |
| AC | KIB | *L. fenestratus* | 501753 | Fen 3-3 | 60.00 | 0.25 | 0.59 | 0.07 | 0.38 | 0.76 | 0.31 | 1.85 | 0.63 | 1.07 | 0.42 | 2.28 | 0.44 |
| AC | KIB | *L. fenestratus* | 501753 | Fen 3-4 | 60.00 | 0.25 | 0.58 | 0.10 | 0.37 | 0.61 | 0.30 | 1.61 | 0.50 | 0.96 | 0.53 | 2.04 | 0.52 |
| AC | KIB | *L. fenestratus* | 501753 | Fen 3-5 | 59.50 | 0.17 | 0.57 | 0.07 | 0.37 | 0.76 | 0.33 | 1.76 | 0.60 | 0.77 | 0.44 | 2.28 | 0.50 |
| AC | KIB | *L. fenestratus* | 501754 | Fen 4-1 | 67.50 | 0.20 | 0.69 | 0.07 | 0.45 | 0.82 | 0.39 | 1.79 | 0.54 | 0.90 | 0.44 | 2.45 | 0.52 |
| AC | KIB | *L. fenestratus* | 501754 | Fen 4-2 | 65.00 | 0.21 | 0.67 | 0.05 | 0.35 | 0.75 | 0.37 | 1.73 | 0.66 | 0.70 | 0.54 | 2.45 | 0.57 |
| AC | KIB | *L. fenestratus* | 786646 | Fen 5-1 | 70.00 | 0.15 | 0.56 | 0.05 | 0.28 | 0.61 | 0.33 | 1.69 | 0.54 | 0.59 | 0.30 | 2.05 | 0.52 |
| AC | KIB | *L. fenestratus* | 786646 | Fen 5-2 | 65.00 | 0.13 | 0.57 | 0.04 | 0.23 | 0.63 | 0.34 | 1.70 | 0.55 | 0.50 | 0.26 | 2.09 | 0.48 |
| AC | KIB | *L. fenestratus* | 501718 | Fen 6-1 | 56.00 | 0.18 | 0.62 | 0.07 | 0.36 | 0.89 | 0.35 | 2.23 | 0.54 | 0.75 | 0.28 | 2.46 | 0.60 |
| AC | KIB | *L. fenestratus* | 501717 | Fen 7-1 | 57.50 | 0.12 | 0.56 | 0.04 | 0.32 | 0.68 | 0.34 | 1.76 | 0.47 | 0.74 | 0.38 | 2.20 | 0.50 |
| AC | KIB | *L. fenestratus* | 501717 | Fen 7-2 | 57.50 | 0.10 | 0.55 | 0.03 | 0.26 | 0.70 | 0.34 | 1.76 | 0.54 | 0.67 | 0.14 | 2.19 | 0.51 |
| AC | KIB | *L. fenestratus* | 501717 | Fen 7-3 | 53.50 | 0.11 | 0.57 | 0.03 | 0.28 | 0.70 | 0.33 | 1.84 | 0.53 | 0.66 | 0.26 | 2.19 | 0.50 |
| AC | Leiden | *L. ferrugineus* | Abd.Rahimetal92961 | Fer 1-1 | 56.00 | 0.10 | 0.51 | 0.05 | 0.31 | 0.47 | 0.31 | 1.54 | 0.58 | 0.64 | 0.11 | 1.85 | 0.50 |
| AC | Leiden | *L. ferrugineus* | CWL1082 | Fer 2-1 | 55.00 | 0.11 | 0.53 | 0.03 | 0.28 | 0.63 | 0.32 | 1.71 | 0.53 | 0.66 | 0.28 | 2.07 | 0.51 |
| AC | Leiden | *L. ferrugineus* | Dyg.Awa&YiiP.C.46720 | Fer 3-1 | 47.50 | 0.10 | 0.63 | 0.06 | 0.34 | 0.61 | 0.33 | 1.85 | 0.74 | 0.78 | 0.41 | 2.25 | 0.71 |
| AC | Leiden | *L. ferrugineus* | Aban GibotSAN100016 | Fer 4-1 | 54.00 | 0.10 | 0.57 | 0.08 | 0.30 | 0.56 | 0.37 | 1.69 | 0.66 | 0.74 | 0.43 | 2.17 | 0.60 |
| AC | Leiden | *L. ferrugineus* | W.L.Chew1802R | Fer 5-1 | 50.00 | 0.11 | 0.54 | 0.05 | 0.35 | 0.62 | 0.33 | 1.78 | 0.46 | 0.69 | 0.29 | 2.09 | 0.44 |
| AC | Leiden | *L. ferrugineus* | JugahakKudi | Fer 6-1 | 55.00 | 0.12 | 0.54 | 0.02 | 0.23 | 0.71 | 0.30 | 1.80 | 0.57 | 0.50 | 0.25 | 2.24 | 0.53 |
| AC | Leiden | *L. ferrugineus* | JugahakKudi | Fer 6-2 | 47.50 | 0.08 | 0.52 | 0.05 | 0.34 | 0.60 | 0.34 | 1.93 | 0.42 | 0.83 | 0.26 | 2.36 | 0.50 |
| AC | Leiden | *L. ferrugineus* | S.Lantoh67719M | Fer 7-1 | 55.00 | 0.10 | 0.58 | 0.08 | 0.22 | 0.58 | 0.38 | 1.72 | 0.24 | 0.61 | 0.14 | 2.26 | 0.40 |
| AC | Leiden | *L. ferrugineus* | Tuyuk,Donggop&Amin94395L | Fer 8-1 | 52.50 | 0.11 | 0.63 | 0.07 | 0.48 | 0.65 | 0.34 | 1.79 | 0.66 | 0.94 | 0.23 | 2.12 | 0.43 |
| AC | Leiden | *L. ferrugineus* | 57078 | Fer 9-1 | 47.50 | 0.11 | 0.62 | 0.04 | 0.36 | 0.69 | 0.35 | 1.75 | 0.52 | 0.77 | 0.30 | 2.29 | 0.53 |
| AC | Harvard | *L. fohaiensis* | 1995 1-24-22 | Foh 1-1 | 62.50 | 0.18 | 0.61 | 0.06 | 0.36 | 0.62 | 0.36 | 1.59 | 0.50 | 0.68 | 0.42 | 2.17 | 0.44 |
| AC | Harvard | *L. fohaiensis* | 1995 1-24-22 | Foh 1-2 | 62.50 | 0.20 | 0.67 | 0.07 | 0.36 | 0.71 | 0.36 | 1.75 | 0.65 | 0.73 | 0.32 | 2.28 | 0.61 |
| AC | Harvard | *L. fohaiensis* | 1995 1-24-22 | Foh 1-3 | 70.00 | 0.24 | 0.67 | 0.07 | 0.37 | 0.74 | 0.38 | 1.88 | 0.49 | 0.82 | 0.48 | 2.28 | 0.64 |
| AC | Harvard | *L. fohaiensis* | 79438 | Foh 2-1 | 70.00 | 0.26 | 0.73 | 0.10 | 0.50 | 1.05 | 0.39 | 2.10 | 0.73 | 1.25 | 0.61 | 2.62 | 0.53 |
| AC | Harvard | *L. fohaiensis* | 79438 | Foh 2-2 | 65.00 | 0.27 | 0.80 | 0.12 | 0.62 | 1.16 | 0.45 | 2.20 | 0.67 | 1.31 | 0.62 | 2.90 | 0.66 |
| AC | Harvard | *L. fohaiensis* | 79438 | Foh 2-3 | 70.00 | 0.20 | 0.68 | 0.07 | 0.40 | 0.74 | 0.42 | 1.78 | 0.61 | 0.85 | 0.31 | 2.38 | 0.55 |
| ER | Harvard | *L. fohaiensis* | 74635 | Foh 3-1 | 82.50 | 0.09 | 0.60 | 0.86 | 0.53 | 0.30 | 0.23 | 1.02 | 0.59 | 1.65 | 0.37 | 1.72 | 0.31 |
| ER | Harvard | *L. fohaiensis* | 74635 | Foh 3-2 | 85.00 | 0.12 | 0.58 | 0.60 | 0.50 | 0.36 | 0.30 | 0.98 | 0.47 | 1.52 | 0.44 | 1.67 | 0.42 |
| AC | Harvard | *L. formosanus* | 1594 | Foh 4-1 | 57.00 | 0.31 | 0.63 | 0.07 | 0.41 | 0.79 | 0.33 | 2.19 | 0.55 | 0.92 | 0.27 | 2.34 | 0.49 |
| AC | Harvard | *L. formosanus* | 1594 | Foh 4-2 | 55.00 | 0.23 | 0.59 | 0.05 | 0.32 | 0.72 | 0.30 | 2.09 | 0.56 | 0.63 | 0.39 | 2.19 | 0.48 |
| AC | Harvard | *L. glaber* | 15744 | Gla 1-1 | 62.50 | 0.11 | 0.44 | 0.04 | 0.24 | 0.68 | 0.26 | 1.99 | 0.46 | 0.57 | 0.27 | 2.12 | 0.35 |
| AC | Harvard | *L. glaber* | 15744 | Gla 1-2 | 65.00 | 0.12 | 0.49 | 0.04 | 0.19 | 0.57 | 0.30 | 1.78 | 0.49 | 0.46 | 0.30 | 2.07 | 0.43 |
| AC | Harvard | *L. glaber* | 218 | Gla 2-1 | 55.00 | 0.17 | 0.44 | 0.04 | 0.17 | 0.80 | 0.26 | 2.20 | 0.39 | 0.49 | 0.11 | 2.34 | 0.46 |
| AC | Harvard | *L. glaber* | 218 | Gla 2-2 | 60.00 | 0.23 | 0.49 | 0.03 | 0.16 | 0.77 | 0.24 | 2.13 | 0.44 | 0.36 | 0.18 | 2.29 | 0.42 |
| AC | KIB | *L. glaber* | 501872 | Gla 3-1 | 47.50 | 0.25 | 0.43 | 0.03 | 0.16 | 0.68 | 0.23 | 2.26 | 0.35 | 0.41 | 0.12 | 2.19 | 0.32 |
| AC | KIB | *L. glaber* | 501872 | Gla 3-2 | 50.00 | 0.18 | 0.34 | 0.02 | 0.14 | 0.55 | 0.20 | 2.05 | 0.30 | 0.29 | 0.15 | 2.02 | 0.24 |
| AC | KIB | *L. glaber* | 501872 | Gla 3-3 | 52.50 | 0.20 | 0.39 | 0.04 | 0.17 | 0.62 | 0.20 | 2.19 | 0.39 | 0.42 | 0.18 | 2.16 | 0.27 |
| AC | KIB | *L. glaber* | 501894 | Gla 4-1 | 60.00 | 0.16 | 0.47 | 0.05 | 0.19 | 0.58 | 0.24 | 1.89 | 0.39 | 0.46 | 0.21 | 2.05 | 0.44 |
| AC | KIB | *L. glaber* | 501902 | Gla 5-1 | 57.50 | 0.15 | 0.43 | 0.05 | 0.14 | 0.60 | 0.24 | 1.96 | 0.40 | 0.41 | 0.18 | 2.06 | 0.37 |
| AC | KIB | *L. glaber* | 501902 | Gla 5-2 | 52.50 | 0.16 | 0.44 | 0.03 | 0.15 | 0.57 | 0.23 | 1.89 | 0.36 | 0.35 | 0.14 | 2.07 | 0.31 |
| AC | KIB | *L. glaber* | 501908 | Gla 6-1 | 50.00 | 0.28 | 0.50 | 0.04 | 0.13 | 1.05 | 0.28 | 2.82 | 0.46 | 0.37 | 0.09 | 2.77 | 0.44 |
| AC | KIB | *L. glaber* | 501908 | Gla 6-2 | 55.00 | 0.31 | 0.47 | 0.06 | 0.15 | 1.01 | 0.27 | 2.95 | 0.43 | 0.40 | 0.14 | 2.82 | 0.49 |
| AC | Leiden | *L. glutinosus* | Elmer20971A | Glu 1-1 | 80.00 | 0.22 | 0.70 | 0.08 | 0.43 | 0.65 | 0.47 | 1.61 | 0.74 | 0.90 | 0.46 | 2.50 | 0.66 |
| AC | Leiden | *L. gracilis* | Ashton19449 | Gra 1-1 | 50.00 | 0.13 | 0.63 | 0.05 | 0.30 | 0.95 | 0.38 | 1.99 | 0.63 | 0.79 | 0.36 | 2.64 | 0.56 |
| AC | Leiden | *L. gracilis* | Beccari4031 | Gra 2-1 | 47.50 | 0.16 | 0.80 | 0.06 | 0.44 | 1.10 | 0.45 | 2.26 | 0.77 | 1.03 | 0.25 | 2.90 | 0.70 |
| AC | Leiden | *L. gracilis* | Clemens26716 | Gra 3-1 | 48.50 | 0.11 | 0.51 | 0.06 | 0.27 | 0.63 | 0.31 | 1.99 | 0.42 | 0.78 | 0.29 | 2.32 | 0.51 |
| AC | Leiden | *L. gracilis* | CWL1039 | Gra 4-1 | 50.00 | 0.09 | 0.55 | 0.06 | 0.23 | 0.70 | 0.35 | 1.71 | 0.49 | 0.84 | 0.30 | 2.41 | 0.35 |
| AC | Leiden | *L. gracilis* | PaieS.15588 | Gra 5-1 | 52.50 | 0.05 | 0.37 | 0.02 | 0.16 | 0.30 | 0.29 | 1.38 | 0.46 | 0.53 | 0.20 | 1.91 | 0.36 |
| AC | Leiden | *L. gracilis* | Villamil216 | Gra 6-1 | 53.50 | 0.09 | 0.57 | 0.04 | 0.30 | 0.73 | 0.34 | 2.05 | 0.51 | 0.74 | 0.33 | 2.43 | 0.45 |
| AC | Leiden | *L. gracilis* | Villamil50 | Gra 6-2 | 52.00 | 0.13 | 0.60 | 0.03 | 0.27 | 0.71 | 0.36 | 1.96 | 0.47 | 0.67 | 0.27 | 2.53 | 0.44 |
| AC | Leiden | *L. gracilis* | Wood3469 | Gra 7-1 | 44.00 | 0.15 | 0.65 | 0.07 | 0.36 | 0.96 | 0.37 | 2.15 | 0.55 | 0.87 | 0.18 | 2.70 | 0.52 |
| AC | Leiden | *L. gracilis* | WoodSan.A4039 | Gra 8-1 | 51.50 | 0.09 | 0.55 | 0.06 | 0.28 | 0.61 | 0.35 | 0.19 | 0.51 | 0.78 | 0.40 | 2.39 | 0.50 |
| AC | Harvard | *L. hancei* | 10280 | Han 1-1 | 57.50 | 0.32 | 0.63 | 0.03 | 0.19 | 1.02 | 0.33 | 2.44 | 0.54 | 0.41 | 0.17 | 2.62 | 0.51 |
| AC | Harvard | *L. hancei* | 10280 | Han 1-2 | 60.00 | 0.20 | 0.61 | 0.02 | 0.13 | 1.04 | 0.34 | 2.42 | 0.50 | 0.34 | 0.19 | 2.61 | 0.50 |
| AC | Harvard | *L. hancei* | 11165 | Han 2-1 | 62.50 | 0.12 | 0.44 | 0.02 | 0.23 | 0.65 | 0.25 | 1.96 | 0.47 | 0.45 | 0.20 | 2.14 | 0.41 |
| AC | Harvard | *L. hancei* | 11165 | Han 2-2 | 45.00 | 0.27 | 0.58 | 0.06 | 0.20 | 1.13 | 0.33 | 2.75 | 0.58 | 0.50 | 0.25 | 2.78 | 0.49 |
| AC | KIB | *L. hancei* | 502027 | Han 3-1 | 57.50 | 0.15 | 0.53 | 0.02 | 0.21 | 0.72 | 0.30 | 1.97 | 0.47 | 2.20 | 0.35 | 0.50 | 0.53 |
| AC | KIB | *L. hancei* | 502027 | Han 3-2 | 70.00 | 0.24 | 0.65 | 0.06 | 0.39 | 0.86 | 0.37 | 2.19 | 0.53 | 0.88 | 0.54 | 2.43 | 0.51 |
| AC | KIB | *L. hancei* | 502027 | Han 3-3 | 58.50 | 0.19 | 0.59 | 0.06 | 0.41 | 0.74 | 0.32 | 1.94 | 0.50 | 0.87 | 0.36 | 2.22 | 0.47 |
| AC | KIB | *L. hancei* | 502029 | Han 4-1 | 65.00 | 0.17 | 0.61 | 0.05 | 0.32 | 0.82 | 0.36 | 2.09 | 0.58 | 0.70 | 0.26 | 2.39 | 0.49 |
| AC | KIB | *L. hancei* | 502029 | Han 4-2 | 65.00 | 0.24 | 0.66 | 0.05 | 0.39 | 1.00 | 0.37 | 2.31 | 0.48 | 1.02 | 0.07 | 2.51 | 0.54 |
| AC | KIB | *L. hancei* | 695486 | Han 5-1 | 63.50 | 0.14 | 0.56 | 0.04 | 0.29 | 0.75 | 0.34 | 2.00 | 0.55 | 0.61 | 0.20 | 2.24 | 0.51 |
| AC | KIB | *L. hancei* | 695486 | Han 5-2 | 61.00 | 0.11 | 0.53 | 0.02 | 0.20 | 0.73 | 0.33 | 2.05 | 0.46 | 0.51 | 0.26 | 2.19 | 0.47 |
| AC | KIB | *L. hancei* | 695486 | Han 5-3 | 62.50 | 0.13 | 0.65 | 0.04 | 0.30 | 0.90 | 0.40 | 2.13 | 0.52 | 0.60 | 0.37 | 2.58 | 0.61 |
| AC | KIB | *L. hancei* | 695486 | Han 5-4 | 60.00 | 0.10 | 0.54 | 0.03 | 0.21 | 0.79 | 0.33 | 2.05 | 0.52 | 0.47 | 0.11 | 2.40 | 0.53 |
| AC | KIB | *L. hancei* | 502038 | Han 6-1 | 60.00 | 0.09 | 0.43 | 0.02 | 0.19 | 0.57 | 0.25 | 1.77 | 0.40 | 0.49 | 0.12 | 1.93 | 0.40 |
| AC | KIB | *L. hancei* | 502038 | Han 6-2 | 57.50 | 0.08 | 0.46 | 0.02 | 0.16 | 0.62 | 0.29 | 1.79 | 0.37 | 0.38 | 0.26 | 2.03 | 0.48 |
| AC | KIB | *L. hancei* | 502038 | Han 6-3 | 55.00 | 0.11 | 0.49 | 0.02 | 0.18 | 0.63 | 0.25 | 1.89 | 0.42 | 0.39 | 0.13 | 2.09 | 0.39 |
| AC | KIB | *L. hancei* | 691522 | Han 7-1 | 52.50 | 0.14 | 0.52 | 0.05 | 0.27 | 0.75 | 0.29 | 2.11 | 0.52 | 0.67 | 0.43 | 2.24 | 0.46 |
| AC | KIB | *L. hancei* | 691522 | Han 7-2 | 56.50 | 0.14 | 0.49 | 0.05 | 0.26 | 0.83 | 0.33 | 2.24 | 0.53 | 0.58 | 0.29 | 2.36 | 0.45 |
| AC | KIB | *L. hancei* | 691518 | Han 8-1 | 55.00 | 0.24 | 0.66 | 0.06 | 0.43 | 1.01 | 0.36 | 2.36 | 0.65 | 0.96 | 0.51 | 2.54 | 0.60 |
| AC | KIB | *L. hancei* | 691518 | Han 8-2 | 50.00 | 0.21 | 0.58 | 0.04 | 0.35 | 0.87 | 0.31 | 2.20 | 0.57 | 0.66 | 0.36 | 2.45 | 0.57 |
| AC | Field08 | *L. hancei* | HA-09 | Han 9-1 | 60.00 | 0.20 | 0.64 | 0.04 | 0.26 | 1.00 | 0.37 | 2.34 | 0.69 | 0.67 | 0.32 | 2.54 | 0.59 |
| AC | Field08 | *L. hancei* | HA-09 | Han 9-2 | 55.00 | 0.15 | 0.56 | 0.03 | 0.24 | 0.69 | 0.32 | 1.76 | 0.53 | 0.48 | 0.07 | 2.17 | 0.48 |
| AC | Field08 | *L. hancei* | HA-09 | Han 9-3 | 62.50 | 0.18 | 0.59 | 0.04 | 0.25 | 0.96 | 0.36 | 2.24 | 0.58 | 0.56 | 0.35 | 2.53 | 0.57 |
| AC | Field08 | *L. hancei* | AL-HA-05 | Han 10-1 | 67.50 | 0.25 | 0.76 | 0.11 | 0.47 | 1.10 | 0.44 | 2.38 | 0.69 | 1.13 | 0.64 | 2.77 | 0.56 |
| AC | Field08 | *L. hancei* | AL-HA-05 | Han 10-2 | 60.00 | 0.21 | 0.64 | 0.07 | 0.33 | 0.82 | 0.40 | 2.09 | 0.59 | 0.76 | 0.29 | 2.53 | 0.59 |
| AC | Field08 | *L. hancei* | AL-HA-05 | Han 10-3 | 65.00 | 0.23 | 0.71 | 0.09 | 0.46 | 1.10 | 0.42 | 2.37 | 0.66 | 1.05 | 0.43 | 2.72 | 0.56 |
| AC | Field08 | *L. hancei* | AL-HA-01 | Han 11-1 | 60.00 | 0.20 | 0.57 | 0.04 | 0.35 | 0.70 | 0.30 | 1.92 | 0.38 | 0.75 | 0.30 | 2.14 | 0.53 |
| AC | Field08 | *L. hancei* | AL-HA-01 | Han 11-2 | 55.00 | 0.18 | 0.52 | 0.04 | 0.31 | 0.75 | 0.31 | 2.06 | 0.49 | 0.72 | 0.39 | 2.22 | 0.45 |
| AC | Field08 | *L. hancei* | AL-HA-01 | Han 11-3 | 55.00 | 0.17 | 0.57 | 0.05 | 0.33 | 0.76 | 0.32 | 2.08 | 0.55 | 0.71 | 0.27 | 2.26 | 0.56 |
| AC | Field08 | *L. hancei* | HA-DNA-05 | Han 12-1 | 59.00 | 0.17 | 0.55 | 0.04 | 0.32 | 0.67 | 0.31 | 1.87 | 0.52 | 0.72 | 0.35 | 2.10 | 0.49 |
| AC | Field08 | *L. hancei* | HA-DNA-05 | Han 12-2 | 65.00 | 0.10 | 0.51 | 0.04 | 0.28 | 0.58 | 0.31 | 1.75 | 0.50 | 0.60 | 0.20 | 1.99 | 0.49 |
| AC | Field08 | *L. hancei* | HA-DNA-05 | Han 12-3 | 62.50 | 0.14 | 0.54 | 0.05 | 0.28 | 0.61 | 0.30 | 1.75 | 0.44 | 0.66 | 0.27 | 2.03 | 0.54 |
| AC | Field08 | *L. hancei* | AL-HA-02 | Han 13-1 | 62.50 | 0.15 | 0.63 | 0.34 | 0.27 | 0.88 | 0.38 | 2.13 | 0.57 | 0.64 | 0.38 | 2.47 | 0.48 |
| AC | Field08 | *L. hancei* | AL-HA-02 | Han 13-2 | 60.00 | 0.07 | 0.60 | 0.02 | 0.24 | 0.71 | 0.35 | 1.81 | 0.55 | 0.54 | 0.13 | 2.33 | 0.58 |
| AC | Field08 | *L. hancei* | AL-HA-02 | Han 13-3 | 57.50 | 0.16 | 0.65 | 0.03 | 0.33 | 0.90 | 0.36 | 2.08 | 0.56 | 0.60 | 0.29 | 2.47 | 0.54 |
| AC | Field08 | *L. hancei* | AL-HA-03 | Han 14-1 | 50.00 | 0.09 | 0.40 | 0.04 | 0.27 | 0.39 | 0.22 | 1.49 | 0.35 | 0.64 | 0.25 | 1.59 | 0.33 |
| AC | Field08 | *L. hancei* | AL-HA-03 | Han 14-2 | 42.50 | 0.14 | 0.44 | 0.06 | 0.26 | 0.63 | 0.23 | 2.07 | 0.41 | 0.74 | 0.44 | 2.07 | 0.39 |
| AC | Field08 | *L. hancei* | AL-HA-03 | Han 14-3 | 50.00 | 0.17 | 0.59 | 0.05 | 0.24 | 0.96 | 0.33 | 2.28 | 0.62 | 0.59 | 0.22 | 2.53 | 0.53 |
| AC | Field08 | *L. hancei* | HA-DNA-11 | Han 15-1 | 57.50 | 0.16 | 0.59 | 0.06 | 0.33 | 0.78 | 0.34 | 2.12 | 0.55 | 2.33 | 0.25 | 0.83 | 0.52 |
| AC | Field08 | *L. hancei* | HA-DNA-11 | Han 15-2 | 65.00 | 0.15 | 0.64 | 0.06 | 0.31 | 0.93 | 0.38 | 2.17 | 0.57 | 0.65 | 0.08 | 2.49 | 0.52 |
| AC | Field08 | *L. hancei* | HA-DNA-11 | Han 15-3 | 62.50 | 0.16 | 0.67 | 0.06 | 0.30 | 0.90 | 0.38 | 2.14 | 0.66 | 0.91 | 0.32 | 2.49 | 0.58 |
| AC | Field08 | *L. hancei* | AL-HA-06 | Han 16-1 | 60.00 | 0.22 | 0.74 | 0.06 | 0.33 | 0.93 | 0.46 | 2.24 | 0.62 | 0.71 | 0.05 | 2.66 | 0.67 |
| AC | Field08 | *L. hancei* | AL-HA-06 | Han 16-2 | 65.00 | 0.17 | 0.56 | 0.06 | 0.33 | 0.72 | 0.32 | 1.97 | 0.51 | 0.89 | 0.40 | 2.21 | 0.44 |
| AC | Field08 | *L. hancei* | AL-HA-04 | Han 17-1 | 65.00 | 0.20 | 0.69 | 0.06 | 0.45 | 0.92 | 0.38 | 2.14 | 0.60 | 1.21 | 0.52 | 2.46 | 0.48 |
| AC | Field08 | *L. hancei* | AL-HA-04 | Han 17-2 | 65.00 | 0.17 | 0.69 | 0.07 | 0.46 | 0.94 | 0.35 | 2.17 | 0.70 | 1.07 | 0.49 | 2.49 | 0.18 |
| AC | Field08 | *L. hancei* | AL-HA-04 | Han 17-3 | 64.00 | 0.15 | 0.63 | 0.04 | 0.35 | 0.83 | 0.34 | 2.02 | 0.58 | 0.75 | 0.49 | 2.32 | 0.48 |
| AC | Field08 | *L. hancei* | HA-DNA-04 | Han 17-4 | 62.50 | 0.19 | 0.62 | 0.06 | 0.38 | 0.84 | 0.33 | 2.15 | 0.62 | 1.06 | 0.38 | 2.42 | 0.45 |
| AC | Field08 | *L. hancei* | HA-DNA-04 | Han 17-5 | 62.50 | 0.16 | 0.55 | 0.05 | 0.33 | 0.72 | 0.30 | 2.00 | 0.43 | 0.92 | 0.33 | 2.18 | 0.45 |
| AC | Field08 | *L. hancei* | HA-DNA-04 | Han 17-6 | 57.50 | 0.19 | 0.59 | 0.04 | 0.43 | 0.87 | 0.32 | 2.21 | 0.54 | 0.95 | 0.54 | 2.37 | 0.57 |
| AC | Field08 | *L. hancei* | AL-HA-07 | Han 18-1 | 65.00 | 0.27 | 0.83 | 0.12 | 0.57 | 1.25 | 0.45 | 2.53 | 0.71 | 1.20 | 0.30 | 2.90 | 0.73 |
| AC | Field08 | *L. hancei* | AL-HA-07 | Han 18-2 | 65.00 | 0.28 | 0.94 | 0.12 | 0.53 | 1.36 | 0.54 | 2.54 | 0.66 | 1.23 | 0.78 | 3.11 | 0.83 |
| AC | Field08 | *L. hancei* | AL-HA-07 | Han 18-3 | 65.00 | 0.20 | 0.73 | 0.13 | 0.58 | 0.98 | 0.39 | 2.23 | 0.60 | 1.38 | 0.63 | 2.54 | 0.63 |
| AC | Field08 | *L. hancei* | HA-DNA-03 | Han 19-1 | 65.00 | 0.20 | 0.70 | 0.05 | 0.43 | 1.05 | 0.39 | 2.33 | 0.70 | 0.81 | 0.30 | 2.67 | 0.57 |
| AC | Field08 | *L. hancei* | HA-DNA-03 | Han 19-2 | 60.00 | 0.21 | 0.63 | 0.04 | 0.42 | 1.00 | 0.37 | 2.26 | 0.58 | 0.75 | 0.47 | 2.55 | 0.46 |
| AC | Field08 | *L. hancei* | HA-DNA-03 | Han 19-3 | 57.50 | 0.20 | 0.63 | 0.03 | 0.33 | 0.94 | 0.35 | 2.21 | 0.59 | 0.61 | 0.31 | 2.49 | 0.55 |
| AC | Field08 | *L. hancei* | HA-DNA-10 | Han 20-1 | 52.50 | 0.23 | 0.78 | 0.13 | 0.40 | 1.01 | 0.37 | 2.32 | 0.68 | 1.07 | 0.50 | 2.58 | 0.57 |
| AC | Field08 | *L. hancei* | HA-DNA-10 | Han 20-2 | 57.50 | 0.21 | 0.64 | 0.08 | 0.35 | 0.99 | 0.36 | 2.26 | 0.53 | 0.76 | 0.29 | 2.58 | 0.53 |
| AC | Field08 | *L. hancei* | 022HD | Han 21-1 | 45.00 | 0.13 | 0.48 | 0.04 | 0.23 | 0.73 | 0.27 | 2.02 | 0.44 | 0.59 | 0.16 | 2.23 | 0.41 |
| AC | Field08 | *L. hancei* | 022HD | Han 21-2 | 50.00 | 0.17 | 0.54 | 0.05 | 0.27 | 0.84 | 0.31 | 2.19 | 0.46 | 0.90 | 0.47 | 2.40 | 0.40 |
| AC | Field08 | *L. hancei* | 032HD | Han 22-1 | 55.00 | 0.16 | 0.60 | 0.05 | 0.37 | 0.76 | 0.31 | 1.98 | 0.49 | 0.80 | 0.48 | 2.24 | 0.54 |
| AC | Field08 | *L. hancei* | 032HD | Han 22-2 | 52.50 | 0.17 | 0.57 | 0.05 | 0.26 | 0.80 | 0.33 | 2.01 | 0.47 | 0.55 | 0.34 | 2.35 | 0.46 |
| AC | Field08 | *L. hancei* | 025HD | Han 23-1 | 65.00 | 0.20 | 0.75 | 0.06 | 0.47 | 1.06 | 0.42 | 2.25 | 0.74 | 0.95 | 0.44 | 2.75 | 0.51 |
| AC | Field08 | *L. hancei* | 025HD | Han 23-2 | 67.50 | 0.25 | 0.81 | 0.08 | 0.45 | 1.15 | 0.49 | 2.44 | 0.74 | 0.96 | 0.58 | 2.88 | 0.75 |
| AC | Field08 | *L. hancei* | 025HD | Han 23-3 | 62.50 | 0.19 | 0.73 | 0.05 | 0.38 | 0.93 | 0.43 | 2.25 | 0.68 | 0.88 | 0.47 | 2.63 | 0.72 |
| AC | Field08 | *L. hancei* | 023HD | Han 24-1 | 60.00 | 0.22 | 0.64 | 0.07 | 0.31 | 1.05 | 0.36 | 2.28 | 0.67 | 0.77 | 0.17 | 2.61 | 0.55 |
| AC | Field08 | *L. hancei* | 023HD | Han 24-2 | 55.00 | 0.19 | 0.59 | 0.08 | 0.36 | 0.94 | 0.32 | 2.24 | 0.55 | 0.83 | 0.17 | 2.45 | 0.53 |
| AC | Field08 | *L. hancei* | 023HD | Han 24-3 | 57.50 | 0.20 | 0.60 | 0.04 | 0.26 | 0.97 | 0.32 | 2.26 | 0.49 | 0.58 | 0.14 | 2.54 | 0.34 |
| AC | Field08 | *L. hancei* | 034HD | Han 25-1 | 56.50 | 0.20 | 0.61 | 0.05 | 0.39 | 0.95 | 0.36 | 2.23 | 0.64 | 0.75 | 0.36 | 2.52 | 0.48 |
| AC | Field08 | *L. hancei* | 034HD | Han 25-2 | 57.50 | 0.19 | 0.64 | 0.05 | 0.37 | 1.01 | 0.37 | 2.48 | 0.57 | 0.76 | 0.28 | 2.62 | 0.62 |
| AC | Field08 | *L. hancei* | 034HD | Han 25-3 | 57.50 | 0.24 | 0.69 | 0.08 | 0.40 | 1.10 | 0.37 | 2.47 | 0.34 | 0.92 | 0.30 | 2.65 | 0.51 |
| AC | Field08 | *L. hancei* | 021HD | Han 26-1 | 57.50 | 0.15 | 0.61 | 0.05 | 0.33 | 0.94 | 0.36 | 2.19 | 0.58 | 0.68 | 0.38 | 2.54 | 0.47 |
| AC | Field08 | *L. hancei* | 021HD | Han 26-2 | 62.50 | 0.17 | 0.61 | 0.05 | 0.25 | 0.64 | 0.34 | 1.82 | 0.53 | 0.61 | 0.12 | 2.15 | 0.46 |
| AC | Field08 | *L. hancei* | 020HD | Han 27-1 | 65.00 | 0.20 | 0.66 | 0.04 | 0.25 | 1.05 | 0.40 | 2.42 | 0.62 | 0.54 | 0.33 | 2.80 | 0.51 |
| AC | Field08 | *L. hancei* | 020HD | Han 27-2 | 65.00 | 0.18 | 0.69 | 0.06 | 0.29 | 1.05 | 0.39 | 2.34 | 0.65 | 0.64 | 0.26 | 2.67 | 0.55 |
| AC | Field08 | *L. hancei* | 020HD | Han 27-3 | 63.50 | 0.29 | 0.77 | 0.04 | 0.29 | 1.19 | 0.42 | 2.42 | 0.73 | 0.62 | 0.09 | 2.89 | 0.58 |
| AC | Harvard | *L. handelianus* | 34143 | Had 1-1 | 52.50 | 0.18 | 0.54 | 0.08 | 0.38 | 0.83 | 0.29 | 1.85 | 0.49 | 0.95 | 0.37 | 2.34 | 0.47 |
| AC | Harvard | *L. handelianus* | 34143 | Had 1-2 | 55.00 | 0.21 | 0.58 | 0.08 | 0.42 | 0.88 | 0.33 | 1.91 | 0.60 | 1.04 | 0.30 | 2.40 | 0.53 |
| AC | Harvard | *L. handelianus* | 64089 | Had 2-1 | 52.50 | 0.25 | 0.72 | 0.08 | 0.36 | 1.03 | 0.39 | 2.20 | 0.62 | 0.86 | 0.43 | 2.64 | 0.60 |
| AC | KIB | *L. handelianus* | 500002 | Had 3-1 | 55.00 | 0.23 | 0.65 | 0.09 | 0.41 | 0.82 | 0.37 | 2.07 | 0.62 | 1.03 | 0.58 | 2.52 | 0.59 |
| AC | KIB | *L. handelianus* | 502290 | Had 4-1 | 67.50 | 0.28 | 0.61 | 0.05 | 0.35 | 0.67 | 0.33 | 1.85 | 0.54 | 0.78 | 0.57 | 2.17 | 0.55 |
| AC | KIB | *L. handelianus* | 502290 | Had 4-2 | 65.00 | 0.26 | 0.63 | 0.07 | 0.41 | 0.72 | 0.36 | 1.89 | 0.59 | 0.95 | 0.34 | 2.30 | 0.53 |
| AC | KIB | *L. handelianus* | 502290 | Had 4-3 | 67.50 | 0.28 | 0.66 | 0.07 | 0.39 | 0.76 | 0.37 | 1.85 | 0.64 | 0.96 | 0.34 | 2.37 | 0.62 |
| AC | KIB | *L. handelianus* | 502305 | Had 5-1 | 72.50 | 0.51 | 1.05 | 0.16 | 0.68 | 1.51 | 0.61 | 2.77 | 1.05 | 1.24 | 0.76 | 3.65 | 1.05 |
| AC | KIB | *L. handelianus* | 502305 | Had 5-2 | 67.50 | 0.47 | 1.04 | 0.21 | 0.77 | 1.62 | 0.63 | 2.07 | 1.04 | 1.47 | 0.64 | 3.70 | 1.11 |
| AC | Harvard | *L. harlandii* | 367 | Har 1-1 | 55.00 | 0.48 | 0.65 | 0.13 | 0.35 | 1.18 | 0.34 | 2.75 | 0.46 | 0.89 | 0.47 | 2.77 | 0.55 |
| AC | Harvard | *L. harlandii* | 367 | Har 1-2 | 55.00 | 0.40 | 0.62 | 0.07 | 0.28 | 1.06 | 0.34 | 2.52 | 0.43 | 0.61 | 0.32 | 2.65 | 0.50 |
| AC | Harvard | *L. harlandii* | NA | Har 2-1 | 55.00 | 0.36 | 0.61 | 0.06 | 0.21 | 1.27 | 0.35 | 2.89 | 0.52 | 0.47 | 0.17 | 2.86 | 0.62 |
| AC | Harvard | *L. harlandii* | NA | Har 2-2 | 55.50 | 0.33 | 0.57 | 0.06 | 0.22 | 0.99 | 0.31 | 2.52 | 0.55 | 0.46 | 0.12 | 2.55 | 0.15 |
| AC | Harvard | *L. harlandii* | 1026 | Har 3-1 | 60.00 | 0.65 | 0.76 | 0.12 | 0.33 | 1.54 | 0.37 | 3.30 | 0.56 | 1.23 | 0.45 | 3.29 | 0.50 |
| AC | Harvard | *L. harlandii* | 1026 | Har 3-2 | 60.00 | 0.43 | 0.77 | 0.16 | 0.46 | 1.62 | 0.45 | 3.21 | 0.67 | 1.34 | 0.23 | 3.28 | 0.66 |
| AC | Harvard | *L. harlandii* | 732 | Har 4-1 | 57.50 | 0.36 | 0.68 | 0.06 | 0.36 | 1.19 | 0.38 | 2.69 | 0.47 | 0.73 | 0.21 | 2.84 | 0.62 |
| AC | Harvard | *L. harlandii* | 732 | Har 4-2 | 50.00 | 0.27 | 0.53 | 0.06 | 0.28 | 0.97 | 0.28 | 2.47 | 0.48 | 0.70 | 0.27 | 2.57 | 0.50 |
| AC | Harvard | *L. henryi* | 543 | Hen 1-1 | 70.00 | 0.30 | 0.78 | 0.05 | 0.40 | 1.22 | 0.46 | 2.54 | 0.82 | 0.85 | 0.33 | 2.90 | 0.76 |
| AC | Harvard | *L. henryi* | 543 | Hen 1-2 | 75.00 | 0.29 | 0.82 | 0.07 | 0.53 | 1.20 | 0.46 | 2.52 | 0.80 | 1.10 | 0.51 | 2.88 | 0.68 |
| AC | Harvard | *L. henryi* | 524 | Hen 2-1 | 61.00 | 0.25 | 0.64 | 0.09 | 0.37 | 0.83 | 0.34 | 2.30 | 0.64 | 0.89 | 0.48 | 2.56 | 0.57 |
| AC | Harvard | *L. henryi* | 524 | Hen 2-2 | 67.50 | 0.29 | 0.66 | 0.11 | 0.37 | 1.05 | 0.39 | 2.48 | 0.69 | 0.91 | 0.48 | 2.63 | 0.59 |
| AC | Harvard | *L. henryi* | 524 | Hen 2-3 | 57.50 | 0.22 | 0.58 | 0.06 | 0.24 | 0.86 | 0.33 | 2.25 | 0.29 | 0.60 | 0.16 | 2.45 | 0.38 |
| AC | KIB | *L. himalaicus* | 502422 | Him 1-1 | 67.00 | 0.29 | 0.74 | 0.11 | 0.56 | 0.94 | 0.42 | 2.22 | 0.69 | 1.43 | 0.56 | 2.64 | 0.58 |
| AC | KIB | *L. himalaicus* | 502422 | Him 1-2 | 55.00 | 0.27 | 0.59 | 0.05 | 0.35 | 0.87 | 0.33 | 2.20 | 0.56 | 0.74 | 0.16 | 2.42 | 0.57 |
| AC | KIB | *L. himalaicus* | 502422 | Him 1-3 | 67.50 | 0.30 | 0.68 | 0.08 | 0.43 | 1.05 | 0.38 | 2.26 | 0.59 | 0.99 | 0.33 | 2.66 | 0.49 |
| AC | KIB | *L. himalaicus* | 502403 | Him 1-4 | 57.50 | 0.15 | 0.64 | 0.04 | 0.29 | 0.95 | 0.37 | 2.14 | 0.55 | 0.66 | 0.27 | 2.56 | 0.61 |
| AC | Harvard | *L. howii* | 26878 | How 1-1 | 53.50 | 0.05 | 0.45 | 0.03 | 0.16 | 0.44 | 0.29 | 1.54 | 0.44 | 0.41 | 0.19 | 1.86 | 0.44 |
| AC | KIB | *L. hypoglaucus* | 502452 | Hyp 1-1 | 70.00 | 0.15 | 0.55 | 0.03 | 0.30 | 0.56 | 0.32 | 1.75 | 0.53 | 0.63 | 0.16 | 1.98 | 0.52 |
| AC | KIB | *L. hypoglaucus* | 502452 | Hyp 1-2 | 70.00 | 0.19 | 0.65 | 0.04 | 0.45 | 0.82 | 0.35 | 1.97 | 0.55 | 0.85 | 0.30 | 2.41 | 0.42 |
| AC | KIB | *L. hypoglaucus* | 502452 | Hyp 1-3 | 70.00 | 0.20 | 0.67 | 0.05 | 0.43 | 0.83 | 0.35 | 1.99 | 0.45 | 0.82 | 0.31 | 2.34 | 0.65 |
| AC | KIB | *L. hypoglaucus* | 502452 | Hyp 1-4 | 70.00 | 0.20 | 0.68 | 0.07 | 0.45 | 0.89 | 0.39 | 2.07 | 0.59 | 0.82 | 0.41 | 2.47 | 0.76 |
| AC | KIB | *L. hypoglaucus* | 502441 | Hyp 2-1 | 75.00 | 0.18 | 0.66 | 0.08 | 0.49 | 0.71 | 0.40 | 1.86 | 0.58 | 1.05 | 0.57 | 2.32 | 0.58 |
| AC | KIB | *L. hypoglaucus* | 502441 | Hyp 2-2 | 70.00 | 0.13 | 0.58 | 0.06 | 0.37 | 0.58 | 0.36 | 1.63 | 0.61 | 0.82 | 0.47 | 2.08 | 0.40 |
| AC | KIB | *L. hypoglaucus* | 502438 | Hyp 3-1 | 65.00 | 0.20 | 0.68 | 0.08 | 0.58 | 0.71 | 0.42 | 1.87 | 0.59 | 1.06 | 0.54 | 2.31 | 0.58 |
| AC | KIB | *L. hypoglaucus* | 502438 | Hyp 3-2 | 65.00 | 0.23 | 0.75 | 0.08 | 0.55 | 0.74 | 0.47 | 1.87 | 0.78 | 1.06 | 0.70 | 2.50 | 0.57 |
| ER | Leiden | *L. indutus* | 30/10-41 | Ind 1-1 | 82.50 | 0.79 | 1.20 | 0.78 | 0.78 | 1.11 | 0.51 | 2.76 | 1.03 | 3.00 | 0.63 | 3.26 | 0.65 |
| ER | Leiden | *L. indutus* | 30/10-41 | Ind 1-2 | 80.75 | 0.73 | 1.12 | 0.84 | 0.80 | 1.19 | 0.48 | 2.94 | 0.95 | 3.10 | 0.70 | 3.33 | 0.58 |
| ER | Leiden | *L. indutus* | P4030025 | Ind 2-1 | 80.00 | 0.75 | 1.16 | 0.81 | 0.81 | 0.65 | 0.50 | 2.59 | 1.14 | 2.62 | 0.65 | 2.93 | 0.75 |
| AC | Leiden | *L. jacobsii* | 15574 | Jac 1-1 | 52.50 | 0.20 | 0.73 | 0.15 | 0.39 | 1.28 | 0.54 | 3.00 | 0.82 | 1.20 | 0.40 | 3.61 | 0.95 |
| AC | Leiden | *L. jacobsii* | IliasPaie15574M | Jac 2-1 | 50.00 | 0.22 | 0.72 | 0.15 | 0.42 | 1.05 | 0.48 | 2.59 | 0.93 | 1.21 | 0.61 | 3.28 | 0.38 |
| AC | Leiden | *L. jacobsii* | IliasPaie15574M | Jac 2-2 | 50.25 | 0.20 | 0.73 | 0.14 | 0.40 | 0.93 | 0.53 | 2.62 | 0.98 | 1.13 | 0.62 | 3.32 | 0.71 |
| AC | Leiden | *L. jacobsii* | IliasPaie15574M | Jac 2-3 | 48.05 | 0.26 | 0.81 | 0.11 | 0.47 | 1.24 | 0.53 | 2.86 | 0.87 | 1.03 | 0.49 | 3.51 | 0.61 |
| ER | Leiden | *L. javensis* | NA | Jav 1-1 | 120.00 | 0.09 | 0.31 | 2.54 | 0.90 | 1.75 | 0.50 | 0.63 | 0.24 | 3.52 | 0.75 | 3.52 | 0.75 |
| ER | Leiden | *L. javensis* | Koorders15213 | Jav 2-1 | 115.00 | 0.08 | 0.29 | 3.81 | 1.06 | 1.83 | 0.61 | 0.62 | 0.29 | 3.95 | 0.90 | 3.95 | 0.90 |
| ER | Leiden | *L. javensis* | Koorders15213 | Jav 2-2 | 115.00 | 0.06 | 0.19 | 3.24 | 0.88 | 1.43 | 0.49 | 0.41 | 0.14 | 4.08 | 0.59 | 3.59 | 0.78 |
| ER | Leiden | *L. javensis* | Koorders15338 | Jav 3-1 | 75.00 | 0.65 | 1.19 | 1.64 | 0.75 | 1.47 | 0.59 | 2.08 | 0.59 | 3.45 | 0.83 | 3.45 | 0.83 |
| ER | Leiden | *L. javensis* | Koorders15338 | Jav 3-2 | 86.88 | 0.13 | 0.61 | 2.75 | 1.03 | 1.92 | 0.70 | 1.05 | 0.66 | 4.62 | 0.88 | 4.09 | 1.12 |
| ER | Leiden | *L. javensis* | TorreetHarms1893 | Jav 4-1 | 77.50 | 0.45 | 1.20 | 1.76 | 1.10 | 2.49 | 0.72 | 2.13 | 0.98 | 4.77 | 0.85 | 4.77 | 0.92 |
| ER | Leiden | *L. javensis* | TorreetHarms1893 | Jav 4-2 | 77.50 | 0.36 | 1.14 | 1.83 | 1.03 | 2.36 | 0.71 | 2.09 | 1.02 | 5.10 | 1.09 | 5.10 | 0.95 |
| AC | Harvard | *L. kawakamii* | 9653 | Kaw 1-1 | 82.50 | 0.64 | 0.86 | 0.13 | 0.43 | 1.32 | 0.51 | 2.82 | 0.81 | 0.96 | 0.49 | 3.11 | 0.69 |
| AC | Harvard | *L. kawakamii* | 9653 | Kaw 1-2 | 75.00 | 0.49 | 1.25 | 0.27 | 0.81 | 1.94 | 0.67 | 2.88 | 0.92 | 3.85 | 0.79 | 1.79 | 0.94 |
| AC | Harvard | *L. kawakamii* | 9653 | Kaw 1-3 | 75.00 | 0.61 | 1.02 | 0.17 | 0.50 | 1.75 | 0.63 | 2.95 | 1.03 | 1.18 | 0.50 | 3.64 | 0.86 |
| ER | Harvard | *L. konishii* | N.H.Li. 271 | Kon 1-1 | 81.50 | 0.25 | 0.70 | 0.26 | 0.47 | 0.41 | 0.26 | 1.49 | 0.55 | 1.68 | 0.34 | 1.69 | 0.43 |
| ER | Harvard | *L. konishii* | 21326 | Kon 2-1 | 76.50 | 0.29 | 0.94 | 0.83 | 0.54 | 0.68 | 0.32 | 1.69 | 0.57 | 2.35 | 0.28 | 2.35 | 0.45 |
| ER | Harvard | *L. konishii* | 21326 | Kon 2-2 | 72.50 | 0.28 | 0.80 | 0.53 | 0.47 | 0.58 | 0.28 | 1.68 | 0.63 | 2.20 | 0.39 | 2.18 | 0.43 |
| ER | Harvard | *L. konishii* | 21246 | Kon 3-1 | 75.00 | 0.39 | 0.86 | 0.66 | 0.58 | 0.60 | 0.34 | 1.98 | 0.94 | 2.30 | 0.52 | 2.30 | 0.39 |
| ER | Harvard | *L. konishii* | 21246 | Kon 3-2 | 75.00 | 0.32 | 0.85 | 0.59 | 0.56 | 0.54 | 0.32 | 1.88 | 0.74 | 2.17 | 0.48 | 2.18 | 0.37 |
| ER | Harvard | *L. konishii* | 11174 | Kon 4-1 | 60.00 | 0.35 | 0.84 | 0.46 | 0.50 | 0.69 | 0.30 | 2.11 | 0.79 | 2.31 | 0.53 | 2.28 | 0.40 |
| ER | Harvard | *L. konishii* | 11174 | Kon 4-2 | 55.00 | 0.27 | 0.76 | 0.41 | 0.46 | 0.61 | 0.27 | 1.85 | 0.63 | 2.21 | 0.31 | 2.27 | 0.32 |
| ER | Harvard | *L. konishii* | 11174 | Kon 4-3 | 57.50 | 0.35 | 0.84 | 0.47 | 0.50 | 0.67 | 0.29 | 2.00 | 0.59 | 2.30 | 0.39 | 2.33 | 0.34 |
| ER | Leiden | *L. lampadarius* | E.C.&L.B.Abbe10002 | Lam 1-1 | 60.00 | 0.51 | 1.01 | 0.89 | 0.84 | 1.31 | 0.53 | 2.67 | 0.91 | 3.71 | 0.71 | 3.71 | 0.65 |
| ER | KIB | *L. laoticus* | 502494 | Lam 2-1 | 75.00 | 0.03 | 0.31 | 0.20 | 0.71 | 1.55 | 0.40 | 0.65 | 0.32 | 2.54 | 0.65 | 3.18 | 0.55 |
| AC | Leiden | *L. lappaceus* | C.H.Cannon817 | Lam 3-1 | 47.50 | 0.11 | 0.44 | 0.02 | 0.20 | 0.57 | 0.25 | 1.61 | 0.43 | 0.43 | 0.32 | 1.97 | 0.45 |
| AC | Leiden | *L. lappaceus* | C.H.Cannon817 | Lam 3-2 | 52.51 | 0.16 | 0.56 | 0.03 | 0.26 | 0.71 | 0.31 | 1.78 | 0.59 | 0.59 | 0.33 | 2.24 | 0.59 |
| ER | Harvard | *L. lepidocarpus* | 9760 | Lep 1-1 | 110.00 | 0.16 | 0.65 | 0.73 | 0.83 | 1.74 | 0.46 | 1.02 | 0.34 | 3.31 | 0.67 | 3.42 | 0.64 |
| AC | Leiden | *L. leptogyne* | Beaman8507 | Let 1-1 | 60.00 | 0.09 | 0.52 | 0.07 | 0.26 | 0.47 | 0.31 | 1.45 | 0.42 | 0.65 | 0.24 | 1.94 | 0.43 |
| AC | Leiden | *L. leptogyne* | CuadraA.1454 | Let 2-1 | 45.00 | 0.12 | 0.51 | 0.03 | 0.26 | 0.46 | 0.31 | 1.53 | 0.43 | 0.61 | 0.35 | 2.12 | 0.41 |
| AC | Leiden | *L. leptogyne* | Elmer21636 | Let 3-1 | 47.50 | 0.08 | 0.47 | 0.02 | 0.22 | 0.35 | 0.30 | 1.43 | 0.48 | 0.55 | 0.38 | 1.97 | 0.45 |
| AC | Leiden | *L. leptogyne* | PaieS.15844 | Let 4-1 | 45.00 | 0.06 | 0.39 | 0.02 | 0.21 | 0.33 | 0.27 | 1.42 | 0.41 | 0.48 | 0.21 | 1.86 | 0.30 |
| AC | Leiden | *L. leptogyne* | WoodSan15437 | Let 5-1 | 50.00 | 0.10 | 0.50 | 0.05 | 0.30 | 0.51 | 0.30 | 1.73 | 0.50 | 0.75 | 0.37 | 2.06 | 0.40 |
| AC | KIB | *L. lindleyanus* | 786766 | Lin 1-1 | 35.00 | 0.24 | 0.46 | 0.10 | 0.27 | 0.70 | 0.23 | 2.38 | 0.41 | 0.79 | 0.33 | 2.31 | 0.38 |
| AC | Harvard | *L. litseifolius* | 43505 | Lit 1-1 | 82.50 | 0.22 | 0.68 | 0.07 | 0.44 | 0.69 | 0.43 | 2.05 | 0.59 | 1.00 | 0.46 | 2.35 | 0.51 |
| AC | Harvard | *L. litseifolius* | 43505 | Lit 1-2 | 80.00 | 0.23 | 0.75 | 0.10 | 0.49 | 0.82 | 0.41 | 2.08 | 0.67 | 1.14 | 0.44 | 2.48 | 0.64 |
| AC | Harvard | *L. litseifolius* | 43505 | Lit 1-3 | 77.50 | 0.21 | 0.70 | 0.14 | 0.55 | 0.74 | 0.41 | 2.10 | 0.55 | 1.11 | 0.38 | 2.37 | 0.61 |
| AC | Harvard | *L. litseifolius* | 128 | Lit 2-1 | 65.00 | 0.18 | 0.52 | 0.09 | 0.29 | 0.54 | 0.30 | 1.98 | 0.45 | 0.89 | 0.31 | 2.00 | 0.38 |
| AC | Harvard | *L. litseifolius* | 128 | Lit 2-2 | 61.00 | 0.22 | 0.55 | 0.12 | 0.33 | 0.57 | 0.32 | 2.03 | 0.46 | 0.80 | 0.44 | 1.99 | 0.45 |
| AC | Harvard | *L. litseifolius* | 128 | Lit 2-3 | 67.50 | 0.15 | 0.52 | 0.08 | 0.35 | 0.54 | 0.30 | 1.89 | 0.50 | 0.78 | 0.32 | 1.99 | 0.37 |
| AC | Harvard | *L. litseifolius* | 25865 | Lit 3-1 | 73.50 | 0.19 | 0.59 | 0.05 | 0.37 | 0.76 | 0.39 | 2.24 | 0.59 | 0.79 | 0.29 | 2.40 | 0.54 |
| AC | Harvard | *L. litseifolius* | 79069 | Lit 4-1 | 52.50 | 0.15 | 0.45 | 0.05 | 0.26 | 0.49 | 0.25 | 1.83 | 0.40 | 0.60 | 0.18 | 1.85 | 0.38 |
| AC | Harvard | *L. litseifolius* | 79069 | Lit 4-2 | 57.50 | 0.14 | 0.47 | 0.06 | 0.30 | 0.50 | 0.28 | 1.77 | 0.45 | 0.65 | 0.23 | 1.86 | 0.40 |
| AC | Harvard | *L. litseifolius* | 27673 | Lit 5-1 | 65.00 | 0.39 | 0.90 | 0.18 | 0.66 | 0.97 | 0.47 | 2.13 | 0.86 | 1.35 | 0.61 | 2.72 | 0.86 |
| AC | Harvard | *L. litseifolius* | 27673 | Lit 5-2 | 70.00 | 0.32 | 0.79 | 0.14 | 0.58 | 0.89 | 0.47 | 2.11 | 0.90 | 1.21 | 0.69 | 2.64 | 0.57 |
| AC | KIB | *L. litseifolius* | 503145 | Lit 6-1 | 70.00 | 0.18 | 0.53 | 0.06 | 0.30 | 0.53 | 0.30 | 1.77 | 0.46 | 0.67 | 0.42 | 1.98 | 0.48 |
| AC | KIB | *L. litseifolius* | 503145 | Lit 6-2 | 57.50 | 0.14 | 0.50 | 0.05 | 0.22 | 0.55 | 0.29 | 1.72 | 0.44 | 0.44 | 0.24 | 2.03 | 0.41 |
| AC | KIB | *L. litseifolius* | 503145 | Lit 6-3 | 67.50 | 0.16 | 0.48 | 0.05 | 0.25 | 0.50 | 0.27 | 1.68 | 0.46 | 0.57 | 0.15 | 1.90 | 0.38 |
| AC | KIB | *L. litseifolius* | 503145 | Lit 6-4 | 65.00 | 0.24 | 0.59 | 0.07 | 0.36 | 0.59 | 0.31 | 1.81 | 0.46 | 0.94 | 0.42 | 2.02 | 0.47 |
| AC | KIB | *L. litseifolius* | 503151 | Lit 7-1 | 62.50 | 0.16 | 0.59 | 0.08 | 0.37 | 0.74 | 0.38 | 2.17 | 0.45 | 0.81 | 0.36 | 2.39 | 0.42 |
| AC | KIB | *L. longanoides* | 502530 | Lon 1-1 | 65.00 | 0.11 | 0.50 | 0.03 | 0.22 | 0.49 | 0.28 | 1.56 | 0.53 | 0.52 | 0.23 | 1.94 | 0.50 |
| AC | KIB | *L. longipedicellatus* | 804684 | Lon 2-1 | 59.00 | 0.16 | 0.57 | 0.04 | 0.32 | 0.58 | 0.30 | 1.63 | 0.58 | 0.64 | 0.27 | 2.02 | 0.39 |
| AC | KIB | *L. longipedicellatus* | 804684 | Lon 2-2 | 60.00 | 0.14 | 0.53 | 0.04 | 0.29 | 0.53 | 0.28 | 1.59 | 0.44 | 0.55 | 0.28 | 1.96 | 0.50 |
| AC | KIB | *L. longipedicellatus* | 804684 | Lon 2-3 | 61.50 | 0.18 | 0.58 | 0.04 | 0.28 | 0.61 | 0.30 | 1.75 | 0.53 | 0.56 | 0.30 | 2.04 | 0.48 |
| ER | Leiden | *L. lucidus* | Brun5259 | Luc 1-1 | 60.00 | 0.24 | 0.80 | 0.71 | 0.56 | 0.66 | 0.44 | 2.68 | 0.67 | 2.78 | 0.51 | 3.02 | 0.47 |
| ER | Leiden | *L. luteus* | Anderson4563 | Lut 1-1 | 70.00 | 0.19 | 0.68 | 0.18 | 0.32 | 0.44 | 0.43 | 1.61 | 0.67 | 1.10 | 0.27 | 2.62 | 0.41 |
| AC | KIB | *L. mairei* | 692007 | Mai 1-1 | 65.00 | 0.06 | 0.70 | 0.06 | 0.29 | 1.29 | 0.42 | 2.21 | 0.74 | 0.65 | 0.29 | 2.92 | 0.63 |
| AC | KIB | *L. mairei* | 692007 | Mai 1-2 | 64.00 | 0.10 | 0.46 | 0.03 | 0.17 | 0.49 | 0.24 | 1.54 | 0.37 | 0.41 | 0.21 | 1.78 | 0.28 |
| AC | KIB | *L. mairei* | 692007 | Mai 1-3 | 65.00 | 0.13 | 0.43 | 0.04 | 0.18 | 0.49 | 0.24 | 1.66 | 0.39 | 0.39 | 0.16 | 1.80 | 0.39 |
| AC | KIB | *L. mairei* | 692007 | Mai 1-4 | 65.00 | 0.11 | 0.45 | 0.04 | 0.22 | 0.49 | 0.24 | 1.60 | 0.38 | 0.47 | 0.18 | 1.78 | 0.40 |
| AC | KIB | *L. mairei* | 502557 | Mai 2-1 | 65.00 | 0.10 | 0.45 | 0.02 | 0.19 | 0.45 | 0.25 | 1.45 | 0.34 | 0.40 | 0.32 | 1.70 | 0.43 |
| AC | KIB | *L. mairei* | 502557 | Mai 2-2 | 62.50 | 0.10 | 0.50 | 0.03 | 0.22 | 0.52 | 0.28 | 1.46 | 0.47 | 0.50 | 0.28 | 1.85 | 0.31 |
| AC | KIB | *L. mairei* | 502557 | Mai 2-3 | 52.50 | 0.07 | 0.41 | 0.02 | 0.17 | 0.38 | 0.22 | 1.25 | 0.39 | 0.42 | 0.14 | 1.60 | 0.33 |
| AC | KIB | *L. mairei* | 502565 | Mai 3-1 | 67.50 | 0.07 | 0.44 | 0.02 | 0.17 | 0.37 | 0.27 | 1.29 | 0.47 | 0.39 | 0.20 | 1.73 | 0.35 |
| AC | KIB | *L. mairei* | 502597 | Mai 4-1 | 65.00 | 0.12 | 0.49 | 0.03 | 0.25 | 0.58 | 0.28 | 0.44 | 0.47 | 0.56 | 0.31 | 1.95 | 0.44 |
| AC | KIB | *L. mairei* | 502597 | Mai 4-2 | 56.00 | 0.10 | 0.45 | 0.03 | 0.21 | 0.52 | 0.24 | 1.70 | 0.40 | 0.55 | 0.24 | 1.82 | 0.42 |
| ER | Leiden | *L. megacarpus* | L.J.Brass13522 | Meg 1-1 | 80.00 | 1.12 | 0.51 | 1.52 | 0.91 | 2.00 | 0.55 | 3.09 | 0.33 | 3.66 | 0.79 | 3.69 | 0.83 |
| AC | Leiden | *L. meijeri* | WrightS.27181 | Mei 1-1 | 55.00 | 0.16 | 0.66 | 0.06 | 0.35 | 0.56 | 0.40 | 1.74 | 0.63 | 0.81 | 0.36 | 2.50 | 0.59 |
| AC | Harvard | *L. naiadarum* | 957 | Nai 1-1 | 65.00 | 0.20 | 0.74 | 0.07 | 0.40 | 1.22 | 0.41 | 2.55 | 0.59 | 0.99 | 0.14 | 2.85 | 0.51 |
| AC | Harvard | *L. naiadarum* | 957 | Nai 1-2 | 60.00 | 0.17 | 0.60 | 0.05 | 0.32 | 0.85 | 0.32 | 2.15 | 0.54 | 0.84 | 0.22 | 2.40 | 0.46 |
| AC | Harvard | *L. naiadarum* | 73995 | Nai 2-1 | 55.00 | 0.10 | 0.55 | 0.02 | 0.20 | 0.66 | 0.32 | 1.84 | 0.48 | 0.41 | 0.15 | 2.15 | 0.48 |
| AC | Harvard | *L. naiadarum* | 73995 | Nai 2-2 | 57.50 | 0.12 | 0.55 | 0.02 | 0.20 | 0.69 | 0.32 | 1.93 | 0.52 | 0.42 | 0.16 | 2.24 | 0.51 |
| AC | Harvard | *L. naiadarum* | 73995 | Nai 2-3 | 57.50 | 0.11 | 0.49 | 0.02 | 0.23 | 0.58 | 0.28 | 1.67 | 0.34 | 0.47 | 0.24 | 2.05 | 0.46 |
| AC | Harvard | *L. naiadarum* | 7777 | Nai 3-1 | 62.50 | 0.18 | 0.61 | 0.05 | 0.36 | 0.79 | 0.37 | 2.11 | 0.56 | 0.81 | 0.33 | 2.34 | 0.58 |
| AC | KIB | *L. naiadarum* | 502641 | Nai 4-1 | 67.50 | 0.24 | 0.61 | 0.06 | 0.33 | 0.75 | 0.37 | 2.01 | 0.60 | 0.76 | 0.28 | 2.28 | 0.54 |
| AC | KIB | *L. naiadarum* | 502641 | Nai 4-2 | 70.00 | 0.16 | 0.61 | 0.08 | 0.30 | 0.79 | 0.36 | 2.09 | 0.52 | 0.67 | 0.31 | 2.44 | 0.56 |
| AC | KIB | *L. naiadarum* | 502654 | Nai 5-1 | 60.00 | 0.12 | 0.56 | 0.05 | 0.25 | 0.72 | 0.34 | 2.03 | 0.52 | 0.60 | 0.25 | 2.23 | 0.57 |
| AC | KIB | *L. naiadarum* | 502654 | Nai 5-2 | 54.00 | 0.13 | 0.53 | 0.03 | 0.20 | 0.69 | 0.30 | 1.95 | 0.49 | 0.45 | 0.16 | 2.15 | 0.55 |
| AC | Leiden | *L. nieuwenhuisii* | SAN16481 | Niu 1-1 | 42.50 | 0.42 | 0.56 | 0.02 | 0.16 | 1.49 | 0.26 | 3.27 | 0.48 | 0.39 | 0.13 | 3.58 | 0.41 |
| AC | Leiden | *L. nodosus* | Clemens29890 | Nod 1-1 | 65.00 | 0.19 | 0.77 | 0.04 | 0.38 | 1.01 | 0.49 | 2.32 | 0.67 | 0.84 | 0.43 | 2.93 | 0.55 |
| AC | Harvard | *L. oblanceolatus* | 509 | Obl 1-1 | 50.00 | 0.34 | 0.61 | 0.05 | 0.36 | 0.95 | 0.34 | 2.16 | 0.49 | 0.73 | 0.29 | 2.54 | 0.57 |
| AC | Harvard | *L. oblanceolatus* | 509 | Obl 1-2 | 47.50 | 0.34 | 0.73 | 0.07 | 0.58 | 1.29 | 0.42 | 2.68 | 0.64 | 1.12 | 0.39 | 2.93 | 0.68 |
| AC | Harvard | *L. oblanceolatus* | 196 | Obl 2-1 | 45.00 | 0.29 | 0.62 | 0.06 | 0.44 | 1.10 | 0.39 | 2.52 | 0.51 | 0.80 | 0.41 | 2.76 | 0.60 |
| AC | KIB | *L. obscurus* | 502668 | Obs 1-1 | 60.00 | 0.10 | 0.65 | 0.03 | 0.35 | 0.85 | 0.38 | 2.06 | 0.64 | 0.76 | 0.36 | 2.49 | 0.50 |
| AC | KIB | *L. obscurus* | 502668 | Obs 1-2 | 59.00 | 0.11 | 0.61 | 0.03 | 0.29 | 0.88 | 0.35 | 2.09 | 0.59 | 0.63 | 0.36 | 2.42 | 0.58 |
| AC | KIB | *L. obscurus* | 502668 | Obs 1-3 | 54.00 | 0.10 | 0.57 | 0.03 | 0.34 | 0.70 | 0.32 | 1.81 | 0.55 | 0.79 | 0.24 | 2.17 | 0.37 |
| AC | KIB | *L. obscurus* | 502668 | Obs 1-4 | 55.00 | 0.09 | 0.56 | 0.23 | 0.32 | 0.68 | 0.32 | 1.81 | 0.52 | 0.71 | 0.48 | 2.14 | 0.58 |
| ER | Harvard | *L. pachylepis* | 288 | Pac 1-1 | 100.00 | 0.72 | 2.00 | 3.02 | 1.20 | 2.59 | 1.91 | 3.36 | 2.09 | 5.24 | 1.02 | 5.40 | 1.02 |
| ER | Harvard | *L. pachylepis* | 11500 | Pac 2-1 | 100.00 | 0.59 | 0.84 | 2.28 | 1.07 | 1.99 | 0.82 | 2.85 | 0.77 | 4.60 | 1.11 | 4.86 | 1.13 |
| ER | KIB | *L. pachylepis* | 772510 | Pac 3-1 | 100.00 | 0.44 | 1.47 | 1.33 | 0.86 | 1.63 | 0.65 | 2.47 | 1.18 | 3.98 | 0.61 | 4.01 | 0.59 |
| ER | KIB | *L. pachylepis* | 772510 | Pac 3-2 | 80.00 | 0.65 | 1.58 | 1.63 | 0.91 | 2.20 | 0.73 | 3.21 | 1.22 | 4.40 | 0.89 | 4.50 | 0.88 |
| ER | KIB | *L. pachylepis* | 502696 | Pac 4-1 | 100.00 | 0.22 | 1.05 | 1.01 | 0.73 | 1.05 | 0.50 | 1.67 | 0.67 | 3.07 | 0.54 | 3.20 | 0.54 |
| ER | KIB | *L. pachyphyllus* | 502679 | Pac 5-1 | 95.00 | 0.15 | 0.50 | 1.60 | 1.04 | 2.75 | 0.67 | 0.97 | 0.58 | 4.36 | 1.04 | 4.33 | 0.95 |
| ER | KIB | *L. pachyphyllus* | 502679 | Pac 5-2 | 60.00 | 0.17 | 0.47 | 1.95 | 1.08 | 3.10 | 0.74 | 0.98 | 0.50 | 4.64 | 1.24 | 4.76 | 1.19 |
| AC | Leiden | *L. pallidus* | Koorders1476B | Pal 1-1 | 72.50 | 0.72 | 1.31 | 0.67 | 1.04 | 2.16 | 0.68 | 3.30 | 1.00 | 2.57 | 0.82 | 4.19 | 0.95 |
| AC | Leiden | *L. pallidus* | xxxx1893b | Pal 2-1 | 90.00 | 0.92 | 1.70 | 0.37 | 1.09 | 2.56 | 1.00 | 3.55 | 1.83 | 2.29 | 0.93 | 5.24 | 1.35 |
| AC | Harvard | *L. petelotii* | 2331 | Pet 1-1 | 53.50 | 0.38 | 0.75 | 0.13 | 0.50 | 1.16 | 0.42 | 2.59 | 0.63 | 1.09 | 0.56 | 2.83 | 0.59 |
| ER | Leiden | *L. platycarpus* | Koorders20222B | Pla 1-1 | 89.00 | 0.52 | 1.51 | 0.53 | 1.06 | 2.17 | 0.69 | 2.37 | 1.14 | 2.73 | 1.03 | 4.18 | 0.96 |
| ER | Leiden | *L. platycarpus* | Koorders24732B | Pla 1-2 | 95.00 | 0.51 | 1.51 | 0.42 | 1.01 | 2.38 | 0.74 | 2.76 | 1.06 | 3.00 | 1.18 | 4.41 | 0.84 |
| ER | Leiden | *L. platycarpus* | 84 | Pla 2-1 | 72.50 | 0.43 | 1.30 | 0.33 | 0.96 | 2.25 | 0.67 | 2.65 | 1.17 | 2.53 | 1.00 | 4.10 | 0.74 |
| ER | Leiden | *L. platycarpus* | 84 | Pla 2-2 | 70.00 | 0.57 | 1.30 | 0.43 | 0.91 | 2.31 | 0.69 | 2.97 | 1.02 | 2.90 | 1.34 | 4.26 | 1.15 |
| ER | Leiden | *L. platycarpus* | 84 | Pla 2-3 | 67.17 | 0.55 | 1.38 | 0.37 | 1.04 | 2.28 | 0.67 | 2.77 | 1.16 | 2.44 | 1.18 | 4.34 | 0.92 |
| AC | Harvard | *L. polystachyus* | 1365 | Pol 1-1 | 45.00 | 0.12 | 0.42 | 0.04 | 0.21 | 0.54 | 0.25 | 1.87 | 0.38 | 0.52 | 0.25 | 2.03 | 0.41 |
| AC | Harvard | *L. polystachyus* | 1365 | Pol 1-2 | 52.50 | 0.13 | 0.50 | 0.04 | 0.26 | 0.57 | 0.30 | 1.86 | 0.36 | 0.56 | 0.15 | 2.01 | 0.40 |
| AC | Harvard | *L. polystachyus* | 1365 | Pol 1-3 | 50.00 | 0.12 | 0.48 | 0.05 | 0.30 | 0.65 | 0.28 | 1.97 | 0.48 | 0.68 | 0.36 | 2.09 | 0.43 |
| AC | KIB | *L. polystachyus* | 502791 | Pol 2-1 | 52.50 | 0.14 | 0.42 | 0.04 | 0.26 | 0.43 | 0.23 | 1.64 | 0.39 | 0.56 | 0.28 | 1.73 | 0.35 |
| AC | KIB | *L. polystachyus* | 502791 | Pol 2-2 | 50.00 | 0.12 | 0.39 | 0.04 | 0.22 | 0.36 | 0.23 | 1.63 | 0.34 | 0.52 | 0.23 | 1.59 | 0.35 |
| AC | KIB | *L. polystachyus* | 502791 | Pol 2-3 | 50.00 | 0.14 | 0.41 | 0.05 | 0.22 | 0.37 | 0.23 | 1.58 | 0.35 | 0.48 | 0.25 | 1.69 | 0.31 |
| AC | KIB | *L. polystachyus* | 502791 | Pol 2-4 | 50.00 | 0.12 | 0.39 | 0.04 | 0.21 | 0.34 | 0.21 | 1.53 | 0.33 | 0.46 | 0.23 | 1.56 | 0.31 |
| AC | KIB | *L. polystachyus* | 502797 | Pol 3-1 | 57.50 | 0.15 | 0.62 | 0.05 | 0.32 | 0.75 | 0.35 | 1.95 | 0.61 | 0.73 | 0.42 | 2.26 | 0.53 |
| AC | KIB | *L. polystachyus* | 502799 | Pol 4-1 | 62.50 | 0.11 | 0.46 | 0.05 | 0.26 | 0.42 | 0.28 | 1.57 | 0.41 | 0.61 | 0.24 | 1.73 | 0.44 |
| AC | KIB | *L. polystachyus* | 502799 | Pol 4-2 | 62.50 | 0.08 | 0.48 | 0.05 | 0.28 | 0.43 | 0.29 | 1.58 | 0.49 | 0.68 | 0.34 | 1.80 | 0.43 |
| AC | KIB | *L. polystachyus* | 502799 | Pol 4-3 | 57.50 | 0.08 | 0.43 | 0.06 | 0.30 | 0.40 | 0.27 | 1.56 | 0.44 | 0.62 | 0.14 | 1.74 | 0.42 |
| AC | KIB | *L. polystachyus* | 670308 | Pol 5-1 | 60.00 | 0.16 | 0.64 | 0.07 | 0.30 | 0.84 | 0.38 | 2.11 | 0.62 | 0.66 | 0.47 | 2.55 | 0.62 |
| AC | KIB | *L. polystachyus* | 670308 | Pol 5-2 | 60.00 | 0.14 | 0.62 | 0.07 | 0.32 | 0.71 | 0.36 | 1.89 | 0.55 | 2.36 | 0.27 | 0.69 | 0.53 |
| AC | KIB | *L. polystachyus* | 670308 | Pol 5-3 | 55.00 | 0.15 | 0.56 | 0.04 | 0.27 | 0.68 | 0.33 | 1.92 | 0.54 | 0.55 | 0.37 | 2.26 | 0.40 |
| AC | KIB | *L. polystachyus* | 502831 | Pol 6-1 | 61.50 | 0.10 | 0.51 | 0.05 | 0.30 | 0.54 | 0.32 | 1.71 | 0.50 | 0.63 | 0.34 | 2.11 | 0.42 |
| AC | KIB | *L. polystachyus* | 502831 | Pol 6-2 | 62.50 | 0.09 | 0.49 | 0.05 | 0.27 | 0.42 | 0.33 | 1.61 | 0.46 | 0.73 | 0.32 | 2.44 | 0.45 |
| AC | Leiden | *L. pseudokunstleri* | Cat.#2243 | Pse 1-1 | 40.00 | 0.34 | 0.44 | 0.02 | 0.11 | 1.38 | 0.21 | 3.74 | 0.41 | 0.41 | 0.16 | 4.07 | 0.37 |
| AC | Leiden | *L. pseudokunstleri* | S.19936 | Pse 2-1 | 37.50 | 0.41 | 0.45 | 0.02 | 0.13 | 1.43 | 0.21 | 3.82 | 0.39 | 0.47 | 0.08 | 4.30 | 0.36 |
| ER | Leiden | *L. pseudomoluccus* | Koorders25864B | Psm 1-1 | 50.00 | 0.58 | 1.01 | 0.29 | 0.58 | 1.52 | 0.54 | 3.11 | 0.69 | 2.34 | 0.70 | 4.17 | 0.71 |
| ER | Leiden | *L. pseudomoluccus* | Koorders25864B | Psm 1-2 | 56.88 | 0.84 | 1.07 | 0.27 | 0.73 | 1.47 | 0.54 | 3.28 | 0.91 | 1.78 | 0.63 | 3.69 | 0.78 |
| ER | Leiden | *L. pseudomoluccus* | Th.Valeton1912.1 | Psm 2-1 | 70.00 | 0.81 | 1.26 | 0.34 | 0.94 | 2.03 | 0.76 | 3.60 | 1.12 | 1.87 | 1.19 | 4.26 | 1.09 |
| ER | Leiden | *L. pseudomoluccus* | v.Gorkum1903.1 | Psm 3-1 | 68.00 | 1.29 | 1.46 | 0.59 | 1.39 | 2.41 | 0.88 | 4.18 | 1.30 | 3.04 | 1.19 | 5.13 | 1.25 |
| AC | KIB | *L. pseudovestitus* | 804694 | Psv 1-1 | 53.50 | 0.10 | 0.44 | 0.04 | 0.23 | 0.38 | 0.25 | 1.59 | 0.44 | 0.53 | 0.28 | 1.67 | 0.29 |
| AC | KIB | *L. pseudovestitus* | 804694 | Psv 1-2 | 55.00 | 0.13 | 0.45 | 0.04 | 0.22 | 0.36 | 0.25 | 1.47 | 0.33 | 0.48 | 0.23 | 1.62 | 0.29 |
| AC | KIB | *L. pseudovestitus* | 804694 | Psv 1-3 | 50.00 | 0.10 | 0.42 | 0.04 | 0.23 | 0.33 | 0.23 | 1.46 | 0.37 | 0.49 | 0.18 | 1.64 | 0.38 |
| AC | KIB | *L. pseudovestitus* | 804694 | Psv 1-4 | 60.00 | 0.08 | 0.45 | 0.05 | 0.30 | 0.36 | 0.26 | 1.50 | 0.44 | 0.62 | 0.16 | 1.66 | 0.36 |
| ER | KIB | *L. pseudoxizangensis* | 748552 | Psx 1-1 | 105.00 | 0.17 | 0.45 | 0.12 | 0.42 | 0.39 | 0.26 | 0.92 | 0.35 | 1.01 | 0.52 | 1.72 | 0.46 |
| ER | KIB | *L. pseudoxizangensis* | 503051 | Psx 1-2 | 82.50 | 0.39 | 0.68 | 0.27 | 0.54 | 0.99 | 0.33 | 1.79 | 0.41 | 1.30 | 0.48 | 2.56 | 0.47 |
| AC | Leiden | *L. pusillus* | Burley&Lee267 | Pus 1-1 | 50.00 | 0.46 | 1.00 | 0.19 | 0.64 | 2.49 | 0.55 | 3.63 | 0.80 | 2.04 | 0.79 | 4.31 | 0.86 |
| AC | KIB | *L. rhabdostachyus* | 503058 | Rha 1-1 | 61.00 | 0.20 | 0.62 | 0.09 | 0.35 | 0.85 | 0.37 | 1.96 | 0.59 | 0.89 | 0.38 | 2.52 | 0.54 |
| AC | KIB | *L. rhabdostachyus* | 503058 | Rha 1-2 | 60.50 | 0.14 | 0.60 | 0.06 | 0.30 | 0.66 | 0.34 | 1.68 | 0.49 | 0.73 | 0.34 | 2.20 | 0.51 |
| AC | KIB | *L. rosthornii* | 503063 | Ros 1-1 | 51.50 | 0.10 | 0.51 | 0.02 | 0.24 | 0.62 | 0.29 | 1.79 | 0.47 | 0.51 | 0.25 | 2.22 | 0.42 |
| AC | KIB | *L. rosthornii* | 503063 | Ros 1-2 | 51.50 | 0.10 | 0.57 | 0.03 | 0.30 | 0.79 | 0.34 | 1.96 | 0.56 | 0.57 | 0.29 | 2.47 | 0.54 |
| AC | KIB | *L. rosthornii* | 503063 | Ros 1-3 | 50.00 | 0.07 | 0.44 | 0.01 | 0.15 | 0.55 | 0.28 | 1.73 | 0.40 | 0.29 | 0.13 | 1.97 | 0.44 |
| AC | Harvard | *L. silvicolarum* | 73001 | Sil 1-1 | 67.50 | 0.34 | 0.65 | 0.07 | 0.38 | 0.70 | 0.42 | 2.08 | 0.57 | 0.87 | 0.49 | 2.51 | 0.58 |
| AC | Harvard | *L. silvicolarum* | 73001 | Sil 1-2 | 70.00 | 0.31 | 0.65 | 0.08 | 0.44 | 0.58 | 0.37 | 1.81 | 0.51 | 0.95 | 0.37 | 2.26 | 0.58 |
| AC | Harvard | *L. silvicolarum* | 15672 | Sil 2-1 | 70.00 | 0.19 | 0.63 | 0.03 | 0.28 | 0.57 | 0.42 | 1.80 | 0.46 | 0.63 | 0.25 | 2.45 | 0.56 |
| AC | Harvard | *L. silvicolarum* | 1078 | Sil 3-1 | 70.00 | 0.29 | 0.75 | 0.08 | 0.36 | 1.05 | 0.46 | 2.22 | 0.72 | 0.67 | 0.39 | 2.85 | 0.67 |
| AC | KIB | *L. silvicolarum* | 503085 | Sil 4-1 | 77.50 | 0.28 | 0.74 | 0.10 | 0.43 | 0.74 | 0.48 | 1.90 | 0.62 | 0.91 | 0.49 | 2.58 | 0.58 |
| AC | KIB | *L. silvicolarum* | 503085 | Sil 4-2 | 77.50 | 0.25 | 0.71 | 0.12 | 0.50 | 0.80 | 0.46 | 2.05 | 0.65 | 1.04 | 0.59 | 2.66 | 0.64 |
| AC | KIB | *L. silvicolarum* | 503085 | Sil 4-3 | 75.00 | 0.27 | 0.69 | 0.09 | 0.43 | 0.72 | 0.43 | 1.91 | 0.74 | 0.95 | 0.37 | 2.48 | 0.56 |
| AC | KIB | *L. silvicolarum* | 503085 | Sil 4-4 | 72.50 | 0.27 | 0.69 | 0.09 | 0.44 | 0.75 | 0.44 | 1.92 | 0.68 | 1.03 | 0.44 | 2.53 | 0.67 |
| AC | Harvard | *L. skanianus* | 18055 | Ska 1-1 | 70.00 | 0.23 | 0.61 | 0.07 | 0.41 | 0.56 | 0.41 | 1.76 | 0.63 | 0.93 | 0.40 | 2.34 | 0.59 |
| AC | Harvard | *L. skanianus* | 18055 | Ska 1-2 | 70.00 | 0.23 | 0.59 | 0.06 | 0.26 | 0.62 | 0.38 | 1.70 | 0.65 | 0.74 | 0.18 | 2.36 | 0.53 |
| AC | Leiden | *L. sundaicus* | Abbe12068 | Sun 1-1 | 44.00 | 0.10 | 0.53 | 0.04 | 0.31 | 0.67 | 0.34 | 1.83 | 0.49 | 0.70 | 0.22 | 2.29 | 0.54 |
| AC | Leiden | *L. sundaicus* | Wood17185 | Sun 1-2 | 42.50 | 0.13 | 0.59 | 0.08 | 0.35 | 0.77 | 0.33 | 1.98 | 0.48 | 0.81 | 0.34 | 2.45 | 0.41 |
| AC | Harvard | *L. taitoensis* | 11170 | Tai 1-1 | 62.50 | 0.23 | 0.65 | 0.09 | 0.41 | 0.78 | 0.36 | 2.13 | 0.63 | 1.04 | 0.52 | 2.35 | 0.59 |
| AC | Harvard | *L. taitoensis* | 11170 | Tai 1-2 | 67.50 | 0.23 | 0.74 | 0.10 | 0.30 | 0.81 | 0.44 | 2.27 | 0.57 | 1.04 | 0.37 | 2.69 | 0.58 |
| AC | Harvard | *L. taitoensis* | 11170 | Tai 1-3 | 60.00 | 0.21 | 0.58 | 0.08 | 0.33 | 0.69 | 0.39 | 2.31 | 0.58 | 0.81 | 0.28 | 2.45 | 0.53 |
| AC | KIB | *L. taitoensis* | 804234 | Tai 2-1 | 60.00 | 0.15 | 0.51 | 0.03 | 0.26 | 0.57 | 0.28 | 1.82 | 0.38 | 0.72 | 0.27 | 1.97 | 0.41 |
| AC | KIB | *L. taitoensis* | 804234 | Tai 2-2 | 59.00 | 0.14 | 0.50 | 0.05 | 0.32 | 0.56 | 0.30 | 1.81 | 0.44 | 0.76 | 0.28 | 1.97 | 0.38 |
| AC | KIB | *L. taitoensis* | 804234 | Tai 2-3 | 56.50 | 0.13 | 0.51 | 0.05 | 0.31 | 0.54 | 0.27 | 1.84 | 0.45 | 0.68 | 0.24 | 1.96 | 0.51 |
| AC | KIB | *L. taitoensis* | 804234 | Tai 2-4 | 60.00 | 0.17 | 0.51 | 0.04 | 0.31 | 0.58 | 0.28 | 1.89 | 0.43 | 0.74 | 0.29 | 1.95 | 0.40 |
| AC | KIB | *L. touranensis* | 503187 | Tou 1-1 | 76.50 | 0.16 | 0.67 | 0.07 | 0.40 | 0.67 | 0.38 | 1.77 | 0.67 | 0.90 | 0.42 | 2.29 | 0.50 |
| AC | KIB | *L. touranensis* | 503187 | Tou 1-2 | 76.00 | 0.13 | 0.57 | 0.02 | 0.34 | 0.27 | 0.39 | 1.27 | 0.49 | 0.68 | 0.33 | 2.02 | 0.54 |
| AC | Harvard | *L. trachycarpus* | 81134 | Tra 1-1 | 77.50 | 0.12 | 0.54 | 0.04 | 0.18 | 0.68 | 0.30 | 1.71 | 0.46 | 0.45 | 0.21 | 2.12 | 0.51 |
| AC | Harvard | *L. trachycarpus* | 81134 | Tra 1-2 | 75.00 | 0.12 | 0.49 | 0.04 | 0.21 | 0.65 | 0.28 | 1.66 | 0.45 | 0.45 | 0.11 | 2.08 | 0.37 |
| AC | Harvard | *L. trachycarpus* | 81134 | Tra 1-3 | 71.00 | 0.10 | 0.44 | 0.03 | 0.18 | 0.61 | 0.25 | 1.76 | 0.45 | 0.41 | 0.21 | 2.03 | 0.44 |
| ER | Harvard | *L. truncatus* | 77964 | Tru 1-1 | 65.00 | 0.06 | 0.39 | 0.09 | 0.46 | 0.58 | 0.24 | 0.73 | 0.30 | 1.23 | 0.44 | 1.95 | 0.41 |
| ER | Harvard | *L. truncatus* | 77964 | Tru 1-2 | 67.50 | 0.09 | 0.42 | 0.10 | 0.43 | 0.60 | 0.24 | 0.79 | 0.32 | 1.16 | 0.38 | 1.97 | 0.41 |
| ER | Harvard | *L. truncatus* | 79110 | Tru 2-1 | 92.00 | 0.05 | 0.41 | 0.14 | 0.41 | 0.68 | 0.29 | 0.71 | 0.40 | 1.40 | 0.49 | 2.10 | 0.40 |
| ER | Harvard | *L. truncatus* | 79110 | Tru 2-2 | 84.00 | 0.05 | 0.35 | 0.14 | 0.47 | 0.55 | 0.26 | 0.57 | 0.33 | 1.31 | 0.47 | 1.88 | 0.42 |
| ER | Field08 | *L. truncatus* | trun2008-02 | Tru 3-1 | 82.50 | 0.03 | 0.28 | 0.16 | 0.41 | 0.53 | 0.23 | 0.43 | 0.22 | 1.44 | 0.40 | 1.85 | 0.27 |
| ER | Field08 | *L. truncatus* | trun2008-02 | Tru 3-2 | 72.50 | 0.04 | 0.29 | 0.17 | 0.37 | 0.49 | 0.22 | 0.63 | 0.24 | 1.46 | 0.42 | 1.80 | 0.30 |
| ER | Field08 | *L. truncatus* | trun2008-02 | Tru 3-3 | 70.00 | 0.03 | 0.25 | 0.13 | 0.37 | 0.51 | 0.23 | 0.56 | 0.31 | 1.46 | 0.18 | 1.84 | 0.36 |
| ER | Field08 | *L. truncatus* | trun2008-02 | Tru 3-4 | 71.00 | 0.02 | 0.22 | 0.11 | 0.36 | 0.44 | 0.21 | 0.47 | 0.18 | 1.39 | 0.35 | 1.70 | 0.38 |
| ER | Field08 | *L. truncatus* | trun2008-03 | Tru 4-1 | 65.00 | 0.03 | 0.22 | 0.09 | 0.28 | 0.32 | 0.16 | 0.51 | 0.16 | 1.07 | 0.22 | 1.48 | 0.24 |
| ER | Field08 | *L. truncatus* | trun2008-03 | Tru 4-2 | 70.00 | 0.03 | 0.26 | 0.10 | 0.33 | 0.42 | 0.19 | 0.52 | 0.26 | 1.29 | 0.30 | 1.70 | 0.32 |
| ER | Field08 | *L. truncatus* | trun2008-03 | Tru 4-3 | 70.00 | 0.03 | 0.26 | 0.14 | 0.37 | 0.51 | 0.24 | 0.53 | 0.29 | 1.43 | 0.39 | 1.84 | 0.35 |
| ER | Field08 | *L. truncatus* | trun2008-01 | Tru 5-1 | 70.00 | 0.03 | 0.28 | 0.18 | 0.44 | 0.78 | 0.29 | 0.62 | 0.18 | 1.98 | 0.50 | 2.26 | 0.54 |
| ER | Field08 | *L. truncatus* | trun2008-01 | Tru 5-2 | 70.00 | 0.05 | 0.34 | 0.16 | 0.44 | 0.71 | 0.27 | 0.71 | 0.38 | 1.77 | 0.42 | 2.18 | 0.43 |
| ER | Field08 | *L. truncatus* | trun2008-01 | Tru 5-3 | 67.50 | 0.04 | 0.28 | 0.15 | 0.39 | 0.63 | 0.24 | 0.66 | 0.18 | 1.56 | 0.42 | 2.03 | 0.38 |
| ER | KIB | *L. truncatus* | 503241 | Tru 6-1 | 67.50 | 2.24 | 0.33 | 0.14 | 0.40 | 0.51 | 0.22 | 0.68 | 0.31 | 1.31 | 0.38 | 1.85 | 0.32 |
| ER | KIB | *L. truncatus* | 503241 | Tru 6-2 | 65.00 | 0.05 | 0.31 | 0.09 | 0.37 | 0.42 | 0.22 | 0.68 | 0.21 | 1.09 | 0.35 | 1.65 | 0.33 |
| ER | KIB | *L. truncatus* | 503265 | Tru 7-1 | 80.00 | 0.06 | 0.40 | 0.14 | 0.46 | 0.62 | 0.29 | 0.83 | 0.46 | 1.46 | 0.39 | 2.02 | 0.50 |
| ER | KIB | *L. truncatus* | 743185 | Tru 8-1 | 74.00 | 0.10 | 0.45 | 0.08 | 0.45 | 0.58 | 0.26 | 1.00 | 0.30 | 1.30 | 0.42 | 1.93 | 0.44 |
| ER | KIB | *L. truncatus* | 743185 | Tru 8-2 | 72.50 | 0.08 | 0.45 | 0.08 | 0.43 | 0.67 | 0.28 | 1.01 | 0.50 | 1.22 | 0.46 | 2.06 | 0.48 |
| ER | KIB | *L. truncatus* | 743185 | Tru 8-3 | 72.50 | 0.14 | 0.51 | 0.15 | 0.58 | 0.82 | 0.32 | 1.12 | 0.50 | 1.60 | 0.46 | 2.30 | 0.52 |
| ER | Field08 | *L. truncatus* | 031HD | Tru 9-1 | 55.00 | 0.16 | 0.48 | 0.34 | 0.54 | 1.00 | 0.30 | 1.37 | 0.42 | 2.19 | 0.58 | 2.61 | 0.46 |
| ER | Field08 | *L. truncatus* | 031HD | Tru 9-2 | 55.00 | 0.12 | 0.36 | 0.36 | 0.67 | 1.20 | 0.36 | 0.93 | 0.39 | 2.23 | 0.64 | 2.83 | 0.49 |
| ER | Field08 | *L. truncatus* | 031HD | Tru 9-3 | 55.00 | 0.19 | 0.51 | 0.37 | 0.59 | 1.00 | 0.33 | 1.23 | 0.56 | 1.85 | 0.54 | 2.56 | 0.47 |
| ER | Field08 | *L. truncatus* | 012HD | Tru 10-1 | 71.00 | 0.06 | 0.35 | 0.19 | 0.47 | 0.53 | 0.26 | 0.67 | 0.27 | 1.76 | 0.38 | 1.86 | 0.43 |
| ER | Field08 | *L. truncatus* | 012HD | Tru 10-2 | 75.00 | 0.05 | 0.29 | 0.18 | 0.47 | 0.57 | 0.28 | 0.63 | 0.32 | 1.83 | 0.39 | 1.95 | 0.42 |
| ER | Field08 | *L. truncatus* | 012HD | Tru 10-3 | 75.00 | 0.09 | 0.38 | 0.16 | 0.44 | 0.57 | 0.26 | 0.84 | 0.30 | 1.90 | 0.43 | 1.96 | 0.37 |
| ER | Leiden | *L. turbinatus* | J.&M.S.Clemens29884 | Tur 1-1 | 80.00 | 0.47 | 0.96 | 4.35 | 0.77 | 1.13 | 0.25 | 1.60 | 0.90 | 4.35 | 0.33 | 4.37 | 0.33 |
| ER | Leiden | *L. turbinatus* | SAN20389 | Tur 2-1 | 75.00 | 1.08 | 1.03 | 3.57 | 1.17 | 2.42 | 0.46 | 1.87 | 0.74 | 3.76 | 0.78 | 4.41 | 0.69 |
| ER | Harvard | *L. uvariifolius* | 610 | Uva 1-1 | 75.00 | 0.29 | 0.95 | 0.84 | 0.70 | 1.05 | 0.36 | 1.83 | 0.81 | 2.47 | 0.60 | 2.59 | 0.62 |
| ER | Harvard | *L. uvariifolius* | 610 | Uva 1-2 | 77.50 | 0.58 | 1.39 | 1.44 | 0.98 | 2.04 | 0.57 | 2.21 | 1.14 | 3.59 | 0.73 | 3.77 | 0.95 |
| ER | Harvard | *L. uvariifolius* | 25829 | Uva 2-1 | 76.50 | 0.46 | 1.14 | 1.18 | 0.76 | 1.13 | 0.44 | 1.80 | 0.93 | 2.83 | 0.66 | 2.88 | 0.59 |
| ER | Harvard | *L. uvariifolius* | 25931 | Uva 3-1 | 85.00 | 0.36 | 1.01 | 0.71 | 0.81 | 1.10 | 0.41 | 1.60 | 0.74 | 2.64 | 0.54 | 2.75 | 0.50 |
| ER | Harvard | *L. uvariifolius* | 25931 | Uva 3-2 | 72.50 | 0.42 | 0.95 | 0.53 | 0.70 | 1.18 | 0.37 | 1.89 | 0.77 | 2.53 | 0.61 | 2.77 | 0.50 |
| AC | Harvard | *L. variolosus* | 11590 | Var 1-1 | 75.00 | 0.08 | 0.58 | 0.16 | 0.65 | 0.91 | 0.36 | 1.25 | 0.55 | 1.83 | 0.59 | 2.47 | 0.49 |
| AC | Harvard | *L. variolosus* | 11590 | Var 1-2 | 75.00 | 0.12 | 0.51 | 0.08 | 0.48 | 0.55 | 0.31 | 1.22 | 0.49 | 0.98 | 0.34 | 1.93 | 0.58 |
| ER | Harvard | *L. variolosus* | 72006 | Var 1-3 | 82.50 | 0.13 | 0.61 | 0.17 | 0.52 | 0.79 | 0.35 | 1.20 | 0.53 | 1.33 | 0.55 | 2.34 | 0.47 |
| ER | Harvard | *L. variolosus* | 6523 | Var 1-4 | 85.00 | 0.21 | 0.77 | 0.20 | 0.62 | 1.21 | 0.43 | 1.54 | 0.69 | 2.14 | 0.73 | 2.89 | 0.72 |
| ER | Harvard | *L. variolosus* | 6523 | Var 1-5 | 80.00 | 0.13 | 0.53 | 0.11 | 0.60 | 0.87 | 0.32 | 1.14 | 0.48 | 1.53 | 0.47 | 2.37 | 0.55 |
| AC | Harvard | *L. vestitus* | 24549 | Ves 1-1 | 52.50 | 0.12 | 0.48 | 0.05 | 0.22 | 0.42 | 0.26 | 1.55 | 0.47 | 0.62 | 0.29 | 1.77 | 0.34 |
| AC | Harvard | *L. vestitus* | 24549 | Ves 1-2 | 49.00 | 0.13 | 0.47 | 0.06 | 0.25 | 0.39 | 0.26 | 1.51 | 0.37 | 0.67 | 0.34 | 1.78 | 0.38 |
| AC | Harvard | *L. vestitus* | 24549 | Ves 1-3 | 55.00 | 0.14 | 0.53 | 0.08 | 0.30 | 0.46 | 0.29 | 1.67 | 0.47 | 0.77 | 0.29 | 1.85 | 0.51 |
| AC | Harvard | *L. vestitus* | 24549 | Ves 1-4 | 53.00 | 0.11 | 0.42 | 0.06 | 0.24 | 0.39 | 0.25 | 1.64 | 0.43 | 0.66 | 0.40 | 1.67 | 0.27 |
| ER | Field08 | *L. xylocarpus* | XY-DNA-06 | Xyl 1-1 | 75.00 | 0.15 | 0.66 | 0.43 | 0.90 | 1.09 | 0.52 | 1.33 | 0.71 | 3.44 | 0.79 | 3.78 | 0.71 |
| ER | Field08 | *L. xylocarpus* | 033HD | Xyl 2-1 | 73.00 | 0.36 | 0.78 | 0.44 | 0.85 | 1.94 | 0.48 | 1.60 | 0.95 | 2.75 | 0.81 | 3.66 | 0.69 |
| ER | Field08 | *L. xylocarpus* | XY-DNA-10 | Xyl 3-1 | 66.00 | 0.14 | 0.47 | 0.56 | 0.63 | 1.05 | 0.33 | 1.14 | 0.51 | 2.56 | 0.55 | 2.76 | 0.51 |
| ER | Field08 | *L. xylocarpus* | XY-DNA-10 | Xyl 3-2 | 70.00 | 0.08 | 0.46 | 0.43 | 0.77 | 1.44 | 0.44 | 0.85 | 0.46 | 2.78 | 0.76 | 3.07 | 0.76 |
| ER | Field08 | *L. xylocarpus* | XY-DNA-05 | Xyl 4-1 | 75.00 | 0.16 | 0.56 | 0.45 | 0.55 | 0.86 | 0.32 | 1.24 | 0.40 | 2.26 | 0.48 | 2.39 | 0.49 |
| ER | Field08 | *L. xylocarpus* | XY-DNA-05 | Xyl 4-2 | 75.00 | 0.09 | 0.45 | 0.52 | 0.58 | 0.89 | 0.34 | 0.90 | 0.39 | 2.29 | 0.49 | 2.45 | 0.52 |
| ER | Field08 | *L. xylocarpus* | XY-DNA-07 | Xyl 5-1 | 75.00 | 0.19 | 0.73 | 0.46 | 0.78 | 1.39 | 0.45 | 1.57 | 0.63 | 3.00 | 0.81 | 3.04 | 0.52 |
| ER | Field08 | *L. xylocarpus* | XY-DNA-07 | Xyl 5-2 | 75.00 | 0.19 | 0.69 | 0.47 | 0.77 | 1.42 | 0.45 | 1.44 | 0.63 | 2.87 | 0.62 | 3.08 | 0.70 |
| ER | Field08 | *L. xylocarpus* | XY-DNA-06 | Xyl 6-1 | 70.00 | 0.21 | 0.63 | 0.53 | 0.78 | 1.33 | 0.40 | 1.40 | 0.70 | 2.68 | 0.68 | 2.95 | 0.60 |
| ER | Field08 | *L. xylocarpus* | XY-DNA-06 | Xyl 6-2 | 69.00 | 0.36 | 0.77 | 0.37 | 0.66 | 1.36 | 0.40 | 1.72 | 0.67 | 1.89 | 0.64 | 3.00 | 0.45 |
| ER | Field08 | *L. xylocarpus* | XY-DNA-06 | Xyl 6-3 | 65.00 | 0.23 | 0.66 | 0.71 | 0.73 | 1.25 | 0.37 | 1.52 | 0.59 | 2.74 | 0.62 | 2.90 | 0.48 |
| ER | Field08 | *L. xylocarpus* | XY-DNA-05 | Xyl 7-1 | 80.00 | 0.15 | 0.61 | 0.52 | 0.84 | 1.46 | 0.47 | 1.44 | 0.58 | 2.87 | 0.71 | 3.12 | 0.79 |
| ER | Field08 | *L. xylocarpus* | XY-DNA-05 | Xyl 7-2 | 72.50 | 0.16 | 0.50 | 0.35 | 0.64 | 1.00 | 0.36 | 1.03 | 0.49 | 2.12 | 0.64 | 2.61 | 0.52 |
| ER | Field08 | *L. xylocarpus* | AL-XY-01 | Xyl 8-1 | 77.50 | 0.11 | 0.49 | 0.46 | 0.76 | 1.57 | 0.44 | 1.01 | 0.54 | 3.08 | 0.73 | 3.17 | 0.79 |
| ER | Field08 | *L. xylocarpus* | AL-XY-01 | Xyl 8-2 | 77.50 | 0.18 | 0.70 | 0.48 | 0.77 | 1.49 | 0.45 | 1.52 | 0.71 | 2.87 | 0.62 | 3.14 | 0.64 |
| ER | Field08 | *L. xylocarpus* | AL-XY-01 | Xyl 8-3 | 70.00 | 0.16 | 0.66 | 0.35 | 0.69 | 1.64 | 0.43 | 1.80 | 0.58 | 3.19 | 0.72 | 3.27 | 0.63 |
| ER | Field08 | *L. xylocarpus* | 018HD | Xyl 9-1 | 64.00 | 0.21 | 0.65 | 0.50 | 0.66 | 1.04 | 0.32 | 1.44 | 0.61 | 2.57 | 0.57 | 2.73 | 0.58 |
| ER | Field08 | *L. xylocarpus* | 018HD | Xyl 9-2 | 67.50 | 0.09 | 0.54 | 0.40 | 0.67 | 1.22 | 0.38 | 1.23 | 0.58 | 2.75 | 0.58 | 2.86 | 0.59 |
| ER | Field08 | *L. xylocarpus* | 018HD | Xyl 9-3 | 75.00 | 0.19 | 0.64 | 0.49 | 0.64 | 0.90 | 0.34 | 1.30 | 0.55 | 2.35 | 0.53 | 2.54 | 0.47 |
| ER | Field08 | *L. xylocarpus* | 027HD | Xyl 10-1 | 75.00 | 0.19 | 0.72 | 0.42 | 0.75 | 1.39 | 0.42 | 1.38 | 0.67 | 2.81 | 0.69 | 3.04 | 0.64 |
| ER | Field08 | *L. xylocarpus* | 027HD | Xyl 10-2 | 71.50 | 0.22 | 0.75 | 0.31 | 0.68 | 1.43 | 0.42 | 1.64 | 0.76 | 2.89 | 0.63 | 3.02 | 0.58 |
| ER | Field08 | *L. xylocarpus* | 027HD | Xyl 10-3 | 75.00 | 0.19 | 0.81 | 0.41 | 0.81 | 1.52 | 0.47 | 1.46 | 0.79 | 3.02 | 0.67 | 3.16 | 0.74 |
| ER | Field08 | *L. xylocarpus* | 031HD | Xyl 11-1 | 70.00 | 0.19 | 0.75 | 0.35 | 0.72 | 1.64 | 0.47 | 1.58 | 0.59 | 3.14 | 0.75 | 3.29 | 0.92 |
| ER | Field08 | *L. xylocarpus* | 031HD | Xyl 11-2 | 67.50 | 0.16 | 0.66 | 0.33 | 0.66 | 1.20 | 0.39 | 1.52 | 0.52 | 2.35 | 0.65 | 2.88 | 0.64 |
| ER | Field08 | *L. xylocarpus* | 030HD | Xyl 12-1 | 67.50 | 0.25 | 0.61 | 0.64 | 0.76 | 1.39 | 0.40 | 1.40 | 0.77 | 3.01 | 0.51 | 3.12 | 0.57 |
| ER | Field08 | *L. xylocarpus* | 030HD | Xyl 12-2 | 67.50 | 0.11 | 0.48 | 0.39 | 0.71 | 1.49 | 0.41 | 0.99 | 0.59 | 3.05 | 0.57 | 3.09 | 0.57 |
| ER | Field08 | *L. xylocarpus* | 030HD | Xyl 12-3 | 65.00 | 0.21 | 0.66 | 0.43 | 0.75 | 1.57 | 0.44 | 1.48 | 0.82 | 3.08 | 0.62 | 3.21 | 0.77 |
| ER | Field08 | *L. xylocarpus* | 035HD | Xyl 13-1 | 70.00 | 0.20 | 0.67 | 0.48 | 0.87 | 1.74 | 0.49 | 1.72 | 0.82 | 3.30 | 0.79 | 3.40 | 0.78 |
| ER | Field08 | *L. xylocarpus* | 035HD | Xyl 13-2 | 72.50 | 0.18 | 0.72 | 0.76 | 0.91 | 1.73 | 0.49 | 1.53 | 0.68 | 3.27 | 0.73 | 3.37 | 0.64 |
| ER | Field08 | *L. xylocarpus* | 035HD | Xyl 13-3 | 70.00 | 0.28 | 0.79 | 0.61 | 0.95 | 2.24 | 0.55 | 1.83 | 0.73 | 3.75 | 0.79 | 3.86 | 0.77 |
| ER | Field08 | *L. xylocarpus* | 036HD | Xyl 14-1 | 65.00 | 0.14 | 0.70 | 0.49 | 0.84 | 1.68 | 0.48 | 1.36 | 0.79 | 3.10 | 0.70 | 3.37 | 0.42 |
| ER | Field08 | *L. xylocarpus* | 036HD | Xyl 14-2 | 67.50 | 0.20 | 0.70 | 0.43 | 0.85 | 2.10 | 0.51 | 1.82 | 0.80 | 3.60 | 0.83 | 3.75 | 0.83 |
| ER | Field08 | *L. xylocarpus* | 036HD | Xyl 14-3 | 75.00 | 0.18 | 0.72 | 0.52 | 0.87 | 2.25 | 0.55 | 1.49 | 0.88 | 3.73 | 0.86 | 3.88 | 0.87 |
| ER | Field08 | *L. xylocarpus* | 033HD | Xyl 15-1 | 74.00 | 0.13 | 0.59 | 0.52 | 0.85 | 1.77 | 0.47 | 1.39 | 0.47 | 3.24 | 0.72 | 3.40 | 0.72 |
| ER | Field08 | *L. xylocarpus* | 033HD | Xyl 16-1 | 72.50 | 0.08 | 0.42 | 0.44 | 0.69 | 1.35 | 0.41 | 1.00 | 0.41 | 2.90 | 0.69 | 3.02 | 0.61 |
| ER | Field08 | *L. xylocarpus* | 037HD | Xyl 17-1 | 75.00 | 0.19 | 0.61 | 0.65 | 0.91 | 1.80 | 0.50 | 1.30 | 0.73 | 3.43 | 0.75 | 3.50 | 0.80 |
| ER | Field08 | *L. xylocarpus* | 037HD | Xyl 17-2 | 77.50 | 0.17 | 0.69 | 0.40 | 0.84 | 1.75 | 0.51 | 1.57 | 0.68 | 3.20 | 0.78 | 3.39 | 0.70 |
| ER | Field08 | *L. xylocarpus* | 037HD | Xyl 17-3 | 72.50 | 0.13 | 0.59 | 0.68 | 0.89 | 1.89 | 0.53 | 1.25 | 0.44 | 3.39 | 0.72 | 3.53 | 0.73 |
| ER | Field08 | *L. xylocarpus* | 029HD | Xyl 18-1 | 77.50 | 0.15 | 0.71 | 0.37 | 0.85 | 1.70 | 0.51 | 1.55 | 0.77 | 2.72 | 0.73 | 3.37 | 0.72 |
| ER | Field08 | *L. xylocarpus* | 029HD | Xyl 18-2 | 75.00 | 0.17 | 0.64 | 0.33 | 0.76 | 1.33 | 0.45 | 1.50 | 0.64 | 2.55 | 0.73 | 2.97 | 0.75 |
| ER | Field08 | *L. xylocarpus* | 028HD | Xyl 19-1 | 70.00 | 0.08 | 0.47 | 0.26 | 0.63 | 0.97 | 0.36 | 1.01 | 0.52 | 2.48 | 0.56 | 2.54 | 0.55 |
| ER | Field08 | *L. xylocarpus* | 028HD | Xyl 19-1 | 70.00 | 0.07 | 0.40 | 0.34 | 0.59 | 0.93 | 0.34 | 0.81 | 0.27 | 2.42 | 0.50 | 2.45 | 0.47 |

Each specific collection number on the specimen was recognized as one individual.
