# Supplementary material for: Associated morphometric and geospatial differentiation among 98 species of stone oaks (Lithocarpus)
Source: PLoS One. 2018 Jun 26;13(6):e0199538. doi: 10.1371/journal.pone.0199538 (PMC6019760; doi:10.1371/journal.pone.0199538)
Supplement: S2 Table — (DOCX) [file pone.0199538.s008.docx]

| **Fruit type** | **Species** | **Individual number** | **Total fruit number** |
| --- | --- | --- | --- |
| AC | *L. craibianus* | 11 | 38 |
| AC | *L. dealbatus* | 10 | 23 |
| AC | *L. elegans* | 11 | 19 |
| AC | *L. fenestratus* | 7 | 19 |
| AC | *L. hancei* | 27 | 75 |
| AC | *L. litseifolius* | 7 | 16 |
| AC | *L. polystarchyus* | 6 | 16 |
| ER | *L. cleistocarpus* | 4 | 7 |
| ER | *L. javensis* | 4 | 7 |
| ER | *L. truncatus* | 10 | 26 |
| ER | *L. xylocarpus* | 19 | 42 |

Each specific collection number on the specimen was recognized as one individual.
