# Supplementary material for: Associated morphometric and geospatial differentiation among 98 species of stone oaks (Lithocarpus)
Source: PLoS One. 2018 Jun 26;13(6):e0199538. doi: 10.1371/journal.pone.0199538 (PMC6019760; doi:10.1371/journal.pone.0199538)
Supplement: S3 Table — (DOCX) [file pone.0199538.s009.docx]

| Fruit type | Species | Source | Distribution range | Sourthern  most  latitude  (degree) | Northern  most  latitude  (degree) | Latitude  median  (degree) | Elevation minimum  (m) | Elevation maximum  (m) | Elevation median  (m) |
| --- | --- | --- | --- | --- | --- | --- | --- | --- | --- |
| ER | *L. amygdalifolius* | Flora of China | S Fujian, Guangdong, S Guangxi, Hainan, C to S Taiwan [Vietnam] | -20.0174 | -26.1008 | -23.0591 | 500 | 2300 | 1400 |
| AC | *L. bacgiangensis* | Flora of China | Guangxi, Hainan, SE Yunnan [N Vietnam] | -14.0583 | -25.0458 | -19.5521 | 200 | 1700 | 950 |
| ER | *L. balansae* | Flora of China | SE Yunnan [N Laos, Vietnam] | -14.0583 | -25.0458 | -19.5521 | 400 | 1900 | 1150 |
| AC | *L. bancanus* | Flora Malsiana | Malesia: Sumatra (scattered in the main- land), Banka and Belitung Is., Malay Peninsula (Pahang, Johore), Borneo (scattered in Sarawak, Brunei, Kalimantan) | 2.8709 | -4.2105 | -0.6698 | 0 | 250 | 125 |
| ER | *L. beccarianus* | Flora Malsiana | Malesia: Borneo (Sarawak, Sabah, Kalimantan, scattered) | -0.9619 | -5.9788 | -3.4704 | 0 | 1500 | 750 |
| AC | *L. bennettii* | Flora Malsiana | Malesia: Sumatra (scattered), Banka (common), Malay Peninsula (Kelantan, Negri Sembilan, frequent in Johore; Singapore), Borneo (scattered, mainly N. of the equator). | 0.5897 | -4.2105 | -1.8104 | 0 | 1000 | 500 |
| AC | *L. blumeanus* | Flora Malsiana | Malesia: Borneo (scattered in Sarawak, E. Kalimantan, and Sabah) | -1.5533 | -5.9788 | -3.7661 | 0 | 1650 | 825 |
| AC | *L. brachystachyus* | Flora of China | SW Guangdong, Hainan (Changjiang Xian) | -20.0174 | -23.1322 | -21.5748 | 800 | 1000 | 900 |
| AC | *L. brevicaudatus* | Flora of China | Anhui, Fujian, Guangdong, Guangxi, Guizhou, Hainan, Hubei, Hunan, Jiangxi, Sichuan, Taiwan, Zhejiang | -20.0174 | -31.8611 | -25.9393 | 300 | 1900 | 1100 |
| AC | *L. calolepis* | Flora of China | SE Yunnan (Xichou Xian) | -25.0458 | -25.0458 | -25.0458 | 1000 | 1800 | 1400 |
| AC | *L. calophyllus* | Flora of China | SW Fujian, SW Guangdong, Guangxi, S Guizhou, S Hunan, SW Jiangxi | -22.8155 | -28.6757 | -25.7456 | 500 | 1200 | 850 |
| AC | *L. carolinae* | Flora of China | S to SE Yunnan | -25.0458 | -25.0458 | -25.0458 | 1500 | 2000 | 1750 |
| AC | *L. caudatilimbus* | Flora of China | SW Guangdong (Yangchun Xian), Hainan | -20.0174 | -23.1322 | -21.5748 | 700 | 700 | 700 |
| AC | *L. celebicus* | Flora Malsiana | Malesia: Philippines, Celebes (scattered), Moluccas, New Guinea (also including Goode- nough and Fergusson Is.) | 6.315 | -12.8797 | -3.2824 | 20 | 1200 | 610 |
| AC | *L. chrysocomus* | Flora of China | N Guangdong, NE Guangxi, S Hunan (Yizhang Xian) | -22.8155 | -28.1124 | -25.4640 | 600 | 1400 | 1000 |
| ER | *L. cleistocarpus* | Flora of China | Anhui, Fujian, N Guizhou, W Hubei, Hunan, Jiangxi, S Shaanxi, Sichuan, NE Yunnan, Zhejiang | -25.0458 | -31.8611 | -28.4535 | 1000 | 2400 | 1700 |
| AC | *L. confertus* | Flora Malsiana | Malesia: Borneo (common on MT Kinabalu) | -0.9619 | -5.9804 | -3.4712 | 900 | 1800 | 1350 |
| AC | *L. confinis* | Flora of China | W Guizhou, C to E Yunnan | -25.0458 | -26.5992 | -25.8225 | 1500 | 2400 | 1950 |
| AC | *L. conocarpus* | Flora Malsiana | Malesia: Sumatra (rare, scattered), Ma- lay Peninsula (rare, scattered), Java (W. parts, rare), Borneo (common, especially in Sabah) | 0.5897 | -4.2105 | -1.8104 | 0 | 1800 | 900 |
| ER | *L. corneus* | Flora of China | S Fujian, Guangdong, Guangxi, S Guizhou, Hainan, S Hunan, Taiwan, EC and SE Yunnan [NE Vietnam] | -20.0174 | -26.5992 | -23.3083 | 0 | 1000 | 500 |
| AC | *L. craibianus* | Flora of China | SW Sichuan, S to SW Yunnan [Laos, N Thailand] | -15.87 | -30.6512 | -23.2606 | 1500 | 2700 | 2100 |
| AC | *L. crassinervius* | Flora Malsiana | Malesia: Java (W. and Central parts, rare) | -4.2105 | -4.2105 | -4.2105 | 200 | 2000 | 1100 |
| AC | *L. cryptocarpus* | Flora of China | Yunnan (Hekou Yaozu Zizhixian) [C to NE Vietnam] | -14.0583 | -25.0458 | -19.5521 | 0 | 500 | 250 |
| AC | *L. cyclophorus* | Flora Malsiana | Peninsular Siam (Betong at 5°45'N), in Malesia: Sumatra (scattered), Malay Peninsula (Perak, Selangor, Malacca; also Penang and Singapore) | 0.5897 | -4.2105 | -1.8104 | 500 | 1500 | 1000 |
| ER | *L. damiaoshanicus* | Flora of China | Guangxi (Damiao Shan) | -22.8155 | -22.8155 | -22.8155 | 1500 | 1900 | 1700 |
| AC | *L. dasystachyus* | Flora Malsiana | Malesia: Borneo (scattered in all parts). | -0.9619 | -0.9619 | -0.9619 | 0 | 750 | 375 |
| AC | *L. dealbatus* | Flora of China | Gui- zhou, SW Sichuan, SE Xizang, Yunnan [Bhutan, NE India, N Laos, NE Myanmar, N Thailand, Vietnam] | -14.0583 | -29.6475 | -21.8529 | 1000 | 2800 | 1900 |
| AC | *L. echinotholus* | Flora of China | SE Yunnan [N Vietnam] | -14.0583 | -25.0458 | -19.5521 | 200 | 1200 | 700 |
| AC | *L. edulis* | Flora of Japan | C. & S. Japan to Nansei-shoto | -36.2048 | -36.2048 | -36.2048 | 0 | 1000 | 500 |
| AC | *L. elegans* | Flora of China, Flora Malsiana | S to SW Yunnan [Bhutan, NE India, N Laos, Nepal, NE Myanmar, Sikkim, N Thailand].Distr. India (Bhutan, Nepal), E. Pakistan (Chittagong), Burma, Indo-China; in Malesia: Sumatra, Malay Peninsula, Java, Borneo (common), Celebes (rare) | 2.8709 | -28.3949 | -12.7620 | 0 | 2400 | 1200 |
| AC | *L. elmerrillii* | Flora of China | Hainan (Baoting Xian) | -20.0174 | -20.0174 | -20.0174 | 500 | 800 | 650 |
| AC | *L. ewyckii* | Flora Malsiana | Malesia: Sumatra (most parts), Malay Peninsula (Perak, Pahang, Selangor, Malacca, Jo- hore; Singapore), Borneo (common in all parts, also Nunukan I.) | 0.5897 | -4.2105 | -1.8104 | 0 | 1800 | 900 |
| AC | *L. farinulentus* | Flora of China | S Yunnan [Cambodia, Thailand, Vietnam] | -14.0583 | -25.0458 | -19.5521 | 0 | 1000 | 500 |
| AC | *L. fenestratus* | Flora of China | C to S Guangdong, SW Guangxi, Hainan, SE Xizang (Mêdog Xian), Yunnan [Bhutan, NE India, Laos, NE Myanmar, Sikkim, N Thailand, NE Vietnam] | -14.0583 | -29.6475 | -21.8529 | 0 | 1700 | 850 |
| AC | *L. ferrugineus* | Flora Malsiana | Malesia: Borneo (Sarawak; SW. Kali- mantan; Sabah: Beaufort Hill, rare) | -0.9619 | -5.9788 | -3.4704 | 0 | 500 | 250 |
| AC | *L. fohaiensis* | Flora of China | S Yunnan | -25.0458 | -25.0458 | -25.0458 | 600 | 1500 | 1050 |
| ER | *L. fordianus* | Flora of China | SW Guizhou, S Yunnan [Vietnam] | -25.0458 | -26.5992 | -25.8225 | 700 | 1500 | 1100 |
| AC | *L. formosanus* | Flora of China | S Taiwan | -23.6978 | -23.6978 | -23.6978 | 100 | 500 | 300 |
| AC | *L. glaber* | Flora of China | Anhui, Fujian, Guangdong, Guangxi, Guizhou, Henan, Hubei, Hunan, Jiangsu, Jiangxi, Taiwan, Zhejiang [Japan] | -23.6978 | -36.2048 | -29.9513 | 0 | 1500 | 750 |
| AC | *L. glutinosus* | Flora Malsiana | Malesia: Philippines (Mindanao), Celebes (Northern Peninsula and Central part) | -3.6121 | -12.8797 | -8.2459 | 0 | 900 | 450 |
| AC | *L. gracilis* | Flora Malsiana | Malesia: S. Sumatra (Palembang and vicinity, Simalur I.), Malay Peninsula (Perak, Pahang, Selangor, Negri Sembilan; Singapore), Borneo | 0.5897 | -4.2105 | -1.8104 | 0 | 1500 | 750 |
| AC | *L. hancei* | Flora of China | Fujian, Guangdong, Guangxi, Guizhou, Hainan, Hubei, Hunan, Jiangxi, Sichuan, Taiwan, Yunnan, Zhejiang | -20.0174 | -30.5466 | -25.2820 | 0 | 2600 | 1300 |
| AC | *L. handelianus* | Flora of China | Hainan | -20.0174 | -20.0174 | -20.0174 | 400 | 1000 | 700 |
| AC | *L. harlandii* | Flora of China | Fujian, Guangdong, S Guangxi, Hainan, Hunan, S Jiangxi, Taiwan, Zhejiang | -22.8155 | -30.2674 | -26.5415 | 400 | 700 | 550 |
| AC | *L. henryi* | Flora of China | Anhui, NE Guizhou, W Hubei, W Hunan, Jiangsu, Jiangxi, S Shaanxi, E Sichuan | -26.5992 | -34.2655 | -30.4324 | 1400 | 2100 | 1750 |
| AC | *L. himalaicus* | Flora of China | Xizang Motuo, India, NE Burma | -21.9162 | -29.6475 | -25.7819 | 2000 | 2400 | 2200 |
| AC | *L. howii* | Flora of China | SW Guangdong (Yangchun Xian), Hainan | -20.0174 | -23.1322 | -21.5748 | 1000 | 1400 | 1200 |
| AC | *L. hypoglaucus* | Flora of China | SW Sichuan, NW Yunnan | -25.0458 | -30.6512 | -27.8485 | 1700 | 3000 | 2350 |
| AC | *L. indutus* | Flora Malsiana | Malesia: Java (W. Java, rather common, eastwards to Mt Slamet, 109° 15' E) | -4.2105 | -4.2105 | -4.2105 | 0 | 1800 | 900 |
| AC | *L. jacobsii* | Flora Malsiana | Malesia: Borneo (Sarawak, Sabah, rare). | -1.5533 | -5.9788 | -3.7661 | 0 | 500 | 250 |
| ER | *L. javensis* | Flora Malsiana | Malesia: Sumatra (various localities), also Singkep I., Malay Peninsula (rare), Java (rather common in the western parts, eastwards to Mt Ungaran in Central Java) | 0.5897 | -4.2105 | -1.8104 | 0 | 1800 | 900 |
| AC | *L. kawakamii* | Flora of China | Taiwan | -23.6978 | -23.6978 | -23.6978 | 700 | 2900 | 1800 |
| AC | *L. konishii* | Flora of China | E Hainan, C to S Taiwan | -20.0174 | -23.6978 | -21.8576 | 300 | 1600 | 950 |
| ER | *L. lampadarius* | Flora Malsiana | Malesia: Malay Peninsula (Perak, Pahang, Trengganu), Borneo (Sabah: Mt Kinabalu) | -0.9619 | -5.9804 | -3.4712 | 900 | 2000 | 1450 |
| ER | *L. laoticus* | Flora of China | SE Yunnan [N Laos, Vietnam] | -14.0583 | -25.0458 | -19.5521 | 1500 | 2200 | 1850 |
| ER | *L. lepidocarpus* | Flora of China | C to S Taiwan. This species has been reported from Vietnam | -23.6978 | -23.6978 | -23.6978 | 300 | 2800 | 1550 |
| AC | *L. leptogyne* | Flora Malsiana | Malesia: Sumatra (rare), Malay Penin- sula (scattered), Borneo (rather common in Sara-wak and Sabah) | -0.9619 | -5.9788 | -3.4704 | 0 | 1500 | 750 |
| AC | *L. lindleyanus* | GBIF | Myanmar, Thailand, Vietnam | -14.0583 | -21.9162 | -17.9873 | 523 | 1289 | 906 |
| AC | *L. litseifolius* | Flora of China | Fujian,Guangdong, Guangxi, Guizhou, Hainan, Hubei, Hunan, Jiangxi,Sichuan, Yunnan, Zhejiang [Laos, NE Myanmar, N Vietnam] | -14.0583 | -30.6512 | -22.3548 | 500 | 2500 | 1500 |
| AC | *L. longanoides* | Flora of China | Guangdong, Guangxi, SE Yunnan | -22.8155 | -25.0458 | -23.9307 | 500 | 1200 | 850 |
| AC | *L. longipedicellatus* | Flora of China | W Guangxi, Hainan, SE Yunnan [N Vietnam] | -14.0583 | -25.0458 | -19.5521 | 0 | 1200 | 600 |
| AC | *L. lucidus* | Flora Malsiana | Malesia: Sumatra (Asahan, Langkat, Indragiri, and Riouw), Malay Peninsula (common, also Singapore and Penang), Borneo | 0.5897 | -4.2105 | -1.8104 | 0 | 1600 | 800 |
| AC | *L. luteus Soepadmo* | Flora Malsiana | Malesia: Borneo (Mt Mulu, Mt Murut, Kapit, Sarawak; Mt Kemul, Kalimantan; Mt Ki nabalu) | -0.9619 | -4.0458 | -2.5039 | 1000 | 1800 | 1400 |
| AC | *L. mairei* | Flora of China | C to N Yunnan | -25.0458 | -25.0458 | -25.0458 | 1500 | 2500 | 2000 |
| ER | *L. megacarpus* | Flora Malsiana | Malesia: New Guinea (rather rare in the western part, more common in the eastern part) | 6.315 | 6.315 | 6.3150 | 1200 | 1900 | 1550 |
| AC | *L. meijeri* | Flora Malsiana | Malesia: Borneo (Sarawak, Brunei, Sabah) | -1.5533 | -5.9788 | -3.7661 | 0 | 1000 | 500 |
| AC | *L. naiadarum* | Flora of China | Hainan | -20.0174 | -20.0174 | -20.0174 | 0 | 0 | 0 |
| AC | *L. nieuwenhuisii* | Flora Malsiana | Malesia: Borneo (Sarawak, Kalimantan, Sabah, Brunei), also Nunukan Is.; Philippines (Mindanao, Basilan, rare) | -1.5533 | -12.8797 | -7.2165 | 0 | 500 | 250 |
| AC | *L. nodosus* | Flora Malsiana | Malesia: Borneo (Sarawak: Mts Mulu and Poi; Sabah: Mt Kinabalu) | -1.5533 | -5.9804 | -3.7669 | 900 | 2400 | 1650 |
| AC | *L. oblanceolatus* | Flora of China | W Sichuan (Emei Shan) | -30.6512 | -30.6512 | -30.6512 | 2000 | 2000 | 2000 |
| AC | *L. obscurus* | Flora of China | SE Xizang (Mêdog Xian), W Yunnan | -25.0458 | -29.6475 | -27.3467 | 1500 | 2500 | 2000 |
| ER | *L. pachylepis* | Flora of China | W Guangxi, SE Yunnan [N Vietnam] | -14.0583 | -25.0458 | -19.5521 | 900 | 1800 | 1350 |
| ER | *L. pachyphyllus* | Flora of China | SE Xizang, SW Yunnan [Bhutan, NE India, NE Myanmar, Nepal, Sikkim] | -21.9162 | -29.6475 | -25.7819 | 800 | 3200 | 2000 |
| AC | *L. pallidus* | Flora Malsiana | Malesia: S. Sumatra (Forbes, fr., 1881, from Mt Dempo), Java (rather common in the western parts, eastwards to Mts Slamet and Wilis) | 0.5897 | -4.2105 | -1.8104 | 1200 | 2100 | 1650 |
| AC | *L. petelotii* | Flora of China | Guangxi, S Guizhou, W Hunan, SE Yunnan [Vietnam] | -14.0583 | -28.1124 | -21.0854 | 1000 | 1800 | 1400 |
| ER | *L. platycarpus* | Flora Malsiana | Malesia: W. and S. Central Java (once collected in SW. Bantam, several times in Nusa Kambanganj | -4.2105 | -4.2105 | -4.2105 | 0 | 500 | 250 |
| AC | *L. pseudokunstleri* | Flora Malsiana | Malesia: Borneo (Sarawak, rather common; Kalimantan, rare; Sabah, rare) | -0.9619 | -5.9788 | -3.4704 | 0 | 1500 | 750 |
| AC | *L. pseudomoluccus* | Flora Malsiana | Malesia: Sumatra (rare), Java (mainly in the western part, eastwards to Mt Jang at 113° 30' E) | 0.5897 | -4.2105 | -1.8104 | 600 | 1700 | 1150 |
| AC | *L. pseudovestitus* | Flora of China | SW Guangdong, SW Guangxi, Hainan, SE Yunnan [N Vietnam] | -14.0583 | -25.0458 | -19.5521 | 200 | 1500 | 850 |
| ER | *L. pseudoxizangensis* | Flora of China | SE Xizang (Mêdog Xian) | -29.6475 | -29.6475 | -29.6475 | 800 | 2000 | 1400 |
| AC | *L. pusillus* | Flora Malsiana | Malesia: Borneo (Sarawak, Kalimantan, Sabah) | -0.9619 | -5.9788 | -3.4704 | 0 | 1800 | 900 |
| AC | *L. rhabdostachyus* | Flora of China | W Guangxi, SE Yunnan [C to N Vietnam] | -14.0583 | -25.0458 | -19.5521 | 900 | 2200 | 1550 |
| AC | *L. rosthornii* | Flora of China | EC to SW Guangdong, S to SW Guangxi, NE Guizhou, Hunan, SE Sichuan | -22.8155 | -30.6512 | -26.7334 | 300 | 900 | 600 |
| AC | *L. silvicolarum* | Flora of China | SW Guangdong, SW Guangxi, Hainan, SE Yunnan [NE Vietnam] | -14.0583 | -25.0458 | -19.5521 | 0 | 1200 | 600 |
| AC | *L. skanianus* | Flora of China | C and S Fujian, Guangdong, Guangxi, Hainan, S Hunan, S Jiangxi, SE Yunnan | -20.0174 | -28.6757 | -24.3466 | 500 | 1000 | 750 |
| AC | *L. sundaicus* | Flora Malsiana | Peninsular Siam (rare), in Malesia: Sumatra (scattered), Malay Peninsula (common; also in Penang and Singapore), Java (common in the western part, scattered in the central and eastern parts, eastwards to Mts Ardjuno, Kawi, Tengger and Idjen), Borneo (scattered in Sarawak, Kalimantan, and Sabah), Philippines (Luzon, Mindoro, rare) | -0.9619 | -12.8797 | -6.9208 | 0 | 2600 | 1300 |
| AC | *L. taitoensis* | Flora of China | Anhui, Fujian, Guangdong, Guangxi, Guizhou, Hubei, Hunan, Jiangsu, Jiangxi, Sichuan, Taiwan, Yunnan, Zhejiang | -22.8155 | -31.8611 | -27.3383 | 1500 | 1500 | 1500 |
| AC | *L. trachycarpus* | Flora of China | S to SW Yunnan [Laos, N Thailand, Vietnam] | -14.0583 | -25.0458 | -19.5521 | 800 | 1300 | 1050 |
| ER | *L. truncatus* | Flora of China | SE Xizang, S Yunnan [NE India, NE Myanmar, N Thailand, N Vietnam]. | -14.0583 | -29.6475 | -21.8529 | 700 | 2200 | 1450 |
| ER | *L. turbinatus* | Flora Malsiana | Malesia: Borneo (N. Sarawak, and se- veral localities in Sabah, especially on Mt Kinabalu) | -0.9619 | -5.9804 | -3.4712 | 1200 | 3000 | 2100 |
| ER | *L. uvariifolius* | Flora of China | Fujian, N to NE Guangdong, Guangxi | -22.8155 | -26.1008 | -24.4582 | 200 | 1000 | 600 |
| ER | *L. variolosus* | Flora of China | SW Sichuan, NW Yunnan [Vietnam] | -25.0458 | -30.6512 | -27.8485 | 2500 | 3000 | 2750 |
| ER | *L. xylocarpus* | Flora of China | SE Xizang, S Yunnan [NE India, N Laos, NE Myanmar, Vietnam] | -14.0583 | -29.6475 | -21.8529 | 1800 | 2300 | 2050 |
