# Supplementary material for: Associated morphometric and geospatial differentiation among 98 species of stone oaks (Lithocarpus)
Source: PLoS One. 2018 Jun 26;13(6):e0199538. doi: 10.1371/journal.pone.0199538 (PMC6019760; doi:10.1371/journal.pone.0199538)
Supplement: S4 Table — (DOCX) [file pone.0199538.s010.docx]

| Fruit  type | Species | 2a | 2b | 3a | 3b | 4a | 4b | 5a | 5b | 6a | 6b | 7a | 7b | 8a | 8b | 9a | 9b | 10a | 10b |
| --- | --- | --- | --- | --- | --- | --- | --- | --- | --- | --- | --- | --- | --- | --- | --- | --- | --- | --- | --- |
| ER | *L. amygdalifolius* (Skan) Hayata | 0.1353 | -0.4362 | 0.0211 | 0.0280 | 0.0383 | -0.1852 | -0.0009 | 0.0397 | 0.0139 | -0.1381 | 0.0034 | 0.0090 | 0.0220 | -0.1119 | 0.0211 | 0.0054 | 0.0201 | -0.0721 |
| AC | *L. bacgiangensis* (Hickel & A.Camus) A.Camus | 0.1041 | -0.4765 | 0.1830 | 0.0274 | 0.0024 | -0.1590 | 0.0962 | -0.0308 | -0.0334 | -0.0823 | 0.0469 | -0.0544 | -0.0143 | -0.0566 | 0.0300 | -0.0533 | -0.0137 | -0.0398 |
| ER | *L. balansae* (Drake) A. Camus | 0.1502 | -0.4236 | 0.0344 | 0.0473 | 0.0854 | -0.1798 | 0.0134 | 0.0387 | 0.0717 | -0.1041 | 0.0011 | 0.0326 | 0.0634 | -0.0641 | -0.0099 | 0.0223 | 0.0564 | -0.0457 |
| AC | *L. bancanus* (Scheff.) Rehder | -0.3099 | -0.4582 | 0.0588 | -0.0447 | -0.1128 | -0.2046 | 0.0518 | -0.0328 | -0.0784 | -0.1251 | 0.0442 | -0.0337 | -0.0375 | -0.0770 | 0.0462 | -0.0163 | -0.0289 | -0.0520 |
| ER | *L. beccarianus* (Benth.) A. Camus | 0.0038 | -0.5158 | 0.0297 | -0.0476 | 0.0012 | -0.2350 | 0.0092 | -0.0127 | 0.0182 | -0.1110 | 0.0226 | 0.0236 | 0.0281 | -0.0502 | 0.0224 | 0.0350 | 0.0286 | -0.0271 |
| AC | *L. bennettii* (Miq.) Rehder | 0.1690 | -0.4886 | -0.0090 | -0.0260 | 0.1265 | -0.2165 | -0.0119 | -0.0125 | 0.0777 | -0.1187 | -0.0203 | -0.0144 | 0.0410 | -0.0955 | -0.0419 | 0.0049 | 0.0238 | -0.0716 |
| AC | *L. blumeanus* (Korth.) Rehder | -0.1920 | -0.5152 | 0.0777 | 0.0173 | -0.0771 | -0.2706 | -0.0120 | 0.0128 | -0.0078 | -0.1686 | 0.0002 | 0.0150 | 0.0483 | -0.1175 | -0.0011 | 0.0038 | 0.0499 | -0.0850 |
| AC | *L. brachystachyus* Chun | 0.1594 | -0.4999 | 0.0138 | 0.0140 | 0.0847 | -0.2446 | 0.0327 | 0.0109 | 0.0528 | -0.1549 | 0.0435 | 0.0077 | 0.0236 | -0.1141 | 0.0376 | 0.0037 | 0.0133 | -0.0863 |
| AC | *L. brevicaudatus* (Skan) Hayata | 0.0527 | -0.5116 | 0.0274 | 0.0136 | 0.0472 | -0.2365 | 0.0542 | 0.0072 | 0.0404 | -0.1470 | 0.0632 | 0.0062 | 0.0275 | -0.0954 | 0.0548 | 0.0086 | 0.0185 | -0.0653 |
| AC | *L. calolepis* Y.C. Hsu & H. Wei Jen | -0.0142 | -0.5112 | 0.0577 | 0.0017 | -0.0248 | -0.2214 | 0.0738 | -0.0114 | -0.0124 | -0.1240 | 0.0589 | -0.0236 | -0.0254 | -0.0730 | 0.0477 | -0.0299 | -0.0237 | -0.0483 |
| AC | *L. calophyllus* Chun ex. C.C. Hunag & Y.T.Chang | 0.0275 | -0.5083 | 0.0711 | 0.0147 | -0.0157 | -0.2126 | 0.0769 | -0.0020 | -0.0251 | -0.1160 | 0.0533 | -0.0220 | -0.0259 | -0.0693 | 0.0427 | -0.0387 | -0.0186 | -0.0483 |
| AC | *L. carolinae* (Skan ex Dunn) Rehder | 0.0678 | -0.5182 | 0.0794 | 0.0361 | -0.0501 | -0.2102 | 0.0860 | -0.0172 | -0.0324 | -0.0980 | 0.0796 | -0.0247 | -0.0218 | -0.0529 | 0.0617 | -0.0307 | -0.0168 | -0.0077 |
| AC | *L. caudatilimbus* (Merr.) A. Camus | 0.0122 | -0.5094 | 0.0608 | 0.0034 | -0.0263 | -0.2274 | 0.0746 | -0.0158 | -0.0224 | -0.1257 | 0.0717 | -0.0267 | -0.0294 | -0.0731 | 0.0582 | -0.0262 | -0.0161 | -0.0517 |
| AC | *L. celebicus* (Miq.) Rehder | 0.2787 | -0.4547 | 0.0176 | -0.0154 | 0.1664 | -0.1701 | -0.0241 | -0.0324 | 0.1160 | -0.0872 | -0.0318 | -0.0686 | 0.0372 | -0.0539 | -0.0164 | -0.0675 | 0.0259 | -0.0306 |
| AC | *L. chrysocomus* Chun & Tsiang | 0.0878 | -0.4957 | 0.0820 | 0.0135 | 0.1310 | -0.2082 | 0.0717 | 0.0272 | 0.0479 | -0.1519 | 0.0184 | 0.0079 | -0.0034 | -0.1170 | -0.0090 | 0.0091 | -0.0136 | -0.0982 |
| ER | *L. cleistocarpus* (Seemen) Rehder & E.H. Wilson | -0.0578 | -0.4877 | 0.0358 | 0.0170 | 0.0035 | -0.2276 | 0.0765 | 0.0041 | -0.0132 | -0.1340 | 0.0608 | -0.0058 | -0.0180 | -0.0887 | 0.0548 | -0.0194 | -0.0172 | -0.0602 |
| AC | *L. confertus* Soepadmo | 0.3057 | -0.4446 | -0.0453 | -0.0497 | 0.1920 | -0.1474 | -0.0448 | -0.0527 | 0.1185 | -0.0590 | -0.0394 | -0.0408 | 0.0742 | -0.0191 | -0.0293 | -0.0366 | 0.0595 | -0.0013 |
| AC | *L. confinis* S.H. Huang ex Y.C. Hsu & H.W. Jen | 0.0029 | -0.5177 | 0.0198 | 0.0104 | -0.0525 | -0.2179 | 0.0664 | -0.0158 | -0.0480 | -0.1104 | 0.0698 | -0.0356 | -0.0321 | -0.0556 | 0.0515 | -0.0468 | -0.0165 | -0.0261 |
| AC | *L. conocarpus* (Oudem.) Rehder | 0.3119 | -0.4413 | -0.0183 | -0.0306 | 0.1509 | -0.1848 | -0.0418 | -0.0320 | 0.0998 | -0.0859 | -0.0383 | -0.0586 | 0.0689 | -0.0626 | -0.0402 | -0.0641 | 0.0308 | -0.0271 |
| ER | *L. corneus* (Lour.) Rehder | 0.0947 | -0.4602 | 0.0462 | -0.0200 | 0.0043 | -0.1452 | 0.0567 | -0.0540 | -0.0045 | -0.0448 | 0.0255 | -0.0665 | 0.0026 | -0.0173 | 0.0003 | -0.0687 | 0.0224 | -0.0197 |
| AC | *L. craibianus* Barnett | -0.0232 | -0.5142 | 0.0633 | 0.0066 | -0.0055 | -0.2232 | 0.0688 | -0.0171 | -0.0412 | -0.1135 | 0.0618 | -0.0399 | -0.0300 | -0.0661 | 0.0411 | -0.0461 | -0.0250 | -0.0358 |
| AC | *L. crassinervius* (Blume) Rehder | -0.0429 | -0.4856 | 0.0502 | -0.0179 | -0.0769 | -0.2320 | 0.0587 | -0.0363 | -0.0619 | -0.1348 | 0.0580 | -0.0106 | -0.0702 | -0.0951 | 0.0544 | -0.0259 | -0.0545 | -0.0651 |
| AC | *L. cryptocarpus* A. Camus * | -0.0002 | -0.4297 | 0.2174 | -0.0164 | -0.0759 | -0.0720 | 0.0985 | -0.1229 | 0.0193 | -0.0147 | 0.0015 | -0.0966 | 0.0417 | -0.0432 | -0.0093 | -0.0485 | 0.0417 | -0.0587 |
| AC | *L. cyclophorus* (Endl.) A. Camus | -0.3423 | -0.3135 | 0.1487 | -0.1281 | -0.1926 | -0.0087 | 0.0619 | -0.0688 | -0.1436 | 0.0005 | 0.0283 | -0.0866 | -0.0836 | -0.0010 | 0.0412 | -0.0852 | -0.0246 | 0.0414 |
| ER | *L. damiaoshanicus* C.C. Huang & Y.T. Chang | 0.0254 | -0.4996 | 0.0706 | 0.0094 | 0.0346 | -0.2218 | 0.0658 | -0.0241 | 0.0108 | -0.1357 | 0.0510 | -0.0176 | 0.0060 | -0.0911 | 0.0405 | -0.0147 | 0.0030 | -0.0693 |
| AC | *L. dasystachyus* (Miq.) Rehder | 0.0390 | -0.5108 | -0.0258 | -0.0197 | 0.0740 | -0.2477 | 0.0619 | 0.0237 | 0.0789 | -0.1432 | 0.0297 | 0.0363 | 0.0665 | -0.0683 | 0.0164 | 0.0397 | 0.0552 | -0.0387 |
| AC | *L. dealbatus* (Hook.f. & Thomson ex Miq.) Rehder | -0.0755 | -0.4947 | 0.0383 | -0.0043 | -0.0271 | -0.1968 | 0.0837 | -0.0139 | -0.0339 | -0.1169 | 0.0901 | -0.0205 | -0.0024 | -0.0598 | 0.0620 | -0.0257 | -0.0045 | -0.0486 |
| AC | *L. echinotholus* (Hu) H.Y. Chun & Huang ex Y.C. Hsu & H.W. Jen | 0.0541 | -0.5144 | 0.0043 | 0.0241 | 0.0818 | -0.2563 | 0.0692 | 0.0283 | 0.0163 | -0.1596 | 0.0737 | -0.0001 | -0.0251 | -0.1052 | 0.0455 | -0.0143 | -0.0324 | -0.0755 |
| AC | *L. edulis* (Makino) Nakai | -0.0004 | -0.5069 | -0.0773 | 0.0116 | 0.0097 | -0.2595 | -0.0124 | 0.0162 | -0.0009 | -0.1669 | 0.0145 | 0.0058 | -0.0073 | -0.1183 | 0.0269 | -0.0043 | -0.0155 | -0.0848 |
| AC | *L. elegans* (Blume) Hatus. ex Soepadmo | -0.1299 | -0.4935 | 0.0723 | -0.0196 | -0.0852 | -0.1931 | 0.0795 | -0.0273 | -0.0613 | -0.0744 | 0.0585 | -0.0492 | -0.0283 | -0.0229 | 0.0357 | -0.0627 | -0.0121 | -0.0100 |
| AC | *L. elmerrillii* Chun | -0.0641 | -0.5033 | 0.0495 | 0.0018 | -0.0338 | -0.2272 | 0.0796 | -0.0140 | -0.0116 | -0.1192 | 0.0701 | -0.0153 | -0.0126 | -0.0684 | 0.0564 | -0.0294 | -0.0107 | -0.0409 |
| AC | *L. encleisocarpus* (Korth.) A. Camus | -0.5234 | 0.0238 | -0.0496 | -0.0488 | 0.0912 | 0.2165 | -0.0056 | 0.0798 | 0.0537 | -0.0965 | 0.0705 | -0.0440 | -0.0628 | -0.0163 | -0.0724 | -0.0391 | -0.0112 | 0.0254 |
| AC | *L. ewyckii* (Korth.) Rehder | 0.1908 | -0.4844 | -0.0221 | -0.0217 | 0.1662 | -0.2000 | -0.0705 | -0.0429 | 0.0894 | -0.1152 | -0.0712 | -0.0374 | 0.0363 | -0.0747 | -0.0615 | 0.0117 | 0.0322 | -0.0453 |
| AC | *L. farinulentus* (Hance) A. Camus | 0.1124 | -0.5220 | 0.0007 | 0.0061 | -0.0115 | -0.2446 | 0.0466 | -0.0057 | -0.0497 | -0.1421 | 0.0440 | -0.0214 | -0.0458 | -0.0997 | 0.0485 | -0.0296 | -0.0398 | -0.0475 |
| AC | *L. fenestratus* (Roxb.) Rehder | -0.0416 | -0.5139 | 0.0373 | 0.0122 | -0.0423 | -0.2254 | 0.0656 | -0.0228 | -0.0492 | -0.1211 | 0.0520 | -0.0412 | -0.0417 | -0.0603 | 0.0328 | -0.0517 | -0.0282 | -0.0382 |
| AC | *L. ferrugineus* Soepadmo | -0.1331 | -0.5129 | 0.0215 | -0.0071 | -0.0409 | -0.2639 | 0.0189 | 0.0051 | -0.0001 | -0.1635 | 0.0259 | -0.0057 | 0.0063 | -0.1211 | 0.0212 | -0.0094 | 0.0180 | -0.0894 |
| AC | *L. fohaiensis* (Hu) A. Camus | -0.0691 | -0.5009 | 0.0558 | -0.0158 | -0.0581 | -0.2013 | 0.0888 | -0.0317 | -0.0325 | -0.1086 | 0.0744 | -0.0493 | -0.0233 | -0.0390 | 0.0587 | -0.0536 | -0.0065 | -0.0228 |
| ER | *L. fordianus* (Hhemsl.) Chun | 0.0421 | -0.4533 | 0.0811 | -0.0132 | -0.0176 | -0.1796 | 0.0690 | -0.0455 | -0.0220 | -0.0852 | 0.0449 | -0.0506 | -0.0281 | -0.0598 | 0.0346 | -0.0506 | -0.0197 | -0.0387 |
| AC | *L. formosanus* (Skan) Hayata | -0.0010 | -0.5122 | 0.0767 | -0.0052 | -0.0036 | -0.1955 | 0.0809 | -0.0338 | -0.0136 | -0.1070 | 0.0799 | -0.0293 | -0.0197 | -0.0584 | 0.0505 | -0.0464 | -0.0101 | -0.0359 |
| AC | *L. glaber* (Thunb.) Nakai | -0.0228 | -0.4979 | 0.0420 | 0.0092 | -0.0080 | -0.2351 | 0.0427 | -0.0024 | -0.0186 | -0.1462 | 0.0491 | 0.0027 | -0.0176 | -0.0969 | 0.0480 | -0.0205 | -0.0206 | -0.0687 |
| AC | *L. glutinosus* (Blume) Soepadmo | 0.4056 | -0.3261 | -0.0186 | -0.0952 | 0.1821 | 0.0013 | 0.0561 | -0.0892 | 0.0711 | 0.0603 | 0.0562 | -0.0244 | 0.0205 | 0.0358 | 0.0750 | 0.0256 | -0.0120 | 0.0214 |
| AC | *L. gracilis* (Korth.) Soepadmo | 0.4553 | -0.2866 | 0.0040 | -0.0345 | 0.2598 | 0.0585 | 0.0153 | -0.0270 | 0.1351 | 0.0739 | 0.0327 | -0.0556 | 0.0708 | 0.0842 | 0.0353 | -0.0351 | -0.0240 | 0.0453 |
| AC | *L. hancei* (Benth.) Rehder | -0.0341 | -0.5125 | 0.0227 | -0.0066 | -0.0087 | -0.2365 | 0.0728 | -0.0036 | -0.0196 | -0.1308 | 0.0721 | -0.0275 | -0.0260 | -0.0793 | 0.0603 | -0.0340 | -0.0227 | -0.0431 |
| AC | *L. handelianus* A. Camus | 0.0611 | -0.5075 | 0.0598 | 0.0188 | -0.0334 | -0.2273 | 0.0707 | -0.0127 | -0.0514 | -0.1125 | 0.0571 | -0.0402 | -0.0387 | -0.0644 | 0.0338 | -0.0455 | -0.0309 | -0.0335 |
| AC | *L. harlandii* (Hance ex Walp.) Rehder | -0.0675 | -0.4997 | 0.0482 | 0.0070 | -0.0076 | -0.2124 | 0.0778 | -0.0219 | -0.0223 | -0.1049 | 0.0555 | -0.0317 | -0.0240 | -0.0589 | 0.0414 | -0.0435 | -0.0206 | -0.0367 |
| AC | *L. henryi* (Seemen) Rehder & E.H. Wilson | -0.0480 | -0.5165 | 0.0338 | 0.0004 | -0.0293 | -0.2345 | 0.0637 | -0.0097 | -0.0393 | -0.1262 | 0.0668 | -0.0261 | -0.0338 | -0.0738 | 0.0497 | -0.0410 | -0.0290 | -0.0467 |
| AC | *L. himalaicus* C. C.Huang & Y.T. Chang | 0.0770 | -0.5095 | 0.0274 | 0.0048 | 0.0140 | -0.2392 | 0.0507 | -0.0034 | 0.0102 | -0.1501 | 0.0457 | 0.0048 | -0.0105 | -0.1066 | 0.0553 | -0.0105 | -0.0182 | -0.0693 |
| AC | *L. howii* Chun | 0.1766 | -0.5003 | 0.0697 | 0.0101 | 0.0142 | -0.2318 | 0.0628 | 0.0054 | -0.0253 | -0.1577 | 0.0500 | -0.0045 | -0.0053 | -0.1122 | 0.0272 | 0.0106 | 0.0060 | -0.0908 |
| AC | *L. hypoglaucus* (Hu) C.C. Huang ex Y.C. Hsu & H.W. Jen | -0.1196 | -0.5276 | 0.0416 | -0.0023 | -0.0508 | -0.2096 | 0.0824 | -0.0280 | -0.0492 | -0.1024 | 0.0887 | -0.0428 | -0.0215 | -0.0373 | 0.0543 | -0.0482 | -0.0095 | -0.0164 |
| AC | *L. indutus* (Blume) Rehder * | -0.1552 | -0.4030 | 0.1063 | -0.0530 | -0.1348 | -0.0760 | 0.0510 | -0.1135 | -0.0351 | -0.0602 | 0.0460 | -0.1099 | 0.0280 | -0.0043 | 0.0320 | -0.0508 | 0.0395 | 0.0130 |
| AC | *L. jacobsii* Soepadmo | 0.2630 | -0.4746 | 0.0012 | -0.0242 | 0.1665 | -0.1945 | 0.0067 | -0.0090 | 0.1237 | -0.1397 | -0.0162 | -0.0112 | 0.0929 | -0.0956 | -0.0034 | -0.0129 | 0.0636 | -0.0755 |
| ER | *L. javensis* Blume | 0.3339 | -0.2746 | 0.0492 | -0.0229 | 0.1324 | -0.1341 | -0.0008 | 0.0592 | 0.1125 | -0.0449 | -0.0062 | 0.0477 | 0.0711 | -0.0248 | -0.0225 | 0.0394 | 0.0528 | -0.0108 |
| AC | *L. kawakamii* (Hayata) Hayata | -0.0806 | -0.5125 | 0.1135 | -0.0215 | -0.0666 | -0.1813 | 0.0973 | -0.0413 | -0.0546 | -0.0645 | 0.0528 | -0.0667 | -0.0220 | -0.0173 | 0.0171 | -0.0629 | 0.0045 | -0.0096 |
| AC | *L. konishii* (Hayata) Hayata * | -0.0255 | -0.4706 | 0.0659 | -0.0127 | -0.0718 | -0.1711 | 0.0728 | -0.0609 | -0.0541 | -0.0757 | 0.0542 | -0.0807 | -0.0292 | -0.0274 | 0.0220 | -0.0805 | -0.0034 | -0.0060 |
| ER | *L. lampadarius* (Gamble) A. Camus * | -0.1681 | -0.4677 | 0.0446 | -0.0240 | -0.0423 | -0.2047 | 0.0686 | -0.0109 | -0.0155 | -0.1135 | 0.0570 | -0.0177 | 0.0055 | -0.0812 | 0.0600 | -0.0098 | 0.0187 | -0.0487 |
| ER | *L. laoticus* (Hhickel & A. Camus) A. Camus | 0.0804 | -0.4546 | 0.0668 | 0.0045 | 0.0152 | -0.2189 | 0.0075 | 0.0352 | 0.0002 | -0.1402 | 0.0424 | 0.0236 | -0.0121 | -0.1064 | 0.0187 | -0.0105 | -0.0200 | -0.0770 |
| AC | *L. lappaceus* (Roxb.) Rehder | 0.3160 | -0.4215 | -0.0116 | -0.0188 | 0.1928 | -0.1580 | -0.0195 | -0.0743 | 0.1309 | -0.0642 | 0.0054 | -0.0560 | 0.1035 | 0.0192 | 0.0099 | -0.0430 | 0.0617 | 0.0233 |
| ER | *L. lepidocarpus* (Hayata) Hayata | -0.1317 | -0.3432 | 0.1494 | 0.0028 | -0.0273 | -0.1487 | 0.1346 | -0.0809 | -0.0342 | -0.0740 | 0.0771 | -0.0665 | -0.0103 | -0.0212 | 0.0502 | -0.0459 | -0.0046 | -0.0193 |
| AC | *L. leptogyne* (Korth.) Soepadmo | 0.2350 | -0.4630 | -0.0527 | -0.0399 | 0.1214 | -0.1942 | -0.0451 | -0.0253 | 0.0929 | -0.1121 | -0.0337 | -0.0192 | 0.0754 | -0.0612 | -0.0331 | -0.0350 | 0.0687 | -0.0291 |
| AC | *L. lindleyanus* (Wall. ex A. DC.) A. Camus | 0.0251 | -0.5080 | 0.0187 | 0.0062 | -0.0259 | -0.2311 | 0.0205 | -0.0062 | -0.0258 | -0.1529 | 0.0297 | -0.0096 | -0.0333 | -0.0954 | 0.0319 | -0.0249 | -0.0294 | -0.0632 |
| AC | *L. litseifolius* (Hance) Chun | 0.0254 | -0.5149 | 0.1006 | 0.0110 | 0.0531 | -0.2268 | 0.0902 | 0.0070 | -0.0165 | -0.1235 | 0.0628 | -0.0106 | -0.0392 | -0.0785 | 0.0451 | -0.0268 | -0.0313 | -0.0516 |
| AC | *L. longanoides* C.C. Huang & Y.T. Chang | 0.0248 | -0.5164 | 0.0444 | 0.0211 | -0.0128 | -0.2268 | 0.0775 | -0.0131 | -0.0614 | -0.1097 | 0.0502 | -0.0501 | -0.0481 | -0.0615 | 0.0401 | -0.0563 | -0.0365 | -0.0328 |
| AC | *L. longipedicellatus* (Hickel & A. Camus) A. Camus | 0.0260 | -0.5065 | 0.0979 | 0.0045 | 0.0157 | -0.2161 | 0.0631 | -0.0122 | -0.0308 | -0.1282 | 0.0581 | -0.0328 | -0.0291 | -0.0705 | 0.0455 | -0.0380 | -0.0275 | -0.0420 |
| AC | *L. lucidus* (Roxb.) Rehder | -0.1443 | -0.4822 | 0.0579 | -0.0229 | -0.1156 | -0.1823 | 0.0646 | -0.0476 | -0.0353 | -0.1097 | 0.0794 | -0.0177 | -0.0043 | -0.0523 | 0.0438 | 0.0064 | -0.0225 | -0.0547 |
| AC | *L. luteus* Soepadmo | 0.3039 | -0.4777 | -0.0109 | -0.0597 | 0.0275 | -0.1930 | 0.0531 | -0.0039 | 0.0207 | -0.1986 | -0.0245 | 0.0048 | 0.0373 | -0.1153 | -0.0083 | 0.0003 | 0.0211 | -0.0984 |
| AC | *L. mairei* (Schottky) Rehder | -0.0348 | -0.5078 | 0.0187 | 0.0051 | 0.0056 | -0.2397 | 0.0586 | 0.0036 | 0.0032 | -0.1247 | 0.0553 | -0.0053 | -0.0033 | -0.0889 | 0.0525 | -0.0088 | -0.0057 | -0.0603 |
| ER | *L. megacarpus* Soepodmo | 0.3246 | -0.3464 | -0.0516 | -0.1133 | 0.1114 | -0.0102 | -0.0077 | -0.0807 | 0.0164 | 0.0344 | 0.0105 | -0.0686 | -0.0315 | -0.0177 | 0.0250 | -0.0521 | -0.0297 | -0.0244 |
| AC | *L. meijeri* Soepadmo | -0.3563 | -0.4717 | -0.0347 | -0.0137 | -0.1011 | -0.2362 | -0.0180 | -0.0170 | -0.0549 | -0.1622 | -0.0160 | 0.0006 | -0.0305 | -0.1191 | 0.0031 | -0.0037 | -0.0282 | -0.0960 |
| AC | *L. naiadarum* (Hance) Chun | 0.0175 | -0.5240 | 0.0375 | 0.0155 | -0.0233 | -0.2206 | 0.0791 | -0.0017 | -0.0239 | -0.1097 | 0.0779 | -0.0340 | -0.0208 | -0.0515 | 0.0658 | -0.0371 | -0.0109 | -0.0221 |
| AC | *L. nieuwenhuisii* (Seemen) A. Camus | 0.0396 | -0.4927 | 0.0406 | 0.0251 | 0.1261 | -0.2075 | -0.0298 | -0.0202 | 0.1164 | -0.0984 | -0.0297 | -0.0340 | 0.0761 | -0.0633 | -0.0162 | -0.0219 | 0.0721 | -0.0422 |
| AC | *L. nodosus* Soepadmo | -0.1253 | -0.5220 | -0.0730 | 0.0312 | -0.0233 | -0.2144 | -0.0783 | -0.0012 | 0.0374 | -0.1121 | -0.0576 | -0.0276 | 0.0509 | -0.0704 | -0.0318 | -0.0528 | 0.0413 | -0.0525 |
| AC | *L. oblanceolatus* C.C. Huang & Y.T. Chang | 0.0252 | -0.5205 | -0.0389 | 0.0129 | -0.0351 | -0.2512 | 0.0204 | 0.0088 | -0.0468 | -0.1364 | 0.0483 | -0.0114 | -0.0370 | -0.0810 | 0.0543 | -0.0097 | -0.0283 | -0.0498 |
| AC | *L. obscurus* C.C. Huang & Y.T. Chang | 0.0595 | -0.5142 | -0.0187 | 0.0087 | 0.0073 | -0.2538 | 0.0364 | 0.0017 | -0.0155 | -0.1556 | 0.0457 | -0.0084 | -0.0213 | -0.1042 | 0.0517 | -0.0135 | -0.0227 | -0.0705 |
| ER | *L. pachylepis* A. Camus | -0.0290 | -0.4735 | 0.0941 | -0.0259 | 0.0001 | -0.1847 | 0.0868 | -0.0427 | 0.0162 | -0.0875 | 0.0685 | -0.0417 | 0.0274 | -0.0493 | 0.0509 | -0.0402 | 0.0332 | -0.0339 |
| ER | *L. pachyphyllus* (Kruz) Rehder | -0.0137 | -0.3069 | 0.0563 | 0.0403 | 0.0411 | -0.1012 | 0.0517 | 0.0027 | 0.0408 | -0.0430 | 0.0368 | -0.0197 | 0.0145 | 0.0068 | 0.0271 | -0.0129 | 0.0165 | 0.0228 |
| AC | *L. pallidus* (Blume) Rehder | 0.3132 | -0.4302 | -0.0071 | -0.0407 | 0.1863 | -0.1210 | -0.0268 | -0.0548 | 0.0988 | -0.0483 | 0.0015 | -0.1017 | 0.0387 | -0.0277 | 0.0191 | -0.0780 | 0.0289 | -0.0086 |
| AC | *L. petelotii* A. Camus | -0.0040 | -0.5164 | 0.0542 | 0.0103 | -0.0714 | -0.2250 | 0.0528 | -0.0061 | -0.0599 | -0.1076 | 0.0436 | -0.0239 | -0.0445 | -0.0569 | 0.0331 | -0.0371 | -0.0264 | -0.0304 |
| ER | *L. platycarpus* (Blume) Rehder | 0.4037 | -0.3116 | -0.0380 | -0.0934 | 0.1501 | 0.0292 | 0.0111 | -0.0983 | 0.0117 | 0.0722 | 0.0469 | -0.0637 | -0.0342 | 0.0348 | 0.0486 | -0.0089 | -0.0248 | -0.0421 |
| AC | *L. polystachyus* (Wall. ex A. DC.) Rehder | 0.0572 | -0.5177 | 0.0813 | 0.0032 | -0.0320 | -0.2116 | 0.0790 | -0.0275 | -0.0143 | -0.1073 | 0.0405 | -0.0307 | -0.0087 | -0.0634 | 0.0273 | -0.0293 | -0.0102 | -0.0522 |
| AC | *L. pseudokunstleri* A. Camus | 0.0698 | -0.5013 | 0.0512 | -0.0001 | 0.0072 | -0.2503 | 0.0042 | -0.0031 | -0.0085 | -0.1534 | -0.0126 | -0.0042 | 0.0063 | -0.1051 | -0.0125 | -0.0090 | 0.0168 | -0.0852 |
| AC | *L. pseudomoluccus* (Blume) Rehder | 0.4003 | -0.3701 | -0.0589 | -0.1115 | 0.2030 | -0.0570 | -0.0110 | -0.0872 | 0.1067 | -0.0324 | -0.0338 | -0.0678 | 0.0786 | -0.0191 | -0.0240 | -0.0833 | 0.0402 | 0.0254 |
| AC | *L. pseudovestitus* A. Camus | 0.0411 | -0.4979 | 0.1519 | -0.0034 | -0.0512 | -0.1715 | 0.0683 | -0.0454 | -0.0460 | -0.1026 | 0.0477 | -0.0563 | -0.0310 | -0.0623 | 0.0320 | -0.0523 | -0.0289 | -0.0412 |
| ER | *L. pseudoxizangensis* Z.K. Zhou & H. Sun | -0.0714 | -0.4809 | 0.0571 | 0.0154 | -0.0036 | -0.1910 | 0.0759 | 0.0040 | -0.0077 | -0.0973 | 0.0704 | -0.0167 | -0.0106 | -0.0623 | 0.0557 | -0.0246 | -0.0125 | -0.0390 |
| AC | *L. pusillus* Soepadmo | -0.1872 | -0.4832 | -0.0347 | -0.0139 | -0.1213 | -0.2343 | 0.0348 | -0.0150 | -0.0768 | -0.1225 | 0.0269 | -0.0088 | -0.0481 | -0.0850 | 0.0160 | -0.0044 | -0.0356 | -0.0716 |
| AC | *L. rhabdostachyus* (Hickel & A. Camus) A. Camus | 0.0933 | -0.5004 | 0.0834 | 0.0316 | 0.0153 | -0.2175 | 0.0538 | 0.0162 | -0.0298 | -0.1304 | 0.0417 | -0.0107 | -0.0319 | -0.0928 | 0.0371 | -0.0235 | -0.0308 | -0.0659 |
| AC | *L. rosthornii* (Schottky) Barnett | 0.1359 | -0.4997 | 0.0363 | 0.0089 | 0.0476 | -0.2390 | 0.0427 | 0.0062 | 0.0216 | -0.1526 | 0.0388 | 0.0045 | 0.0124 | -0.1110 | 0.0329 | 0.0010 | 0.0104 | -0.0847 |
| AC | *L. silvicolarum* (Hance) Chun | 0.0689 | -0.5129 | 0.0795 | 0.0325 | -0.0138 | -0.1988 | 0.0780 | 0.0239 | -0.0317 | -0.1104 | 0.0738 | -0.0094 | -0.0280 | -0.0628 | 0.0593 | -0.0336 | -0.0236 | -0.0387 |
| AC | *L. skanianus* (Dunn) Rehder | 0.2440 | -0.5073 | -0.0668 | 0.0721 | 0.0834 | -0.2390 | 0.0162 | 0.0464 | -0.0184 | -0.1557 | 0.0377 | 0.0084 | -0.0277 | -0.1087 | 0.0380 | -0.0034 | -0.0292 | -0.0773 |
| AC | *L. sundaicus* (Blume) Rehder | -0.3844 | -0.3400 | 0.0090 | 0.0021 | -0.2414 | 0.0377 | -0.0084 | -0.0185 | -0.0060 | 0.1507 | -0.0122 | 0.0037 | 0.0836 | 0.0563 | -0.0050 | 0.0288 | 0.0781 | -0.0332 |
| AC | *L. taitoensis* (Hayata) Hayata | 0.0394 | -0.5088 | 0.0872 | 0.0120 | 0.0470 | -0.2326 | 0.0880 | 0.0072 | 0.0070 | -0.1312 | 0.0546 | -0.0079 | -0.0210 | -0.0896 | 0.0336 | -0.0231 | -0.0221 | -0.0634 |
| AC | *L. touranensis* (Hickel & A. Camus) A. Camus | 0.0860 | -0.5132 | 0.0978 | 0.0273 | 0.0592 | -0.2136 | 0.1032 | 0.0385 | 0.0194 | -0.1075 | 0.0926 | 0.0257 | 0.0050 | -0.0624 | 0.0694 | -0.0058 | 0.0068 | -0.0434 |
| AC | *L. trachycarpus* (Hickel & A.Camus) A. Camus | -0.0472 | -0.5023 | 0.0084 | 0.0010 | 0.0352 | -0.2367 | 0.0633 | 0.0150 | -0.0034 | -0.1344 | 0.0700 | -0.0090 | 0.0026 | -0.0785 | 0.0631 | -0.0092 | -0.0140 | -0.0565 |
| ER | *L. truncatus* (King ex Hook. f.) Rheder | 0.1113 | -0.4544 | 0.0740 | 0.0119 | 0.0631 | -0.1752 | 0.0700 | 0.0027 | 0.0202 | -0.1028 | 0.0455 | 0.0056 | -0.0075 | -0.0611 | 0.0264 | -0.0100 | -0.0152 | -0.0419 |
| ER | *L. turbinatus* (Stapf) Forman | -0.0756 | -0.2237 | 0.2221 | -0.3165 | -0.1786 | -0.3307 | -0.2543 | -0.1711 | 0.0123 | -0.1161 | 0.0573 | -0.1734 | -0.1217 | -0.1459 | -0.1334 | -0.0949 | 0.0233 | -0.0560 |
| ER | *L. uvariifolius* (Hance) Rehder | -0.0069 | -0.4198 | 0.1082 | -0.0516 | 0.0104 | -0.1283 | 0.0498 | -0.0725 | 0.0284 | -0.0739 | 0.0338 | -0.0445 | 0.0209 | -0.0542 | 0.0289 | -0.0335 | 0.0157 | -0.0393 |
| ER | *L. variolosus* (Franch.) Chun | -0.1299 | -0.4422 | 0.0532 | -0.0219 | -0.0593 | -0.1757 | 0.0882 | -0.0514 | -0.0601 | -0.0582 | 0.0704 | -0.0519 | -0.0387 | -0.0150 | 0.0506 | -0.0712 | -0.0173 | -0.0013 |
| AC | *L. vestitus* (Hickel & A. Camus) A. Camus | 0.1115 | -0.5028 | 0.1272 | 0.0043 | -0.0102 | -0.2282 | 0.0546 | -0.0145 | -0.0346 | -0.1405 | 0.0341 | -0.0177 | -0.0290 | -0.0900 | 0.0393 | -0.0180 | -0.0333 | -0.0672 |
| ER | *L. xylocarpus* (Kurz) Markgr. | 0.0485 | -0.4635 | 0.0909 | -0.0001 | 0.0040 | -0.2373 | 0.0465 | 0.0260 | -0.0112 | -0.1337 | 0.0511 | -0.0016 | -0.0153 | -0.0952 | 0.0457 | 0.0020 | -0.0197 | -0.0681 |
