# Supplementary material for: Associated morphometric and geospatial differentiation among 98 species of stone oaks (Lithocarpus)
Source: PLoS One. 2018 Jun 26;13(6):e0199538. doi: 10.1371/journal.pone.0199538 (PMC6019760; doi:10.1371/journal.pone.0199538)
Supplement: S5 Table — (DOCX) [file pone.0199538.s011.docx]

| Fruit type | Species | 2a | 2b | 3a | 3b | 4a | 4b | 5a | 5b | 6a | 6b | 7a | 7b | 8a | 8b | 9a | 9b | 10a | 10b |
| --- | --- | --- | --- | --- | --- | --- | --- | --- | --- | --- | --- | --- | --- | --- | --- | --- | --- | --- | --- |
| ER | *L. amygdalifolius* (Skan) Hayata | -0.2041 | -0.5278 | 0.0100 | 0.0106 | -0.1149 | -0.2178 | 0.1090 | -0.0733 | -0.0609 | -0.0584 | 0.0630 | -0.1011 | -0.0541 | -0.0077 | -0.0020 | -0.1302 | 0.0296 | 0.0085 |
| AC | *L. bacgiangensis* (Hickel & A.Camus) A.Camus | -0.1038 | -0.4785 | -0.1283 | 0.0357 | -0.0501 | -0.2207 | 0.0359 | -0.0016 | -0.0848 | -0.1151 | 0.0539 | -0.0426 | -0.0327 | -0.0989 | 0.0598 | -0.0412 | -0.0572 | -0.0297 |
| ER | *L. balansae* (Drake) A. Camus | -0.0732 | -0.5435 | 0.0619 | -0.0110 | -0.0674 | -0.2231 | 0.0562 | -0.0229 | -0.0770 | -0.0961 | 0.0730 | -0.0706 | -0.0484 | -0.0496 | 0.0687 | -0.0765 | -0.0251 | -0.0333 |
| AC | *L. bancanus* (Scheff.) Rehder | 0.0038 | -0.4710 | 0.1155 | 0.0433 | 0.1135 | -0.2812 | 0.0529 | -0.0387 | 0.0327 | -0.0907 | 0.0541 | 0.0337 | 0.0325 | -0.0400 | 0.0424 | 0.0163 | 0.0499 | -0.0138 |
| ER | *L. beccarianus* (Benth.) A. Camus | -0.1842 | -0.5249 | 0.1941 | -0.1463 | -0.1725 | -0.0540 | 0.0219 | -0.0447 | -0.0884 | -0.0428 | 0.0316 | -0.0593 | -0.0360 | 0.0122 | -0.0149 | -0.0653 | 0.0082 | -0.0022 |
| AC | *L. bennettii* (Miq.) Rehder | -0.1462 | -0.5018 | -0.1608 | 0.0671 | -0.0973 | -0.2578 | -0.0392 | 0.0510 | -0.0899 | -0.0957 | 0.0638 | -0.0472 | -0.0570 | -0.0450 | 0.0508 | -0.0879 | -0.0099 | 0.0049 |
| AC | *L. blumeanus* (Korth.) Rehder | 0.1522 | -0.4072 | -0.1710 | -0.0826 | 0.1457 | -0.2393 | -0.0277 | 0.0084 | 0.0560 | -0.0841 | -0.0117 | -0.1443 | 0.1039 | -0.0038 | -0.0572 | -0.0396 | -0.0047 | -0.0406 |
| AC | *L. brachystachyus* Chun | -0.1501 | -0.5141 | -0.0909 | 0.0516 | -0.1097 | -0.1644 | 0.0646 | -0.0340 | -0.0369 | -0.0814 | 0.0633 | -0.1244 | -0.0607 | -0.0362 | 0.0294 | -0.0900 | -0.0324 | 0.0035 |
| AC | *L. brevicaudatus* (Skan) Hayata | -0.1582 | -0.4831 | -0.0249 | 0.0147 | -0.0703 | -0.2139 | 0.0331 | 0.0039 | -0.0001 | -0.1631 | 0.0255 | -0.0433 | -0.0280 | -0.1059 | 0.0023 | -0.0417 | -0.0339 | -0.0453 |
| AC | *L. calolepis* Y.C. Hsu & H. Wei Jen | -0.0918 | -0.4875 | -0.0844 | 0.0127 | -0.0806 | -0.2399 | 0.0268 | -0.0128 | -0.0619 | -0.1263 | 0.0553 | -0.0314 | -0.0488 | -0.0763 | 0.0584 | -0.0445 | -0.0390 | -0.0469 |
| AC | *L. calophyllus* Chun ex. C.C. Hunag & Y.T.Chang | -0.1225 | -0.4928 | 0.0001 | 0.0117 | -0.0625 | -0.1860 | 0.0162 | -0.0032 | -0.0230 | -0.0850 | 0.0781 | -0.0764 | -0.0481 | -0.0523 | 0.0502 | -0.0794 | -0.0674 | -0.0181 |
| AC | *L. carolinae* (Skan ex Dunn) Rehder | -0.0181 | -0.5041 | -0.0231 | 0.0138 | -0.0084 | -0.2417 | -0.0024 | 0.0040 | -0.0106 | -0.1304 | 0.0117 | 0.0015 | -0.0130 | -0.0825 | 0.0206 | -0.0101 | -0.0067 | -0.0486 |
| AC | *L. caudatilimbus* (Merr.) A. Camus | -0.0954 | -0.5107 | -0.1136 | 0.0157 | -0.0792 | -0.1980 | -0.1428 | 0.0344 | -0.0042 | -0.1248 | -0.0805 | 0.0041 | -0.0132 | -0.1175 | -0.0610 | 0.0009 | -0.0238 | -0.0959 |
| AC | *L. celebicus* (Miq.) Rehder | 0.0548 | -0.4263 | 0.1950 | 0.0379 | 0.0838 | -0.1942 | 0.0319 | -0.0236 | -0.0201 | -0.0936 | 0.0325 | 0.0395 | 0.0338 | -0.0145 | 0.0330 | -0.0042 | 0.0141 | -0.0247 |
| AC | *L. chrysocomus* Chun & Tsiang | -0.1069 | -0.5429 | -0.1583 | 0.0364 | -0.0730 | -0.2712 | 0.0364 | -0.0148 | -0.0798 | -0.1214 | 0.0954 | -0.0665 | -0.0950 | -0.0784 | -0.0082 | -0.0142 | -0.0439 | -0.0636 |
| ER | *L. cleistocarpus* (Seemen) Rehder & E.H. Wilson | -0.0878 | -0.5297 | -0.0503 | 0.0276 | -0.0334 | -0.2644 | 0.0397 | 0.0050 | -0.0722 | -0.1416 | 0.0609 | -0.0188 | -0.0603 | -0.0715 | 0.0493 | -0.0585 | -0.0515 | -0.0557 |
| AC | *L. confertus* Soepadmo | -0.1287 | -0.4777 | 0.0153 | -0.0078 | -0.0505 | -0.2450 | -0.0290 | 0.0273 | -0.0933 | -0.1368 | -0.0127 | -0.0183 | -0.0296 | -0.0677 | -0.0190 | 0.0138 | -0.0561 | -0.0530 |
| AC | *L. confinis* S.H. Huang ex Y.C. Hsu & H.W. Jen | -0.0809 | -0.4909 | -0.1160 | 0.0391 | -0.0210 | -0.1887 | 0.0430 | -0.0304 | 0.0055 | -0.1460 | 0.1031 | -0.0598 | -0.0442 | -0.1058 | 0.0811 | -0.0261 | -0.0178 | -0.0443 |
| AC | *L. conocarpus* (Oudem.) Rehder | 0.4706 | -0.2701 | 0.0338 | -0.0519 | 0.1397 | 0.2526 | 0.0190 | -0.0510 | -0.0668 | 0.0328 | -0.0298 | 0.0006 | 0.0242 | -0.0413 | 0.0482 | -0.0263 | -0.0035 | 0.0273 |
| ER | *L. corneus* (Lour.) Rehder | -0.3019 | -0.4913 | -0.0034 | 0.0268 | -0.2134 | -0.1286 | 0.0852 | -0.2571 | 0.1254 | 0.0093 | -0.0196 | -0.0184 | 0.0580 | -0.0604 | -0.0629 | -0.0649 | 0.0386 | -0.0962 |
| AC | *L. craibianus* Barnett | 0.0312 | -0.4695 | 0.0163 | -0.0038 | 0.0141 | -0.2148 | 0.0141 | -0.0008 | 0.0091 | -0.1184 | 0.0301 | -0.0044 | 0.0152 | -0.0694 | 0.0225 | -0.0007 | 0.0288 | -0.0511 |
| AC | *L. crassinervius* (Blume) Rehder | 0.0128 | -0.5276 | -0.0800 | 0.0155 | -0.0031 | -0.2614 | 0.0333 | 0.0224 | -0.0082 | -0.1217 | 0.0538 | -0.0092 | -0.0075 | -0.0642 | 0.0453 | -0.0240 | -0.0108 | -0.0348 |
| AC | *L. cryptocarpus* A. Camus * | -0.1245 | -0.4708 | -0.2502 | 0.0690 | -0.0860 | -0.2960 | 0.3316 | -0.1775 | 0.0227 | 0.0804 | 0.0790 | -0.1104 | -0.0160 | -0.0062 | 0.0520 | -0.0781 | 0.0119 | 0.0081 |
| AC | *L. cyclophorus* (Endl.) A. Camus | -0.1666 | -0.2474 | 0.2903 | -0.2918 | -0.3340 | -0.1742 | -0.0381 | 0.0973 | -0.0547 | 0.0292 | -0.0259 | -0.1701 | -0.1030 | 0.0394 | 0.0369 | 0.0349 | 0.0054 | -0.0414 |
| ER | *L. damiaoshanicus* C.C. Huang & Y.T. Chang | -0.0767 | -0.5410 | -0.0434 | 0.0081 | -0.0529 | -0.2586 | 0.0035 | 0.0068 | -0.0582 | -0.1454 | 0.0943 | -0.0482 | -0.0585 | -0.1019 | 0.0393 | -0.0089 | -0.0243 | -0.0813 |
| AC | *L. dasystachyus* (Miq.) Rehder | -0.0803 | -0.4133 | 0.0754 | 0.0390 | 0.0111 | -0.1435 | 0.0603 | 0.0375 | 0.0443 | -0.0216 | 0.0134 | 0.0123 | 0.0178 | 0.0167 | -0.0055 | -0.0247 | 0.0101 | 0.0213 |
| AC | *L. dealbatus* (Hook.f. & Thomson ex Miq.) Rehder | -0.1026 | -0.4984 | -0.0136 | -0.0025 | -0.0382 | -0.2264 | 0.0337 | -0.0012 | -0.0605 | -0.1357 | 0.0509 | -0.0339 | -0.0593 | -0.1032 | 0.0669 | -0.0563 | -0.0468 | -0.0502 |
| AC | *L. echinotholus* (Hu) H.Y. Chun & Huang ex Y.C. Hsu & H.W. Jen | -0.0516 | -0.4749 | -0.0775 | -0.0021 | -0.1564 | -0.2312 | -0.0889 | 0.0294 | -0.0257 | -0.1675 | 0.0159 | -0.0210 | 0.0393 | -0.1410 | 0.0320 | -0.0007 | -0.0338 | -0.0913 |
| AC | *L. edulis* (Makino) Nakai | -0.0340 | -0.4760 | 0.0667 | -0.0340 | 0.0096 | -0.1723 | 0.1418 | -0.0453 | -0.0359 | -0.0772 | 0.1394 | -0.0759 | -0.0257 | -0.0359 | 0.0899 | -0.0556 | 0.0057 | 0.0142 |
| AC | *L. elegans* (Blume) Hatus. ex Soepadmo | -0.0124 | -0.5031 | -0.0090 | 0.0112 | -0.0954 | -0.1905 | 0.0179 | 0.0046 | -0.0036 | -0.1053 | 0.0432 | -0.0260 | 0.0148 | -0.0810 | 0.0387 | -0.0557 | -0.0265 | -0.0705 |
| AC | *L. elmerrillii* Chun | -0.0437 | -0.4950 | 0.0446 | 0.0192 | -0.0788 | -0.2344 | 0.0060 | 0.0127 | -0.0386 | -0.1368 | 0.0663 | -0.0115 | -0.0388 | -0.1102 | 0.0557 | -0.0198 | -0.0184 | -0.0552 |
| AC | *L. encleisocarpus* (Korth.) A. Camus | 0.4461 | -0.1523 | 0.0832 | -0.1864 | -0.1121 | 0.1529 | -0.0334 | -0.1808 | 0.1851 | -0.2043 | 0.0691 | -0.0329 | 0.1832 | -0.0283 | 0.0884 | 0.1817 | -0.1227 | -0.0166 |
| AC | *L. ewyckii* (Korth.) Rehder | 0.1311 | -0.4282 | -0.1993 | -0.0171 | 0.0382 | -0.2029 | -0.0840 | -0.0075 | 0.0313 | -0.1437 | -0.0628 | -0.1741 | 0.0756 | 0.0235 | -0.0838 | 0.0061 | -0.0594 | -0.0261 |
| AC | *L. farinulentus* (Hance) A. Camus | 0.1222 | -0.4690 | -0.1194 | -0.0174 | 0.0415 | -0.1458 | -0.0586 | -0.0513 | 0.0446 | -0.0460 | -0.0019 | -0.0232 | 0.0374 | -0.0250 | 0.0050 | -0.0451 | 0.0280 | -0.0193 |
| AC | *L. fenestratus* (Roxb.) Rehder | 0.0072 | -0.4845 | 0.0235 | -0.0276 | 0.0231 | -0.2185 | 0.0017 | 0.0370 | -0.0396 | -0.1408 | 0.0289 | 0.0123 | -0.0304 | -0.0955 | 0.0338 | 0.0058 | -0.0163 | -0.0646 |
| AC | *L. ferrugineus* Soepadmo | 0.0332 | -0.5192 | 0.0783 | 0.0014 | 0.0943 | -0.2154 | 0.1254 | 0.0225 | 0.0312 | -0.1250 | 0.0362 | 0.0286 | 0.0068 | -0.1016 | -0.0056 | 0.0242 | 0.0056 | -0.0726 |
| AC | *L. fohaiensis* (Hu) A. Camus | -0.0676 | -0.5161 | -0.1279 | 0.0392 | -0.0835 | -0.2226 | 0.0148 | -0.0020 | -0.0539 | -0.1040 | 0.1093 | -0.0683 | -0.0580 | -0.0918 | 0.0599 | -0.0870 | -0.0547 | -0.0601 |
| ER | *L. fordianus* (Hhemsl.) Chun | -0.4025 | -0.3939 | 0.0431 | -0.0989 | -0.1433 | -0.0129 | -0.0811 | -0.2325 | 0.1558 | -0.1270 | 0.0591 | 0.0507 | -0.0168 | -0.0437 | 0.0063 | -0.0203 | -0.0595 | -0.0735 |
| AC | *L. formosanus* (Skan) Hayata | -0.0786 | -0.5111 | -0.0264 | 0.0242 | -0.0286 | -0.2077 | 0.1141 | -0.0412 | -0.0327 | -0.1222 | 0.0894 | -0.0618 | -0.0504 | -0.0540 | 0.0512 | -0.0207 | -0.0272 | -0.0219 |
| AC | *L. glaber* (Thunb.) Nakai | 0.0109 | -0.4757 | 0.1320 | -0.0141 | -0.0170 | -0.0996 | 0.1168 | -0.0768 | -0.0046 | -0.1179 | 0.1170 | -0.0023 | 0.0049 | -0.0435 | 0.0880 | -0.0064 | 0.0213 | 0.0081 |
| AC | *L. glutinosus* (Blume) Soepadmo | 0.0672 | -0.4838 | -0.0348 | -0.0089 | 0.0601 | -0.2306 | -0.0091 | 0.0003 | 0.0643 | -0.1316 | 0.0158 | 0.0062 | 0.0516 | -0.0781 | 0.0066 | -0.0117 | 0.0436 | -0.0470 |
| AC | *L. gracilis* (Korth.) Soepadmo | 0.0731 | -0.3406 | -0.2680 | -0.0601 | 0.1029 | -0.3614 | 0.0090 | 0.0497 | -0.0566 | -0.0317 | -0.1545 | -0.0746 | 0.0514 | -0.0443 | -0.1036 | 0.0195 | -0.0335 | -0.0166 |
| AC | *L. hancei* (Benth.) Rehder | -0.1556 | -0.4831 | -0.0670 | 0.0095 | 0.0110 | -0.2474 | 0.0408 | -0.0230 | -0.0495 | -0.1416 | 0.0382 | -0.0307 | -0.0574 | -0.0959 | 0.0454 | -0.0256 | -0.0420 | -0.0750 |
| AC | *L. handelianus* A. Camus | -0.0949 | -0.4713 | -0.0438 | 0.0212 | -0.0852 | -0.2477 | 0.0036 | 0.0112 | -0.0192 | -0.0841 | -0.0053 | 0.0066 | 0.0018 | -0.0842 | 0.0857 | -0.0239 | -0.0142 | -0.0182 |
| AC | *L. harlandii* (Hance ex Walp.) Rehder | -0.1281 | -0.4809 | 0.0567 | -0.0068 | -0.0552 | -0.2105 | 0.1255 | -0.1790 | -0.1550 | -0.0342 | 0.0868 | -0.0587 | -0.0211 | -0.0435 | 0.0082 | -0.0962 | -0.0264 | -0.0356 |
| AC | *L. henryi* (Seemen) Rehder & E.H. Wilson | -0.0914 | -0.5083 | -0.1749 | 0.0450 | -0.0833 | -0.1995 | 0.0607 | -0.0253 | -0.0685 | -0.1104 | 0.0857 | -0.0457 | -0.0762 | -0.0959 | 0.0494 | -0.0384 | -0.0615 | -0.0568 |
| AC | *L. himalaicus* C. C.Huang & Y.T. Chang | -0.1027 | -0.4692 | -0.0809 | 0.0078 | -0.0168 | -0.2593 | -0.0229 | 0.0181 | -0.0240 | -0.1425 | 0.0152 | -0.0299 | 0.0128 | -0.1191 | 0.0175 | -0.0035 | -0.0155 | -0.0844 |
| AC | *L. howii* Chun | 0.1913 | -0.4431 | 0.1306 | -0.0048 | 0.0631 | -0.2071 | -0.0156 | -0.0689 | 0.0095 | -0.1163 | -0.0594 | -0.0237 | -0.0016 | -0.0804 | -0.0387 | -0.0043 | 0.0082 | -0.0349 |
| AC | *L. hypoglaucus* (Hu) C.C. Huang ex Y.C. Hsu & H.W. Jen | -0.1346 | -0.5127 | 0.0110 | -0.0083 | -0.0730 | -0.2360 | 0.0070 | 0.0140 | -0.0928 | -0.1051 | 0.0215 | 0.0010 | -0.0257 | -0.0440 | 0.0439 | -0.0200 | -0.0093 | -0.0091 |
| AC | *L. indutus* (Blume) Rehder * | -0.1821 | -0.3778 | -0.0992 | 0.0493 | -0.1340 | -0.1409 | 0.0058 | -0.0125 | -0.0556 | -0.0915 | 0.0589 | -0.0736 | 0.0112 | 0.0311 | -0.0180 | -0.0312 | -0.0390 | 0.0222 |
| AC | *L. jacobsii* Soepadmo | -0.3331 | -0.3256 | 0.0484 | -0.2294 | -0.2002 | 0.0519 | -0.0642 | -0.0647 | -0.0694 | 0.0707 | -0.0889 | -0.0072 | 0.0199 | 0.1143 | -0.0055 | 0.0407 | 0.0447 | -0.0024 |
| ER | *L. javensis* Blume | 0.0074 | -0.1324 | -0.3591 | -0.0998 | 0.1415 | -0.3156 | -0.0565 | 0.0590 | 0.0049 | -0.2070 | 0.2120 | -0.0028 | 0.0707 | 0.1113 | -0.2202 | 0.0021 | 0.0845 | -0.2525 |
| AC | *L. kawakamii* (Hayata) Hayata | 0.0110 | -0.4911 | -0.0759 | 0.0120 | -0.0600 | -0.2212 | -0.0016 | 0.0142 | 0.0159 | -0.1331 | 0.0686 | -0.0085 | 0.0097 | -0.1114 | 0.0455 | -0.0496 | 0.0078 | -0.0754 |
| AC | *L. konishii* (Hayata) Hayata * | -0.1526 | -0.5374 | -0.0320 | 0.0255 | -0.1198 | -0.1838 | 0.2466 | -0.2213 | 0.0251 | 0.0063 | 0.0424 | -0.0532 | -0.0650 | -0.0175 | 0.0326 | -0.0987 | 0.0580 | 0.0017 |
| ER | *L. lampadarius* (Gamble) A. Camus * | 0.1754 | -0.4440 | -0.0454 | 0.0120 | 0.0350 | -0.2207 | 0.0641 | 0.0286 | 0.0214 | -0.0785 | 0.0526 | -0.0756 | 0.0842 | 0.0787 | -0.0070 | -0.1199 | 0.0290 | 0.0071 |
| ER | *L. laoticus* (Hhickel & A. Camus) A. Camus | -0.1350 | -0.4935 | -0.0441 | -0.0038 | -0.0726 | -0.2614 | 0.0816 | -0.0026 | -0.1104 | -0.0819 | 0.0486 | -0.0676 | -0.0600 | -0.0504 | 0.0596 | -0.0832 | -0.0468 | -0.0222 |
| AC | *L. lappaceus* (Roxb.) Rehder | 0.0234 | -0.4882 | 0.0516 | 0.0179 | 0.0202 | -0.2231 | 0.0296 | 0.0098 | 0.0567 | -0.0692 | -0.0216 | 0.0124 | 0.0575 | -0.0183 | -0.0377 | 0.0453 | 0.0232 | 0.0000 |
| ER | *L. lepidocarpus* (Hayata) Hayata | -0.1618 | -0.5395 | 0.0033 | 0.0123 | -0.0990 | -0.2220 | 0.0665 | -0.0276 | -0.0832 | -0.0846 | 0.0732 | -0.0792 | -0.0538 | -0.0459 | 0.0389 | -0.0969 | -0.0150 | -0.0141 |
| AC | *L. leptogyne* (Korth.) Soepadmo | -0.0246 | -0.4584 | -0.0662 | 0.0158 | -0.0729 | -0.2283 | -0.0698 | 0.0124 | -0.0432 | -0.1616 | -0.0006 | 0.0039 | -0.0263 | -0.0597 | -0.0179 | 0.0248 | -0.0647 | -0.0415 |
| AC | *L. lindleyanus* (Wall. ex A. DC.) A. Camus | -0.2044 | -0.4260 | 0.0694 | -0.0308 | -0.0230 | -0.1979 | 0.0676 | -0.1421 | -0.0544 | -0.0025 | 0.0352 | -0.1222 | -0.0106 | -0.0138 | -0.0075 | -0.0978 | 0.0036 | -0.0203 |
| AC | *L. litseifolius* (Hance) Chun | -0.1436 | -0.5279 | -0.0602 | 0.0390 | -0.0989 | -0.1615 | 0.0687 | -0.0555 | -0.0480 | -0.0187 | 0.0850 | -0.1536 | -0.0474 | 0.0036 | 0.0311 | -0.1331 | -0.0459 | -0.0147 |
| AC | *L. longanoides* C.C. Huang & Y.T. Chang | 0.0390 | -0.4869 | 0.0490 | 0.0065 | 0.0152 | -0.2099 | -0.0074 | 0.0036 | -0.0124 | -0.1363 | -0.0006 | -0.0070 | -0.0118 | -0.1033 | 0.0100 | 0.0032 | -0.0105 | -0.0697 |
| AC | *L. longipedicellatus* (Hickel & A. Camus) A. Camus | -0.0941 | -0.5063 | -0.1067 | 0.0262 | -0.0226 | -0.2303 | 0.0181 | -0.0109 | -0.0236 | -0.1522 | 0.0824 | -0.0521 | -0.0603 | -0.0924 | 0.0786 | -0.0451 | -0.0566 | -0.0564 |
| AC | *L. lucidus* (Roxb.) Rehder | -0.1193 | -0.3185 | -0.2866 | 0.1980 | -0.1626 | -0.1872 | 0.0157 | -0.0903 | -0.0354 | -0.1108 | 0.1173 | -0.1297 | 0.0465 | 0.0242 | -0.0326 | -0.0716 | -0.0533 | -0.0402 |
| AC | *L. luteus* Soepadmo | 0.2319 | -0.1081 | 0.2949 | 0.0234 | -0.0012 | -0.2371 | -0.0669 | 0.3132 | 0.1149 | -0.2848 | 0.0710 | 0.1041 | -0.0626 | 0.1338 | 0.0890 | -0.0774 | -0.1407 | 0.1128 |
| AC | *L. mairei* (Schottky) Rehder | -0.0380 | -0.4963 | 0.0329 | 0.0038 | -0.0488 | -0.1832 | 0.0355 | 0.0076 | 0.0121 | -0.0795 | 0.0660 | -0.0230 | 0.0125 | -0.0405 | 0.0632 | -0.0396 | 0.0068 | -0.0235 |
| ER | *L. megacarpus* Soepodmo | -0.5704 | 0.0342 | -0.0088 | -0.0153 | 0.0414 | 0.2398 | 0.0872 | -0.1552 | 0.0434 | -0.0217 | -0.1220 | -0.0686 | -0.0028 | -0.0205 | -0.0132 | 0.0842 | 0.0476 | -0.0171 |
| AC | *L. meijeri* Soepadmo | 0.1227 | -0.3875 | -0.1012 | -0.0252 | 0.0805 | -0.2079 | -0.0735 | -0.1037 | 0.1192 | -0.1828 | 0.0504 | -0.0245 | 0.1164 | 0.0106 | -0.1003 | -0.0316 | -0.0029 | -0.0456 |
| AC | *L. naiadarum* (Hance) Chun | -0.0466 | -0.5085 | -0.0883 | 0.0072 | -0.0367 | -0.1750 | 0.0294 | -0.0287 | -0.0693 | -0.0845 | 0.0782 | -0.0358 | -0.0612 | -0.0865 | 0.0454 | -0.0302 | -0.0264 | -0.0555 |
| AC | *L. nieuwenhuisii* (Seemen) A. Camus | 0.0663 | -0.4431 | 0.1070 | 0.0177 | 0.0249 | -0.1153 | 0.1126 | 0.1432 | 0.1191 | -0.0455 | 0.0516 | 0.0468 | 0.0446 | -0.0234 | -0.0115 | 0.0474 | 0.0228 | 0.0034 |
| AC | *L. nodosus* Soepadmo | 0.0043 | -0.4966 | -0.0113 | -0.0028 | 0.0139 | -0.2561 | -0.0220 | 0.0126 | 0.0017 | -0.1377 | -0.0227 | -0.0116 | 0.0102 | -0.0941 | -0.0083 | 0.0110 | -0.0094 | -0.1069 |
| AC | *L. oblanceolatus* C.C. Huang & Y.T. Chang | -0.1942 | -0.4766 | 0.0152 | -0.0059 | -0.0845 | -0.1393 | 0.0646 | -0.0536 | -0.0402 | -0.0836 | 0.0405 | -0.0578 | -0.0459 | -0.0453 | 0.0152 | -0.0644 | -0.0283 | -0.0306 |
| AC | *L. obscurus* C.C. Huang & Y.T. Chang | -0.0406 | -0.5084 | -0.0824 | 0.0039 | 0.0009 | -0.2493 | -0.0557 | 0.0013 | -0.0033 | -0.1509 | -0.0072 | 0.0018 | -0.0015 | -0.1075 | 0.0168 | 0.0013 | 0.0013 | -0.0868 |
| ER | *L. pachylepis* A. Camus | -0.0586 | -0.4524 | -0.2694 | 0.0797 | -0.1526 | -0.3472 | 0.2243 | -0.1287 | 0.0054 | -0.0462 | 0.0346 | 0.0054 | -0.0836 | -0.0653 | 0.0991 | -0.1227 | 0.0206 | -0.0141 |
| ER | *L. pachyphyllus* (Kruz) Rehder | -0.2040 | -0.5510 | 0.0274 | -0.0040 | -0.1501 | -0.1597 | 0.1518 | -0.1674 | -0.0100 | -0.0345 | 0.0567 | -0.0965 | 0.0229 | 0.0086 | -0.0074 | -0.1056 | 0.0659 | -0.0271 |
| AC | *L. pallidus* (Blume) Rehder | -0.5515 | 0.0268 | -0.1506 | -0.1189 | 0.0197 | 0.2879 | 0.0252 | -0.0811 | 0.0435 | -0.0030 | -0.1341 | -0.0840 | -0.0144 | -0.0622 | -0.1014 | 0.0921 | -0.0458 | 0.0286 |
| AC | *L. petelotii* A. Camus | -0.1559 | -0.4841 | -0.0195 | 0.0195 | -0.0742 | -0.1574 | 0.0618 | -0.0157 | -0.0267 | -0.0926 | 0.0445 | -0.1038 | -0.0832 | -0.0270 | 0.0178 | -0.0949 | -0.0437 | 0.0215 |
| ER | *L. platycarpus* (Blume) Rehder | -0.3806 | -0.1890 | 0.0280 | -0.2579 | -0.2739 | 0.1327 | 0.0208 | -0.0216 | -0.0627 | 0.0902 | -0.0273 | 0.0311 | 0.1385 | 0.1101 | 0.0470 | -0.1014 | 0.0191 | -0.0644 |
| AC | *L. polystachyus* (Wall. ex A. DC.) Rehder | 0.0306 | -0.4903 | 0.0227 | -0.0059 | -0.0069 | -0.1403 | 0.0472 | 0.0177 | -0.0171 | -0.0928 | 0.0699 | -0.0047 | 0.0001 | -0.0650 | 0.0651 | -0.0132 | -0.0050 | -0.0394 |
| AC | *L. pseudokunstleri* A. Camus | 0.3499 | 0.2760 | 0.1571 | -0.0031 | -0.2587 | 0.2167 | 0.1496 | -0.2386 | 0.0758 | -0.1127 | -0.0426 | -0.1177 | 0.1255 | 0.0368 | -0.0825 | 0.0002 | -0.1026 | -0.0138 |
| AC | *L. pseudomoluccus* (Blume) Rehder | -0.0848 | -0.1787 | -0.3545 | -0.4409 | 0.1941 | -0.0205 | -0.0744 | -0.1214 | -0.0180 | 0.0819 | -0.0557 | -0.0165 | -0.0717 | -0.0567 | 0.0495 | -0.0222 | -0.0072 | -0.1074 |
| AC | *L. pseudovestitus* A. Camus | 0.0356 | -0.4796 | -0.0592 | -0.0126 | -0.0631 | -0.1855 | 0.0873 | -0.0325 | -0.0872 | -0.1076 | 0.0975 | 0.0426 | -0.0332 | -0.0950 | 0.0408 | -0.0319 | 0.0076 | -0.0368 |
| ER | *L. pseudoxizangensis* Z.K. Zhou & H. Sun | -0.1522 | -0.5420 | -0.0054 | 0.0096 | -0.0779 | -0.2356 | 0.1087 | -0.0775 | -0.1190 | -0.0678 | 0.0592 | -0.1116 | -0.0562 | -0.0244 | 0.0212 | -0.0700 | -0.0103 | -0.0002 |
| AC | *L. pusillus* Soepadmo | -0.4907 | 0.1236 | -0.2333 | -0.0563 | 0.2315 | 0.1452 | -0.0388 | -0.0739 | -0.0075 | -0.1470 | -0.0857 | 0.0034 | -0.1425 | -0.0476 | 0.0743 | 0.0318 | -0.0126 | 0.0433 |
| AC | *L. rhabdostachyus* (Hickel & A. Camus) A. Camus | -0.1288 | -0.4678 | -0.0063 | -0.0115 | -0.0927 | -0.2129 | 0.0264 | -0.0034 | -0.0245 | -0.1284 | 0.0228 | -0.0110 | -0.0305 | -0.1084 | 0.0325 | -0.0378 | -0.0415 | -0.0815 |
| AC | *L. rosthornii* (Schottky) Barnett | -0.0828 | -0.5110 | 0.0284 | 0.0095 | 0.0462 | -0.2294 | -0.0339 | 0.0018 | 0.0245 | -0.1379 | -0.0160 | 0.0013 | 0.0337 | -0.0907 | -0.0089 | 0.0001 | 0.0382 | -0.0678 |
| AC | *L. silvicolarum* (Hance) Chun | -0.2451 | -0.4597 | -0.0693 | 0.0366 | -0.1156 | -0.2152 | -0.0136 | 0.0441 | -0.0339 | -0.1223 | 0.0320 | -0.0193 | -0.0211 | -0.0959 | 0.0222 | -0.0675 | -0.0361 | -0.0745 |
| AC | *L. skanianus* (Dunn) Rehder | 0.0947 | -0.4769 | -0.0054 | 0.0051 | 0.0080 | -0.2383 | -0.0537 | 0.0098 | -0.0047 | -0.1518 | -0.0207 | -0.0026 | 0.0112 | -0.1081 | -0.0078 | 0.0049 | 0.0207 | -0.0757 |
| AC | *L. sundaicus* (Blume) Rehder | -0.1484 | -0.4689 | -0.0439 | 0.0034 | -0.1205 | -0.2456 | -0.0303 | 0.0534 | -0.0775 | -0.0829 | 0.0161 | 0.0145 | -0.0405 | -0.0308 | 0.0372 | -0.0483 | 0.0027 | -0.0175 |
| AC | *L. taitoensis* (Hayata) Hayata | -0.0425 | -0.5151 | -0.0914 | 0.0043 | -0.0849 | -0.1975 | 0.1019 | -0.0210 | -0.0349 | -0.1304 | 0.1069 | -0.0146 | -0.0564 | -0.1034 | 0.0146 | -0.0612 | -0.0463 | -0.0454 |
| AC | *L. touranensis* (Hickel & A. Camus) A. Camus | -0.0101 | -0.4946 | 0.0565 | -0.0121 | -0.0413 | -0.2235 | -0.0423 | 0.0247 | -0.0264 | -0.1505 | 0.0421 | -0.0132 | -0.0495 | -0.0952 | 0.0458 | -0.0123 | -0.0381 | -0.0718 |
| AC | *L. trachycarpus* (Hickel & A.Camus) A. Camus | -0.0374 | -0.4771 | -0.0527 | 0.0048 | -0.0820 | -0.2687 | 0.0286 | -0.0126 | -0.0381 | -0.1604 | 0.0806 | -0.0409 | -0.0768 | -0.0764 | 0.0425 | -0.0274 | -0.0549 | -0.0545 |
| ER | *L. truncatus* (King ex Hook. f.) Rheder | -0.0966 | -0.5509 | -0.0252 | 0.0059 | -0.0567 | -0.2375 | 0.0852 | -0.0392 | -0.0836 | -0.0941 | 0.0740 | -0.0606 | -0.0384 | -0.0534 | 0.0703 | -0.0801 | -0.0289 | -0.0174 |
| ER | *L. turbinatus* (Stapf) Forman | -0.4082 | -0.3853 | 0.0637 | -0.2029 | -0.0603 | -0.0027 | -0.0643 | -0.1180 | 0.0749 | -0.0691 | -0.0085 | 0.0077 | 0.0000 | -0.0548 | 0.0530 | -0.0146 | -0.0557 | -0.0524 |
| ER | *L. uvariifolius* (Hance) Rehder | -0.1899 | -0.5632 | -0.0511 | 0.0305 | -0.1522 | -0.1788 | 0.1059 | -0.1296 | -0.0296 | -0.0079 | 0.0528 | -0.1534 | 0.0132 | -0.0133 | 0.0068 | -0.1414 | 0.0374 | -0.0022 |
| ER | *L. variolosus* (Franch.) Chun | -0.0734 | -0.5314 | -0.0842 | 0.0235 | -0.0782 | -0.2479 | 0.0363 | -0.0196 | -0.0637 | -0.1370 | 0.0514 | -0.0312 | -0.0277 | -0.1068 | 0.0595 | -0.0313 | -0.0613 | -0.0618 |
| AC | *L. vestitus* (Hickel & A. Camus) A. Camus | -0.1673 | -0.4627 | 0.0047 | -0.0088 | -0.1323 | -0.1774 | 0.0384 | -0.0324 | -0.0082 | -0.0340 | 0.0346 | -0.0668 | 0.0281 | -0.0318 | -0.0041 | -0.1197 | 0.0141 | -0.0474 |
| ER | *L. xylocarpus* (Kurz) Markgr. | -0.0969 | -0.5477 | -0.0979 | 0.0290 | -0.1331 | -0.2178 | 0.0663 | -0.0424 | -0.0748 | -0.1225 | 0.0588 | -0.0532 | -0.0736 | -0.0503 | 0.0471 | -0.0684 | -0.0345 | -0.0325 |
